# Australian guideline on offloading treatment for foot ulcers: part of the 2021 Australian evidence-based guidelines for diabetes-related foot disease

**DOI:** 10.1186/s13047-022-00538-3

**Published:** 2022-05-05

**Authors:** Malindu E. Fernando, Mark Horsley, Sara Jones, Brian Martin, Vanessa L. Nube, James Charles, Jane Cheney, Peter A. Lazzarini, on behalf of the Australian Diabetes-related Foot Disease Guidelines & Pathways Project

**Affiliations:** 1 Queensland Research Centre for Peripheral Vascular Disease, College of Medicine and Dentistry, James Cook University, Townsville, Australia; 2 Faculty of Health and Medicine, School of Health Sciences, University of Newcastle, Callaghan, Australia; 3 Department of Orthopaedics, Royal Prince Alfred Hospital, Sydney, Australia; 4 Department of Rural Health, University of South Australia, Adelaide, Australia; 5 Department of Orthopaedics, Nepean Hospital, Sydney, Australia; 6 Sydney Local Health District, Department of Podiatry, Sydney, Australia; 7 First Peoples Health Unit, Faculty of Health, Griffith University, Gold Coast, Queensland Australia; 8 Diabetes Victoria, Melbourne, Australia; 9 School of Public Health and Social Work, Queensland University of Technology, Brisbane, Australia; 10 Allied Health Research Collaborative, The Prince Charles Hospital, Brisbane, Australia; 11 Diabetes Feet Australia, Brisbane, Australia; 12 Australian Diabetes Society, Sydney, Australia

**Keywords:** Cast, Diabetes-related foot ulceration, Diabetic foot, Footwear, Foot ulcer, Guidelines, Offloading, Offloading device, Surgery, Treatment

## Abstract

**Background:**

Pressure offloading treatment is critical for healing diabetes-related foot ulcers (DFU). Yet the 2011 Australian DFU guidelines regarding offloading treatment are outdated. A national expert panel aimed to develop a new Australian guideline on offloading treatment for people with DFU by adapting international guidelines that have been assessed as suitable to adapt to the Australian context.

**Methods:**

National Health and Medical Research Council procedures were used to adapt suitable International Working Group on the Diabetic Foot (IWGDF) guidelines to the Australian context. We systematically screened, assessed and judged all IWGDF offloading recommendations using best practice ADAPTE and GRADE frameworks to decide which recommendations should be adopted, adapted or excluded in the Australian context. For each recommendation, we re-evaluated the wording, quality of evidence, strength of recommendation, and provided rationale, justifications and implementation considerations, including for geographically remote and Aboriginal and Torres Strait Islander peoples. This guideline, along with five accompanying Australian DFU guidelines, underwent public consultation, further revision and approval by ten national peak bodies (professional organisations).

**Results:**

Of the 13 original IWGDF offloading treatment recommendations, we adopted four and adapted nine. The main reasons for adapting the IWGDF recommendations included differences in quality of evidence ratings and clarification of the intervention(s) and control treatment(s) in the recommendations for the Australian context. For Australians with plantar DFU, we recommend a step-down offloading treatment approach based on their contraindications and tolerance. We strongly recommend non-removable knee-high offloading devices as first-line treatment, removable knee-high offloading devices as second-line, removable ankle-high offloading devices third-line, and medical grade footwear as last-line. We recommend considering using felted foam in combination with the chosen offloading device or footwear to further reduce plantar pressure. If offloading device options fail to heal a person with plantar DFU, we recommend considering various surgical offloading procedures. For people with non-plantar DFU, depending on the type and location of the DFU, we recommend using a removable offloading device, felted foam, toe spacers or orthoses, or medical grade footwear. The six new guidelines and the full protocol can be found at: https://diabetesfeetaustralia.org/new-guidelines/.

**Conclusions:**

We have developed a new Australian evidence-based guideline on offloading treatment for people with DFU that has been endorsed by ten key national peak bodies. Health professionals implementing these offloading recommendations in Australia should produce better DFU healing outcomes for their patients, communities, and country.

**Supplementary Information:**

The online version contains supplementary material available at 10.1186/s13047-022-00538-3.

## Background

Diabetes-related foot ulcers (DFU) are a leading cause of the global hospitalisation, disability and healthcare costs burdens [1–4]. In Australia each year, DFU affects an estimated 50,000 people, resulting in around 30,000 hospitalisations, 5000 amputations and nearly $AU2 billion in health system costs [3–6]. Aboriginal and Torres Strait Islander peoples have up to a 38-fold risk of developing DFU and amputation compared with non-Indigenous people in Australia [3, 6, 7]. Thus, improved care for Australians with DFU is critical to reducing a large cause of the national healthcare burden and to closing the gap in health inequality experienced by Aboriginal and Torres Strait Islanders [3, 6, 8].

The most common pathway to developing a DFU is via high plantar tissue stress (due to high plantar pressure and/or high activity) on the foot of a person with a loss of protective sensation due to diabetes-related peripheral neuropathy (DPN) [1, 3, 9]. Plantar tissue stress is the result of an accumulation of the repetitive cycles of plantar pressure and shear pressure during daily weight-bearing activity [1, 9, 10]. DPN not only causes a loss of protective sensation but can also result in higher plantar tissue stress due to detrimental changes in gait, soft tissue and foot deformities [1, 9, 10]. High plantar tissue stress if left untreated leads to subcutaneous tissue damage and eventually a DFU develops [1, 9, 10]. Thus, reducing high plantar tissue stress that caused the DFU, or reducing high tissue stress in DFUs from other causes such as ill-fitting footwear, is critical to healing people with DFU.

Optimal treatment for most effective DFU healing involves a multi-disciplinary team of different health professionals, in collaboration with the patient (person affected by DFU), that collectively address the multiple factors contributing to the DFU aetiology by managing multiple aspects of the wound including infection, ischaemia and plantar tissue stress [1, 9, 11]. Pressure offloading aims to reduce high plantar tissue stress and has been found to be critical to achieve timely and complete DFU healing [1, 9, 11]. To do this effectively, offloading should maximise the desirable effects (benefits) of reducing high plantar tissue stress; whilst also minimising any undesirable effects (risks), such as adverse events and high costs [10, 12, 13]. Various offloading treatments have been used clinically, including offloading devices, footwear and corrective surgery [12, 14]. Yet, these different offloading treatments carry differing benefits and risks [9, 15, 16], quality of supporting evidence [9, 15, 16] and feasibility of clinical uptake [13, 17–19], making the clinical decision for offloading treatments in people with DFU complex.

Evidence-based guidelines have been previously developed to weigh up the benefits, risks, quality of evidence and feasibility of treatments to provide health professionals with best practice recommendations on optimal treatments for people with DFU [16, 20]. However, the current 2011 Australian evidence-based DFU guidelines are outdated [3, 16, 21] and have not weighed up the substantial new offloading evidence published over the last decade [15]. Conversely, many international evidence-based DFU guidelines have recently been published [9, 22–24], but their applicability and acceptability to the Australian context is unclear. Specifically, the methodological quality, suitability and currency of international guidelines and their relevance to the unique Australian health context needs formal assessment before they can be used, adopted, or adapted in Australia. Thus, we aimed to systematically assess, adopt, or adapt suitable international guidelines to the Australian context to become the new Australian evidence-based guideline on offloading treatment for people with DFU.

## Methods

The methodology for this guideline followed the recommended National Health and Medical Research Council (NHMRC) procedures for adapting source guidelines [25–27] and has been described in detail in an accompanying guidelines development protocol paper [28]. The development protocol reports that the 2019 International Working Group on the Diabetic Foot (IWGDF) guidelines were systematically identified and assessed as suitable international source guidelines to adapt for this new guideline [28]. Thus, the subsequent steps for adapting the IWGDF guideline to the Australian context for offloading treatment in people with DFU are summarised below.

### National panel

A national expert panel (referred to as “the panel”) was established by the Australian DFD Guidelines development working group to develop and author this Australian offloading guideline, and was comprised of recognised multi-disciplinary (inter) national experts in surgical and non-surgical offloading treatments for people with DFU, and consumer and Aboriginal and Torres Strait Islander representatives with expertise in DFD [28]. The panel was provided with all offloading recommendations (and supporting rationale and evidence) from the IWGDF guidelines [15, 22] as the basis for developing this guideline [28].

### Screening recommendations

The initial step for the panel involved using a customised 7-item ADAPTE evaluation form [26, 28] to screen each IWGDF offloading recommendation (and rationale) for their quality of evidence, strength of recommendation, acceptability and feasibility in the Australian context. Any recommendation in which the panel by consensus were certain that all items agreed with the IWGDF quality of evidence and strength of recommendation ratings and were acceptable and applicable in the Australian national context, were adopted for the Australian context. Whereas any recommendation where the panel did not agree or were unsure on any of these items was fully assessed [26, 28].

### Assessing recommendations

The second step involved using a customised GRADE Evidence to Decision (EtD) template tool [27–30] to systematically evaluate all the evidence supporting those recommendations (and all rationale) needing full assessment. This was performed by one panel member, checked by a second, who extracted and populated the EtD tool with all supporting text for the recommendation included in the IWGDF offloading guideline and systematic review [15, 22]. Eight important EtD criteria were specifically populated: the problem (a priority), values (of outcomes), desirable effects, undesirable effects, balance of effects, quality of (supporting) evidence, acceptability and feasibility [27–30]. The panel then reviewed, discussed and made consensus judgement decisions on all eight EtD criteria [29, 30] and compared their judgements for these criteria with those from the IWGDF [27, 28].

### Decisions on recommendations

Based on the level of agreement between the panel and IWGDF judgements, the next step involved the panel making a consensus decision on whether to adopt, adapt or exclude each recommendation for the Australian context [27, 28]. These decisions were defined as: adopted, if there were no major differences between the panel and the IWGDF judgements; adapted, if there were differences; and excluded, if there were substantial differences and/or the panel concluded the recommendation was not acceptable or applicable in Australia [27, 28]. The recommendations in which the panel decided to adapt then had their quality of evidence rating, strength of recommendation rating [29, 30] and written recommendation re-evaluated via consensus based on the panel’s judgements [27, 28]. The panel rated the quality of evidence in alignment with the GRADE system as High, Moderate, Low or Very Low, based on the panel’s confidence that the findings were from studies that reported consistent effects with low risk of bias and further research was unlikely to change that confidence [29, 30]. The panel also rated the strength of recommendation in alignment with the GRADE system, based on weighing up the balance of effects, quality of evidence, applicability and feasibility [29, 30] in the Australian context [28] as: Strong, if there was a large clear difference in the balance of effects between an intervention and control; or Weak, if there was a small and/or uncertain difference [29, 30].

### Drafting recommendations

The final step involved re-drafting the guideline recommendations and reasons for the Australian context [28]. The panel re-wrote any adapted recommendation to be clear, specific, and unambiguous as per the GRADE system [27, 29, 30]. For each recommendation the panel drafted the following reasons for the Australian context: the clinical question originally posed; the recommendation(s) to address that question; the rationale for the decision to adopt, adapt or exclude the original IWGDF recommendation; justifications for the recommendation (and detailed justifications if the recommendation was fully assessed); and implementation considerations for the recommendation in Australia (including a description of the treatment, any contraindications, procedures, monitoring and special considerations for geographically remote and Aboriginal and Torres Strait Islander people) [27–30]. The panel collated all recommendations (and reasons), along with suggested future research priorities, into a consultation draft manuscript of the Australian evidence-based guideline on offloading treatment for people with DFU ready for public consultation [28]. The finalised recommendations were also developed into an Australian clinical pathway for offloading treatment, using best practice methodology for developing pathways, to help facilitate implementation of these new evidence-based recommendations [31].

### Consultation and endorsement

The consultation draft of this Australian offloading guideline manuscript underwent a formal six-week public consultation period using a 23-item customised consultation survey. The survey was based on ADAPTE examples with additional open ended items for feedback on each recommendation and overall final thoughts [26, 28]. Each item employed a 5-point Likert scale from strongly agree to strongly disagree in response to a statement as the answer options for each item. All survey and written feedback formally submitted from the consultation period was collated, analysed and the guideline was subsequently revised accordingly by the authors [26, 28]. All de-identified formal feedback and the authors individual responses to the feedback were collated and publicly posted on the Diabetes Feet Australia website. Finally, the authors sought endorsement from the Australian DFD Guidelines development working group and relevant national peak bodies (also known as national professional organisations, national professional societies or national representative bodies, amongst other terms in other nations) [28]. We refer the reader to the results section below for all final recommendations contained in the new Australian evidenced-based guidelines on offloading treatment for people with DFU. The results and recommendations in our below Australian guideline should be read in conjunction with the respective IWGDF source guideline and systematic review from the IWGDF Offloading Working Group for full descriptions of findings and rationale [15, 22].

## Results

Following screening of all 13 IWGDF offloading recommendations, four were adopted and nine required further full assessment (Table 1). Of the nine recommendations which underwent full assessment, all were adapted to the Australian context for the following reasons: six had their quality of evidence rating downgraded (Australian Recommendations 1a, 1b, 3, 4, 5, 9), six added the comparison control treatment (1a, 2, 3, 4, 5, 9), three adapted the intervention(s) (5, 6a, 9), one adapted the population (6b), and one had strength of recommendation rating downgraded (1b) (Tables 2 and 3). A summary of the wording differences between the new Australian recommendations and the original IWGDF recommendations can be found in Table 3.

**Table 1:** Summary of screening ratings for acceptability and applicability in the Australian context for all the IWGDF Offloading recommendations

Recommendation	Acceptability			Applicability				Full assessment	Comments
	1	2	3	4	5	6	7		
1a	+	?	+	+	+	?	+	Yes	Assess strength of recommendation & expertise availability
1b	+	?	?	+	?	?	?	Yes	Assess patient preference, equipment availability, expertise availability & legislative/policy constraints
2	+	?	+	+	+	+	+	Yes	Assess strength of recommendation
3	?	–	+	+	+	+	+	Yes	Assess quality of evidence & strength of recommendation
4a	?	–	?	+	?	+	?	Yes	Assess quality of evidence, strength of recommendation, patient preference, equipment availability & legislative/policy constraints
4b	?	+	+	+	+	+	+	Yes	Assess quality of evidence
5	+	?	?	+	+	?	+	Yes	Assess strength of recommendation & expertise availability
6	+	?	+	+	+	+	+	Yes	Assess strength of recommendation
7a	+	+	+	+	+	+	+	No	
7b	+	+	+	+	+	+	+	No	
7c	+	+	+	+	+	+	+	No	
8	+	+	+	+	+	+	+	No	
9	?	–	+	+	+	+	+	Yes	Assess quality of evidence, strength of recommendation
Total	**9**	**5**	**10**	**13**	**11**	**10**	**11**	**9**	
%	**69%**	**38%**	**77%**	**100%**	**85%**	**77%**	**85%**	**69%**	

Note: +, yes item is met; −, no item is not met;? unsure if item is met

**Table 2:** Summary of final panel judgements compared with the IWGDF judgements for all the IWGDF Offloading recommendations

No.	Problem	Desirable effects	Undesirable effects	Quality of evidence	Values	Balance of effects	Acceptability	Applicability/ feasibility	Decision	Comments
1a	+ Yes	? Moderate	+ Trivial	- Moderate	+ Probably no important uncertainty	+ Favours the intervention	+ Probably yes	+ Probably yes	Adapt	Adapted QoE & control
1b	+ Yes	? Trivial	+ Trivial	- Low	+ Probably no important uncertainty	+ Does not favour either intervention or control	+ Probably yes	+ Probably yes	Adapt	Adapted QoE & strength of recommendation
2	+ Yes	+ Moderate	? Varies	+ Low	- Possibly important uncertainty	+ Probably favours the intervention	+ Varies	+ Probably yes	Adapt	Adapted control, patient circumstances & foot-device interface
3	+ Yes	? Varies	+ Small	- Very low	+ Probably no important uncertainty	+ Favours the intervention	+ Yes	+ Yes	Adapt	Adapted QoE, control, patient circumstances & foot-device interface
4a	+ Yes	? Don’t know	? Don’t know	- Low	- Possibly important uncertainty	+ Favours the comparison	+ Probably yes	+ Probably yes	Adapt	Adapt QoE & control
4b	+ Yes	+ Small	+ Small	- Very low	+ Probably no important uncertainty	+ Probably favours the intervention	+ Probably yes	+ Probably yes	Adapt	Adapted QoE, intervention & control
5	+ Yes	+ Moderate	+ Small	+ Low	+ Probably no important uncertainty	+ Probably favours the intervention	+ Probably yes	? Probably yes	Adapt	Adapted intervention
6	+ Probably yes	+ Moderate	+ Small	+ Low	+ Probably no important uncertainty	+ Probably favours the intervention	+ Probably yes	? Yes	Adapt	Adapted population
7a	=	=	=	=	=	=	=	=	Adopt	Adopted in screening
7b	=	=	=	=	=	=	=	=	Adopt	Adopted in screening
7c	=	=	=	=	=	=	=	=	Adopt	Adopted in screening
8	=	=	=	=	=	=	=	=	Adopt	Adopted in screening
9	+ Yes	- Don’t know	- Don’t know	- Very low	+ Probably no important uncertainty	+ Favours the intervention	+ Probably yes	+ Probably yes	Adapt	Adapted QoE, intervention & control

Note: +, panel agreed with the original IWGDF judgement; −, panel disagreed with the original IWGDF judgement; ?, panel unsure if agreed with the original IWGDF judgement due to lack of IWGDF information on judgement; =, panel agreed with the original IWGDF judgement during screening (see Table 1); *QoE* Quality of evidence

**Table 3:** Summary of the original IWGDF recommendation compared with the new Australian guideline recommendations for offloading

No.	Original IWGDF Recommendation	Decision	No.	New Australian Recommendation
1a	In a person with diabetes and a neuropathic plantar forefoot or midfoot ulcer, use a non-removable knee-high offloading device with an appropriate foot-device interface as the first-choice of offloading treatment to promote healing of the ulcer. (Strong; High)	Adapted	**1a**	In a person with diabetes and a neuropathic plantar forefoot or midfoot ulcer, use a non-removable knee-high offloading device rather than a removable offloading device to promote healing of the ulcer (GRADE strength of recommendation: Strong; Quality of evidence: Moderate).
1b	When using a non-removable knee-high offloading device to heal a neuropathic plantar forefoot or midfoot ulcer in a person with diabetes, use either a total contact cast or non-removable knee-high walker, with the choice dependent on the resources available, technician skills, patient preferences and extent of foot deformity present. (Strong; Moderate)	Adapted	**1b**	When using a non-removable knee-high offloading device to heal a neuropathic plantar forefoot or midfoot ulcer in a person with diabetes, consider using either a total contact cast or nonremovable knee-high walker, with the choice dependent on the local resources and technical skills available, and person’s preferences and extent of foot deformity (Weak; Low).
2	In a person with diabetes and a neuropathic plantar forefoot or midfoot ulcer for whom a non-removable knee-high offloading device is contraindicated or not tolerated, consider using a removable knee-high offloading device with an appropriate foot-device interface as the second-choice of offloading treatment to promote healing of the ulcer. Additionally, encourage the patient to wear the device at all times. (Weak; Low)	Adapted	**2**	In a person with diabetes and a neuropathic plantar forefoot or midfoot ulcer, when non-removable knee-high offloading devices are contraindicated or not tolerated, consider using a removable knee-high offloading device (and explain the importance of using) during all weight-bearing activities rather than a removable ankle-high offloading device to reduce plantar pressure and promote healing of the ulcer (Weak; Low).
3	In a person with diabetes and a neuropathic plantar forefoot or midfoot ulcer for whom a knee-high offloading device is contraindicated or not tolerated, use a removable ankle-high offloading device as the third-choice of offloading treatment to promote healing of the ulcer. Additionally, encourage the patient to wear the device at all times. (Strong; Low)	Adapted	**3**	In a person with diabetes and a neuropathic plantar forefoot or midfoot ulcer, when knee-high offloading devices are contraindicated or not tolerated, use a removable ankle-high offloading device (and explain the importance of using) during all weight-bearing activities rather than medical grade footwear to promote healing of the ulcer (Strong; Very low)
4a	In a person with diabetes and a neuropathic plantar forefoot or midfoot ulcer, do not use, and instruct the patient not to use, conventional or standard therapeutic footwear as offloading treatment to promote healing of the ulcer, unless none of the above-mentioned offloading devices is available. (Strong; Moderate)	Adapted	**4**	In a person with diabetes and a neuropathic plantar forefoot or midfoot ulcer, when ankle-high offloading devices are contraindicated or not tolerated, use medical grade footwear rather than other footwear types or no footwear to reduce plantar pressure and promote healing of the ulcer (Strong; Low).
4b	In that case, consider using felted foam in combination with appropriately fitting conventional or standard therapeutic footwear as the fourth choice of offloading treatment to promote healing of the ulcer. (Weak; Low)	Adapted	**5**	In a person with diabetes and a neuropathic plantar forefoot or midfoot ulcer, consider using felted foam in combination with an offloading device or footwear rather than using the offloading device or footwear alone to further reduce plantar pressure and promote healing of the ulcer (Weak; Very Low).
5	In a person with diabetes and a neuropathic plantar metatarsal head ulcer, consider using Achilles tendon lengthening, metatarsal head resection(s), or joint arthroplasty to promote healing of the ulcer, if non-surgical offloading treatment fails. (Weak; Low)	Adapted	**6a**	If the best recommended offloading device option fails to heal a person with diabetes and a neuropathic plantar metatarsal head ulcer, consider using Achilles tendon lengthening or Gastrocnemius recession, metatarsal head resection(s), or joint arthroplasty to promote healing of the ulcer (Weak; Low).
6	In a person with diabetes and a neuropathic plantar or apex digital ulcer, consider using digital flexor tenotomy to promote healing of the ulcer, if non-surgical offloading treatment fails. (Weak; Low)	Adapted	**6b**	If the best recommended offloading device option fails to heal a person with diabetes and a neuropathic plantar or apical ulcer on a non-rigid toe, consider using digital flexor tenotomy to promote healing of the ulcer (Weak; Low).
7a	In a person with diabetes and a neuropathic plantar forefoot or midfoot ulcer with either mild infection or mild ischemia, consider using a non-removable knee-high offloading device to promote healing of the ulcer. (Weak; Low)	Adopted	**7a**	As stated in original the IWGDF Recommendation
7b	In a person with diabetes and a neuropathic plantar forefoot or midfoot ulcer with both mild infection and mild ischemia, or with either moderate infection or moderate ischaemia, consider using a removable knee-high offloading device to promote healing of the ulcer. (Weak; Low)	Adopted	**7b**	As stated in original the IWGDF Recommendation
7c	In a person with diabetes and a neuropathic plantar forefoot or midfoot ulcer with both moderate infection and moderate ischaemia, or with either severe infection or severe ischemia, primarily address the infection and/or ischemia, and consider using a removable offloading intervention based on the patient’s functioning, ambulatory status and activity level, to promote healing of the ulcer. (Weak; Low)	Adopted	**7c**	As stated in original the IWGDF Recommendation
8	In a person with diabetes and a neuropathic plantar heel ulcer, consider using a knee-high offloading device or other offloading intervention that effectively reduces plantar pressure on the heel and is tolerated by the patient, to promote healing of the ulcer. (Weak; Low)	Adopted	**8**	As stated in original the IWGDF Recommendation
9	In a person with diabetes and a non-plantar foot ulcer, use a removable ankle-high offloading device, footwear modifications, toe spacers, or orthoses, depending on the type and location of the foot ulcer, to promote healing of the ulcer. (Strong; Low)	Adapted	**9**	In a person with diabetes and a non-plantar foot ulcer, use a removable offloading device, medical grade footwear, felted foam, toe spacers or orthoses, depending on the type and location of the foot ulcer, rather than no offloading intervention to promote healing of the ulcer and to prevent further ulceration (Strong; Very Low).

Note: underlined wording indicates the specific adapted changes to the original IWGDF recommendation

We received 14 responses (nine individuals and five organisations) to the public consultation survey with collated responses displayed in Table 4. No respondents (0%) disagreed with the statements that: there was a need for a new offloading guideline, the methodology used for these guidelines was appropriate, the recommendations were clear, when applied the recommendations should produce more benefits than harms, and they would be comfortable if people with DFU received these recommendations. However, most respondents agreed that to implement the recommendations may require some reorganisation of services (77%), may be technically challenging (77%), may be too expensive (54%), but were likely acceptable to people living with DFD (77%). Overall, 12 of the 14 respondents (85%) (strongly) agreed that the guideline should be approved as the new Australian offloading guideline and none (0%) disagreed that the guideline would be supported by the majority of their colleagues and would encourage its use if approved. All de-identified comments received during public consultation and the panel’s responses were collated and are available on the Diabetes Feet Australia website.

**Table 4:** Summary public consultation survey responses (*n* = 14)

No.	Item	n	Strongly Agree	Agree	Neither Agree or Disagree	Disagree	Strongly Disagree
Background							
1	You are involved with the care of patients for whom this draft Australian offloading guideline is relevant.	14	11 (78.6%)	0	3 (21.4%)	0	0
2	There is a need for a new Australian offloading guideline in this population.	14	9 (64.35%	5 (35.7%)	0	0	0
3	The rationale for developing a new Australian offloading guideline on this topic is clear in this draft guideline.	14	9 (64.35%	5 (35.7%)	0	0	0
Methodology							
4	I agree with the overall methodology used to develop this draft Australian offloading guideline.	14	6 (42.9%)	6 (42.9%)	2 (14.3%)	0	0
5	The search strategy used to identify international guidelines on which this draft Australian offloading guideline was based is relevant and complete	14	5 (35.7%)	7 (50.0%)	2 (14.3%)	0	0
6	The methods used to determine the suitability of identified international source guidelines upon which this draft Australian offloading guideline were based were robust.	14	5 (35.7%)	7 (50.0%)	2 (14.3%)	0	0
7	I agree with the methods used within this draft Australian offloading guideline to interpret the available evidence on this topic.	14	5 (35.7%)	7 (50.0%)	2 (14.3%)	0	0
8	The methods used to decide which recommendations to adopt, adapt or exclude for the Australian context were objective and transparent.	14	5 (35.7%)	8 (57.1%)	1 (7.1%)	0	0
Recommendations							
9	The recommendations in this draft Australian offloading guideline are clear.	14	8 (57.1%)	4 (28.6%)	2 (14.3%)	0	0
10	I agree with the recommendations in this draft Australian offloading guideline as stated.	14	5 (35.7%)	6 (42.9%)	3 (21.4%)	0	0
11	The recommendations are suitable for people living with diabetes-related foot disease.	14	5 (35.7%)	6 (42.9%)	1 (7.1%)	1 (7.1%)	0
12	The recommendations are too rigid to apply for people living with diabetes-related foot disease.	14	2 (14.3%)	1 (7.1%)	3 (21.4%)	6 (42.9%)	2 (14.3%)
13	The recommendations reflect a more effective approach to improving patient outcomes than is current practice.	14	5 (35.7%)	3 (21.4%)	4 (28.6%)	2 (14.3%)	0
14	When applied, the recommendations should produce more benefits than harms for people living with diabetes-related foot disease.	14	7 (50%)	6 (42.9%)	1 (7.1%)	0	0
15	When applied, the recommendations should result in better use of resources than current practice allows.	14	6 (42.9%)	4 (28.6%)	3 (21.4%)	1 (7.1%)	0
16	I would feel comfortable if people living with diabetes-related foot disease received the care recommended in this draft Australian offloading guideline.	14	8 (57.1%)	4 (28.6%)	2 (14.3%)	0	0
Implementation of recommendations							
17	To apply the draft Australian offloading guideline may require reorganisation of services/care.	13	5 (38.5%)	5 (38.5%)	2 (15.4%)	1 (7.7%)	0
18	To apply the draft Australian offloading guideline may be technically challenging.	13	4 (30.8%)	6 (46.2%)	2 (15.4%)	1 (7.7%)	0
19	The draft Australian offloading guideline may be too expensive to apply.	13	4 (30.8%)	2 (23.1%)	3 (23.1%)	3 (23.1%)	1 (7.7%)
20	The draft Australian offloading guideline presents options that will likely be acceptable to people living with diabetes-related foot disease.	13	3 (23.1%)	7 (53.9%)	1 (7.7%)	2 (15.4%)	0
Final thoughts							
21	This draft guideline should be approved as the new Australian offloading guideline.	13	6 (46.2%)	5 (38.5%)	1 (7.7%)	1 (7.7%)	0
22	This draft Australian offloading guideline would be supported by the majority of my colleagues.	13	5 (38.5%)	7 (53.9%)	1 (7.7%)	0	0
23	If this draft guideline was to be approved as the new Australian offloading guideline, I would use or encourage their use in practice.	13	8 (61.5%)	4 (30.8%)	1 (7.7%)	0	0

Based on the collated public consultation feedback, the guideline was revised, approved by the panel and the Australian DFD Guidelines development working group, and endorsed as the new *Australian guideline on offloading treatment for foot ulcers* by ten national peak bodies including the Australian Podiatry Association, Wounds Australia, Australian and New Zealand Society for Vascular Surgery, Australasian Society for Infectious Diseases, Australian Orthotic Prosthetic Association, Pedorthic Association of Australia, Australian Advanced Practicing Podiatrists - High Risk Foot Group, Australian Aboriginal and Torres Strait Islander Diabetes-related Foot Complications Program, Australian Diabetes Society and Diabetes Feet Australia.

**Fig. 1:**
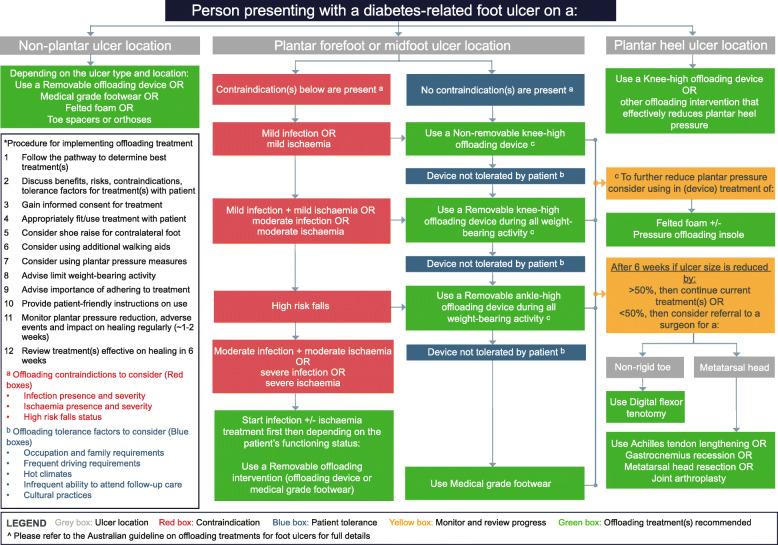
Australian evidence-based clinical pathway on offloading treatment for people with diabetes-related foot ulcers (DFU)*^

The 13 recommendations (and reasons) for this Australian evidence-based guideline on offloading treatment for people with DFU are grouped into five Sections (A-E) below. Sections A-D cover the different offloading treatments to use (or not use) for those with a plantar forefoot or midfoot DFU: A. Offloading devices, B. Footwear, C. Other (non-surgical) offloading techniques, D. Surgical offloading interventions. Whereas the final section covers the offloading treatments to use for those with E. Other DFU types and locations, such as those with infected or non-plantar DFU. Each section contains the following sub-sections: the question(s) posed; the new Australian recommendation(s); the decision (and rationale) to adopt, adapt or exclude the original IWGDF recommendation(s); the justifications supporting the new recommendation(s); and the implementation considerations (including descriptions, contraindications, procedures, monitoring and for geographically remote and Aboriginal and Torres Strait Islander people) for the recommendation(s). A summary of implementation considerations can be found in Table 5, and detailed justifications and implementation considerations can be found in the eTables (A1, A2, A3, A4, A5, A6, A7, A8 and A9, B1, B2, B3, B4, B5, B6, B7, B8, B9, B10, B11, B12 and B13) in the Supplementary Material. Finally, all recommendations are incorporated in the Australian evidence-based clinical pathway on offloading treatment for people with DFU in Fig. 1.

**Table 5:** Summary implementation considerations for the Australian evidence-based offloading treatment guidelines

No	Treatment or scenario	Contraindications	Procedures	Monitoring	Considerations in the Australian context	Additional information
**1a**	Irremovable knee-high offloading devices.	For those with high falls risk [32], moderate-to-severe infection and/or moderate-to-severe ischaemia [22, 33, 34] consider Recommendations 3, 7B and 7C, respectively. Consider personal circumstances [22], such as because of occupation, family care requirements, frequent driving, hot climates, social impacts or infrequent ability to attend follow-up care. For these people we suggest also considering Recommendation 2.	We strongly advise that the benefits, risks and contraindications are always carefully explained and people with DFU have an opportunity to discuss their personal circumstances to gain full informed consent. Offloading treatment is always performed in conjunction with a good standard of DFU care that includes DFU measurement, appropriate debridement, wound dressings, antimicrobial treatment if infected, revascularisation considerations if ischaemic [9, 35]. We refer the reader to the specific recommendations for such care in the relevant accompanying guidelines (REFS).	We suggest all people have their offloading regularly reviewed within ≤1 week of initial offloading device use and ~ 1–2 weekly thereafter - to monitor DFU healing, adverse events and plantar pressure where available.	Geographically remote people Aboriginal and Torres Strait Islander people.	See eTable B1 for further detailed information
**1b**	Total contact casts (TCC) and instant total contact cats (iTCC)	The same contraindications as in Recommendation 1A also apply for this recommendation. Additionally, large foot deformity is likely a contraindication for iTCCs	The same monitoring considerations as outlined in Recommendations 1A apply. Capture as data items/options to monitor the organisations use of either TCC or iTCC in the Australian context for audit and quality review and reporting purposes.			See eTable B2 for further detailed information
**2**	Removable knee-high offloading devices	The same contraindications as in Recommendation 1A.	The same procedures as in Recommendation 1A apply. Additionally, we agree with IWGDF that persons should be strongly advised to wear the device consistently.	Determine if the device is still optimally reducing plantar pressure and if the person is adhering to wearing the device as much as possible.		See eTable B3 for further detailed information.
**3**	Removable ankle-high offloading devices	People at high risk of mid-foot fractures if using half-shoe devices and people with very large foot deformity(s). Refer to Recommendation 4.	The same procedure considerations as in Recommendation 2. it is likely that higher ankle-high devices and those with rocker-soles may offer more plantar pressure reduction			See eTable B4 for further detailed information.
**4**	Medical grade footwear	People with a large foot deformity(s) that cannot be safely accommodated in prefabricated medical grade footwear.	Similar procedure considerations as outlined in Recommendations 1–3.	The same monitoring considerations as outlined in Recommendations 1–3.	See Recommendations 1–3. Often medical grade footwear is more difficult to source in geographically remote settings than removable offloading devices. Consider whether culturally appropriate.	See eTable B5 for further detailed information.
**5**	Felted foam (adhesive felt)	People with severe ischaemia, very fragile skin or heavily exudating ulcers are likely to be contraindicated to using felted foam that is adhered to the foot itself. Therefore, adhere the felted foam to the pressure offloading insole.	Similar procedure considerations as outlined in Recommendations 1–3. Ensure there is enough room in the device or footwear to safely accommodate the foot and felted foam, use a bevelled technique. Monitor for adverse events.	The same monitoring considerations as outlined in Recommendation 2 also apply.	Geographically remote people Aboriginal and Torres Strait Islander people.	See eTable B6 for further detailed information.
**6a**	Surgical offloading	A significant contraindication for these surgical procedures is moderate-to-severe ischaemia [22]. Relative contraindications include those with moderate-to-severe infection, moderate-to-severe oedema, cognitive impairment impairing capacity to provide informed consent, or conditions precluding anaesthesia. Lastly, we suggest people with normal (> 5 degrees of) ankle dorsiflexion are not likely to benefit from Achilles tendon lengthening or Gastrocnemius Recession procedures, and metatarsal head resections should be the surgical procedure considered instead. People with a rigid toe deformity are unlikely to benefit from Recommendation 6b.		The same monitoring considerations as outlined in Recommendations 1A also apply to this recommendation.		See eTable B7 for further detailed information.
**6b**			We strongly agreed with IWGDF that these surgical offloading procedures should only be considered if the person has failed to heal following 4–6 weeks of a good standard of DFU care			See eTable B8 for further detailed information.
**7a**	DFU complicated by infection or ischaemia	NA. The infection or ischaemia treatment plan should be instigated first. Please refer to Australian Guidelines on Infection and PAD [33, 34, 36].	See Recommendation 1	The same monitoring considerations as outlined in Recommendations 1–3 apply.		See eTable B9, 10 and B11 for further detailed information
**7b**			See Recommendation 2			
**7c**			See Recommendation 3			
**8**	Plantar heel DFU	The same contraindications as outlined in Recommendations 1–2	If considering ankle-high devices we highlight that such a device needs to demonstrate it can reduce more plantar pressure at the ulcer site than knee-high devices	The same monitoring considerations as outlined in Recommendations 1–2. Additionally, collect site of the ulcer as routine characteristics.		See eTable B12 for further detailed information
**9**	Non-plantar DFU	The same contraindications in Recommendations 2–5 apply.	Given there is a substantial lack of evidence, various removable non-surgical offloading modalities can be considered.	The same monitoring considerations in Recommendations 2–5 & 8 apply.		See eTable B13 for further detailed information.

## Recommendations

### A. Offloading devices

#### Q1 In people with a plantar DFU, are non-removable offloading devices compared to removable offloading devices effective to heal the DFU?

##### Australian recommendation 1A

In a person with diabetes and a neuropathic plantar forefoot or midfoot ulcer, use a non-removable knee-high offloading device rather than a removable offloading device to promote healing of the ulcer (GRADE strength of recommendation: Strong; Quality of evidence: Moderate).

##### Decision: Adapted (from the original IWGDF Recommendation)

*Rationale*: The panel decided to adapt the original IWGDF recommendation, based on differing judgements to the IWGDF for the quality of evidence rating and the need to include a comparison control treatment (Table 2). Therefore, we downgraded the quality of evidence from “high” to “moderate”, added “rather than a removable offloading device” as the control treatment, and removed the phrase “appropriate foot-device interface” as we considered this to have only limited indirect evidence to be included in this recommendation, and a term not used in Australia, and thus unnecessary (Table 3). For detailed justification see eTable A1 in the Supplementary Material.

##### Implementation considerations

For effective implementation we suggest the following considerations:

*Description*: We agreed with the IWGDF definition that non-removable knee-high offloading device are offloading devices that extend up the leg to just below the knee and cannot be readily removed by the patient, including total contact casts (TCCs) and non-removable walkers (often termed “instant TCCs”) [22].

*Contraindications*: We also agreed that contraindications for these devices include high falls risk [32], moderate-to-severe infection and/or moderate-to-severe ischaemia [22, 33, 34]. For people with these contraindications we instead suggest using Recommendations 3, 7B and 7C, respectively. We also agreed that there are people who due to a range of personal circumstances may not tolerate, or wish to wear, these devices following informed consent [22], such as because of occupation, family care requirements, frequent driving, hot climates, social impacts or infrequent ability to attend follow-up care. For these people we suggest also considering Recommendation 2.

*Procedures*: Before using any offloading device we strongly advise that the benefits, risks and contraindications are always carefully explained, and people with DFU have an opportunity to discuss and consider their personal circumstances, in order to first gain their full informed consent. This is particularly important in patients with neuropathy with loss of protective sensation and thus difficulty in sensing any benefits (e.g. healing) or risks (e.g. adverse events) to their feet when using offloading devices.

Following informed consent we strongly suggest health professionals always consider the following: appropriate fitting of the device, the pressure offloading material within the device (termed “appropriate foot-device interface” in the IWGDF guideline or “orthoses” in other guidelines, but hereto referred to as a “pressure offloading insole”), a shoe raise for the contralateral side to reduce any limb length difference, advice to limit weight-bearing activity and simple patient-friendly written instructions on safe offloading device use and when and how to seek advice [22, 37, 38]. Additionally, health professionals should consider the use of validated (i.e. proven accurate) in-shoe plantar pressure measurements where available and feasible and the use of any additional walking aids, such as walking frames, to support people to safely optimise plantar pressure reduction [22, 37, 38]. Finally, in terms of which type of non-removable knee-high offloading device to choose we refer the reader to Recommendation 1B below.

Offloading treatment is always recommended as part of a good standard of DFU care that includes best practice recommendations for DFU classification, local wound debridement, wound dressings, antibiotics (if infected), revascularisaton (if ischaemic), and patient-centred education [9, 35]. We refer the reader to the specific recommendations for such care in the relevant accompanying Australian guidelines for DFD [33, 34, 39–41].

*Monitoring*: We agreed with the IWGDF that offloading treatment is arguably the most important intervention for healing neuropathic plantar DFU [22]. Thus, we suggest all people have their offloading treatment regularly reviewed within ≤1 week of initial dispense of the offloading device, and ~ 1–2 weekly thereafter, to monitor plantar pressure reduction, adverse events and DFU healing. We strongly suggest 4–6 weeks after initial offloading device use, that the person’s DFU size and classification is carefully reviewed against the baseline DFU size at the time of initial offloading device dispense to determine if the DFU has reduced in size by > 50% in that time. A > 50% reduction suggests treatment is effective and can be continued, whereas a < 50% reduction in size should prompt formal review of the offloading treatment and wider DFU management plan [9, 35]. For offloading this should include reviewing whether the person is adherent to using the offloading device, limiting their weight-bearing activity, and whether the device is providing optimal plantar pressure reduction at the DFU site [10, 22]. If at this review, it is thought that other offloading treatments may improve these factors, we then refer the reader to the subsequent recommendations in this guideline (see Recommendations 2–6).

We suggest organisations routinely managing DFU should include at least one offloading data item/field in their organisation’s DFU database monitoring system to enable at least one annual offloading treatment key clinical performance indicator [22, 42] to objectively monitor the proportion of eligible patients (not contraindicated) with plantar DFU that are prescribed non-removable knee-high offloading devices [22, 42] or alternative devices, in-line with local patient preferences, resource utilisation and DFU healing rates [42, 43]. We refer the reader to existing national and state-based High Risk Foot Service database monitoring systems and datasets that typically include such offloading treatment items and indicators and are typically available to most Australian organisations to utilise [42–44].

*Geographically remote people*: In addition to the above considerations, the panel suggests for people from geographically remote locations that the potential infrequent access to follow-up care, hot climates and dusty environments that may result in a higher likelihood of adverse events should also be considered. In these circumstances, the balance of effects may favour Recommendation 2 compared to Recommendation 1.

*Aboriginal and Torres Strait Islander people*: In addition to all above considerations, the panel suggests for Aboriginal and Torres Strait Islander people that further personal circumstances are also carefully considered as part of the informed consent process, including the person’s need to participate in any traditional cultural practices where footwear may need to be removed. Further, we strongly suggest that all above considerations are discussed with the person in collaboration with their family, caregivers and support networks and a local Aboriginal and Torres Strait Islander Health Care Worker(s) where available, to optimise the person’s understanding of the benefits, risks, personal circumstances and requirements of these devices, such as length of time the device would likely need to be worn to heal the DFU. We also suggest health professionals consider facilitating culturally appropriate follow-up care for Aboriginal and Torres Strait Islander people where or if possible, such as via liaising with local Aboriginal and Torres Strait Islander Health Care Worker(s), local Aboriginal Community Controlled Health Services, using Aboriginal Medical Benefit Scheme entitlements and developing culturally-appropriate resources [45]. Lastly, we suggest health professionals consider the aesthetic appearance of such devices for Aboriginal and Torres Strait Islander people and whether the user would like their culture represented in the form of artwork or insignia to further personalise the device.

For more detailed considerations see eTable B1 in Supplementary Material.

#### Q2 In people with a plantar DFU, are total contact casts (TCC) compared to other non-removable knee-high offloading devices effective to heal the DFU?

##### Recommendation 1B

When using a non-removable knee-high offloading device to heal a neuropathic plantar forefoot or midfoot ulcer in a person with diabetes, consider using either a total contact cast or non-removable knee-high walker, with the choice dependent on the local resources and technical skills available, and the person’s preference and extent of foot deformity (Weak; Low).

##### Decision*:* Adapted

*Rationale*: The panel decided to adapt this recommendation as we had differing judgements for the quality of evidence rating (Table 2). Therefore, we downgraded the quality of evidence from “moderate” to “low”, plus, we also downgraded the strength of recommendation from “strong” to “weak” to align with GRADE criteria for strength of recommendation where the recommendation is not favouring either the intervention or control [29, 30], as in this case. Further, we made minor modifications to the “choice dependent” phrasing to group the local organisational and patient factors more intuitively (Table 3). For detailed justifications see eTable A2 in Supplementary Material.

##### Implementation considerations

For effective implementation we suggest the following considerations:

*Description*: We agreed with IWGDF that total contact casts (TCCs) are custom-made, knee-high, non-removable casts that can be applied using several different methods and materials [22]; whereas non-removable walkers are prefabricated, knee-high devices such as CAM walkers, moonboots or air cast walkers, that are made irremovable by wrapping a layer of plaster of paris, fibreglass, cohesive bandage or tie wrap around the device [22].

*Contraindications*: The same contraindications in Recommendation 1A apply for this recommendation. Additionally, we agreed with IWGDF that a further contraindication for non-removable walkers are a large foot deformity(s) that cannot be safely accommodated in a prefabricated walker and may cause further ulcers, such as a very wide foot, plantigrade foot, a large Charcot foot, or extensive bunion [22]. For patients where their foot deformity cannot be accommodated in a prefabricated walker, we strongly suggest instead using a TCC [22].

*Procedures*: The same procedures as in Recommendation 1A apply. Additionally, we agree with IWGDF that the choice between a TCC or non-removable walker should be guided by the local organisation’s available resources and technical skills, and the person’s foot deformity status and preference [22]. As mentioned for those with a large foot deformity(s) a TCC is typically indicated [22]. Whereas, non-removable walkers may be preferred in those persons without large foot deformities, or in organisations with less resources, technical skills and time to apply, as they have been found to be equally effective, lighter in weight, quicker and easier to apply, and more cost-effective than TCCs [15, 22, 46]. Thus, the panel, strongly suggests that organisations routinely managing DFU should offer, or be able to directly refer for, both types of non-removable knee-high offloading devices to cater for the above situations. Finally, we agree with IWGDF that there is no standard method for manufacturing a TCC or non-removable knee-high walker [22], and instead refer the reader to the papers cited on manufacture to choose a method based on the above considerations and local discretion [47–49], plus, we suggest to consider using Recommendation 5 (i.e. felted foam in combination with the offloading device) for additional plantar pressure reduction if needed.

*Monitoring*: The same monitoring considerations as outlined in Recommendations 1A apply. Additionally, we suggest that the two types of non-removable offloading device types are included as data items to capture and monitor the organisation’s use and impact on DFU healing of these device types in organisational database monitoring systems.

*Geographically remote people*: In addition to the above considerations and those for geographically remote people in Recommendation 1A, we suggest if choosing a non-removable walker in a person with infrequent access to follow-up care, that health professionals consider using a cohesive bandage (e.g. Coban™) wrap to make non-removable. Such a wrap is potentially “removable” by people using scissors if needed in an emergency, such as for acute onset of moderate-to-severe swelling of the foot or leg from infection or oedema. Evidence of removal of the wrap may also serve as a surrogate indicator to the health professional of device removal and lower adherence to use.

*Aboriginal and Torres Strait Islander people*: In addition to above, the same considerations for Aboriginal and Torres Strait Islander people outlined in Recommendation 1A apply.

For more detailed considerations see eTable B2 in Supplementary Material.

#### Q3 In people with a plantar DFU, are removable knee-high offloading devices compared to other removable offloading devices effective to heal the DFU?

##### Recommendation 2

In a person with diabetes and a neuropathic plantar forefoot or midfoot ulcer, when non-removable knee-high offloading devices are contraindicated or not tolerated, consider using a removable knee-high offloading device (and explain the importance of using) during all weight-bearing activities rather than a removable ankle-high offloading device to reduce plantar pressure and promote healing of the ulcer (Weak; Low).

##### Decision*:* Adapted

*Rationale*: The panel decided to adapt this recommendation as we had differing judgements for the value of outcomes rating, the need to emphasise the importance of using the device at all times and the control treatment (Table 2). Therefore, we added “(and explain the importance of using) during all weight-bearing activities” as we considered this a critical part of the intervention, and “rather than a removable ankle-high offloading device” as the control treatment. The panel also noted that the primary superiority of the intervention was on “reducing plantar pressure” rather than ulcer healing and hence added in this surrogate outcome. We also removed “appropriate foot-device interface” and “second choice” as we considered both unnecessary given that this may be the “first choice” in some person’s circumstances (Table 3). For detailed justifications see eTable A3 in Supplementary Material.

##### Implementation considerations

For effective implementation we suggest the following considerations:

*Description*: We agreed with IWGDF that removable knee-high offloading devices are offloading devices that extend up the leg to just below the knee and can be readily removed by the patient, including prefabricated, knee-high, removable cast walkers, such as CAM walkers, moonboots or air cast walkers, or custom-made bi-valved knee-high TCCs [22].

*Contraindications*: We agreed with IWGDF that contraindications for these devices include high falls risk [32], severe infection and/or severe ischaemia [22, 33, 34]. For persons with these contraindications we instead refer to Recommendations 3 and 7C, respectively.

*Procedures*: The same procedures as in Recommendation 1A apply and we also agree with IWGDF that health professionals should explain the importance of wearing the device consistently. Such an explanation should highlight that wearing such a device for 100% of the person’s weight-bearing activity should provide similar plantar tissue stress reduction, and in turn healing effectiveness, to if using a gold standard non-removable knee-high device [10, 15, 22]. However, any non-adherence compromises or negates the effectiveness of the device and will likely lengthen the healing time. Lastly, we suggest to consider using Recommendation 5 (i.e. felted foam in combination with the offloading device) for further plantar pressure reduction and to consider the persons’ capacity to apply and adhere to using removable knee-high offloading devices.

*Monitoring*: The same monitoring considerations as outlined in Recommendations 1A also apply. In addition, we emphasise the need to review the specific removable knee-high device over time to determine if the device is still optimally reducing plantar pressure and if the person is adhering to wearing the device as much as possible. If either is significantly impacted, we suggest considering using another knee-high offloading device, or potentially an ankle-high device as there is low-quality evidence showing that people may be more adherent to an ankle-high device [15]. We also suggest that different removable offloading device types are monitored as data items in organisational DFU database monitoring systems [42, 43].

*Geographically remote people*: In addition to the above, the same considerations for geographically remote people outlined in Recommendation 1A apply.

*Aboriginal and Torres Strait Islander people*: In addition to above, the same considerations for Aboriginal and Torres Strait Islander people outlined in Recommendation 1A apply.

For more detailed considerations see eTable B3 in Supplementary Material.

##### Recommendation 3

In a person with diabetes and a neuropathic plantar forefoot or midfoot ulcer, when knee-high offloading devices are contraindicated or not tolerated, use a removable ankle-high offloading device (and explain the importance of using) during all weight-bearing activities rather than medical grade footwear to promote healing of the ulcer (Strong; Very low).

##### Decision: Adapted

*Rationale*: The panel decided to adapt this recommendation as we had differing judgements for desirable effects and quality of evidence ratings, and the need to emphasise the importance of using the device and the control treatment (Table 2). Therefore, we downgraded the quality of evidence from “low” to “very low”, added “(and explain the importance of using) during all weight-bearing times” as we considered critical to the intervention, and “rather than medical grade footwear” as the control treatment. We also removed “appropriate foot-device interface” and “third choice” as we considered unnecessary given this may be the “first choice” in some person’s circumstances (Table 3). For detailed justifications see eTable A4 in Supplementary Material.

##### Implementation considerations

For effective implementation we suggest the following considerations:

*Description*: We agreed with IWGDF that removable ankle-high offloading devices are offloading devices that extend up the leg no higher than just above the ankle and can be readily removed by the patient [22]. We also agree that this definition incorporates a broad range of devices, including ankle-high walkers, forefoot offloading shoes, half shoes, cast shoes, healing sandals, postoperative healing shoes, and custom-made temporary shoes [22].

*Contraindications*: We agreed with IWGDF that a specific contraindications for removable ankle-high devices are the use of half shoe offloading devices as they have been reported to potentially increase the risk of midfoot fractures [22]. Otherwise a further potential contraindication is a very large foot deformity(s) that is unable to be accommodated by any ankle-high offloading device. For persons with these contraindications we instead refer to Recommendation 4.

*Procedures*: The same procedure considerations as in Recommendation 2 apply. Additionally, we suggest health professionals be aware that there is a broad range of ankle-high devices that may offer a broad range of plantar pressure reduction capabilities. However, it is likely that higher ankle-high devices and those with rocker-soles offer more plantar pressure reduction, such as ankle high walkers. Again, we also suggest considering using Recommendation 5 (i.e. felted foam in combination with the offloading device) to further reduce plantar pressure at the ulcer site. Lastly, we suggest medical grade footwear can be considered an option for this recommendation (see Recommendation 4), but only in circumstances where this footwear can be demonstrated to offer superior plantar pressure reduction at the person’s ulcer site compared to available ankle-high offloading device options.

*Monitoring*: The same monitoring considerations as outlined in Recommendation 2 also apply to this recommendation.

*Geographically remote people*: In addition to the above, the same considerations for geographically remote people outlined in Recommendation 2 apply.

*Aboriginal and Torres Strait Islander people*: In addition to above, the same considerations for Aboriginal and Torres Strait Islander people outlined in Recommendation 2 apply.

For more detailed considerations see eTable B4 in Supplementary Material.

### B. Footwear

#### Q4 In people with a plantar DFU, are conventional or standard therapeutic footwear compared to other (non-surgical) offloading interventions effective to heal the DFU?

##### Recommendation 4

In a person with diabetes and a neuropathic plantar forefoot or midfoot ulcer, when ankle-high offloading devices are contraindicated or not tolerated, use medical grade footwear rather than other footwear types or no footwear to promote healing of the ulcer (Strong; Low).

##### Decision: Adapted

*Rationale*: The panel decided to adapt this recommendation as we had differing judgements for value of outcomes, desirable effects, undesirable effects and quality of evidence ratings, the need to emphasise the control treatment and be a positive recommendation (Table 2). Therefore, we downgraded the quality of evidence from “moderate” to “low”, added “rather than other footwear types or no footwear” as the control treatments, and removed “do not use” to change the context from a negative to a positive recommendation as it would be “when ankle-high devices are contraindicated” or where no offloading devices were available. We also replaced “therapeutic footwear “with the Australian term “medical grade footwear” [37], and modified “unless none of the abovementioned offloading devices is available” to “when ankle-high offloading devices are contraindicated or not tolerated” to further emphasise when this recommendation is appropriate and align better with the wording of earlier recommendations (Table 3). For detailed justifications see eTable A5 in Supplementary Material.

##### Implementation considerations

For effective implementation we suggest the following considerations:

*Description*: We agreed with IWGDF that therapeutic footwear is a generic term for footwear that is specially designed to have a therapeutic effect on foot health [22]. The 2018 Australian diabetes footwear guideline’s term for such therapeutic footwear is “medical grade footwear” and incorporates both prefabricated or custom-made types [37]. Prefabricated medical grade footwear is typically only available from speciality footwear shops and provides special features designed to accommodate a broader range of foot types than standard off-the-shelf footwear, including extra depth, multiple width fittings, modified soles, fastenings and/or smooth internal linings features [37]. Custom-made medical grade footwear is typically uniquely manufactured for one person, by a trained footwear health professional, when the person cannot be safely accommodated in prefabricated medical grade footwear and is typically made to accommodate large foot deformity(s) and/or relieve pressure over at-risk sites on the plantar and dorsal surfaces of the foot [37].

*Contraindications*: We are unaware of any significant sub-groups who may be contraindicated to correctly fitted medical grade footwear [37]. However, a contraindication for prefabricated medical grade footwear are those with a large foot deformity(s) that cannot be safely accommodated in prefabricated medical grade footwear, such as a very wide foot, plantigrade foot, a large Charcot foot, or extensive bunion [22, 37]. We strongly suggest using custom-made medical grade footwear instead in these cases.

*Procedures*: Similar procedure considerations as outlined in Recommendations 1–3 also apply to medical grade footwear, including appropriate fitting, pressure offloading insoles (termed “appropriate foot-device interface” in the IWGDF guideline or “orthoses” in other guidelines, but hereto referred to as a “pressure offloading insole”), shoe raise for the contralateral shoe, advice to limit weight-bearing activity, written patient-centred follow-up care information and to see Recommendation 5 for additional felted foam supports that may be utilised to supplement offloading devices. Additionally, we agree with the Australian diabetes footwear guidelines that custom-made medical grade footwear requires an in-depth assessment by a trained footwear health professional (such as a pedorthist or orthotist/prosthetist) that typically includes multiple measurements, impressions or a mould, and a positive model of a person’s foot for manufacture [37]. We again highlight, that medical grade footwear is typically only recommended for treating those with DFU when offloading devices are contraindicated or where no other offloading devices are available, as the balance of effects strongly favours offloading devices rather than medical grade footwear due to the moderate additional desirable effects (for healing, plantar pressure reduction, activity reduction, costs and cost-effectiveness) and trivial undesirable effects (for adverse events and patient preference) to heal people with DFU [15, 22]. We consider the only exception to this is if the medical grade footwear is demonstrated to offer superior plantar pressure reductions at the person’s ulcer site than offloading device options using validated plantar pressure equipment measurements. Therefore, medical grade footwear should nearly always be considered a last, stop-gap offloading treatment to heal a person with DFU until offloading devices can be obtained. However, we do note that as recommended in the accompanying Australian guideline to prevent DFU there is moderate quality of supporting evidence for the use of medical grade footwear to prevent recurrence of DFU once healed [39]. Thus, we suggest that health professionals strongly consider arrangements to transition into medical grade footwear when healing is (nearly) achieved as per expert consensus guidelines [50] and refer the reader to the accompanying Australian guideline to prevent DFU [39]. Finally, while there is no literature to support their use as treatment to heal people with DFU, wheelchairs, knee scooters or electric scooters may be considered in these rare circumstances.

*Monitoring*: The same monitoring considerations as outlined in Recommendations 1–3 also apply to this recommendation. Additionally, we suggest that the use of medical grade footwear is perhaps captured and monitored in organisational monitoring systems to try and ensure that medical grade footwear to offload DFU is only used in those rare circumstances.

*Geographically remote people*: In addition to the above, similar considerations for geographically remote people outlined in Recommendations 1–3 apply. However, we do highlight that often medical grade footwear is more difficult to source in geographically remote settings than removable offloading devices, and thus offloading devices are likely a much practical option for people with DFU (See Recommendations 1–3).

*Aboriginal and Torres Strait Islander people*: In addition to above, similar considerations for Aboriginal and Torres Strait Islander people outlined in Recommendation 1–3 apply. Additionally, in situations where Aboriginal and Torres Strait Islander people are not in agreement to use offloading devices, or prefer a different approach, we suggest considering whether offloading devices or medical grade footwear are made more culturally appropriate for these circumstances. Only as a very last resort we suggest that health professionals consider the benefits and risk of using well-fitted off-the-shelf footwear rather than no footwear at all if they are the only options available. We refer the reader to the Australian diabetes footwear guidelines in these circumstances [37].

For more detailed considerations see eTable B5 in Supplementary Material.

### C. Other (non-surgical) offloading techniques

#### Q5 In people with a plantar DFU, are any other offloading techniques that are not device or footwear-related, effective to heal a DFU?

##### Recommendation 5

In a person with diabetes and a neuropathic plantar forefoot or midfoot ulcer, consider using felted foam in combination with an offloading device or footwear rather than using the offloading device or footwear alone to further reduce plantar pressure and promote healing of the ulcer (Weak; Very Low).

##### Decision: Adapted

*Rationale*: The panel decided to adapt this recommendation as we had differing judgements for quality of evidence ratings, and the need to clarify the intervention treatment and emphasise the control treatment (Table 2). Therefore, we downgraded the quality of evidence from “low” to “very low”, modified the intervention from “using felted foam in combination with appropriately fitting conventional or standard therapeutic footwear” to “using felted foam in combination with an offloading device or footwear”, and added “rather than using the offloading device or footwear alone” as the control treatments. We also replaced “as the fourth choice” as we now conditionally recommend felted foam as an adjunct offloading treatment. Felted foam should therefore be considered to be used in conjunction with other offloading devices or footwear where appropriate (Table 3). Finally, we note for the Autralian reader that studies on felted foam and felt only were considered and reported collectively under the category of “felted foam” by IWGDF, and thus felt can be considered as a type of felted foam for this recommendation.

For detailed justifications see eTable A6 in Supplementary Material.

##### Implementation considerations

For effective implementation we suggest the following considerations:

*Description*: Felted foam is a term used for another (non-surgical) offloading intervention, that is a made from either a combined felt and foam material, or from felt alone, that has different densities and an adherent backing that enables it to be cut, contoured and fixed to a surface, typically the pressure offloading insole of an existing offloading device or footwear, or the foot [22, 51]. The type of felted foam most commonly used in Australia is semi-compressed wool felt with an adhesive backing [19, 52–54].

*Contraindications*: We agreed with IWGDF that we are unaware of any significant sub-groups who may be contraindicated to correctly fitted felted foam [22]. However, we suggest those with severe ischaemia or heavily exudating ulcers are likely to be contraindicated to using felted foam [22]. We suggest if choosing to use felted foam to consider adhering the felted foam to the pressure offloading insole in the offloading device or footwear to avoid injury to fragile skin and that felted foam paddings with apertures not be used for large wounds >2cm^2^.

*Procedures*: Similar procedure considerations as outlined in Recommendations 1–4 also apply to felted foam. Additionally, we suggest the following considerations when using felted foam: ensure there is enough room in the device or footwear to safely accommodate the foot and felted foam and minimise the effect of transferring load to other areas of the foot from the contoured area of the felted foam (around the ulcer site) by bevelling the edge of the felted foam or using in combination with other cushioning material [52]. Also, it is important to monitor for adverse events (such as transfer lesions, maceration or infection) and replace the felted foam at least weekly as it has been found to lose > 30% of its plantar pressure effects within a week of application [52]. Otherwise, we refer the reader to this cited Australian paper on the application and effect of different felted foam on plantar pressure when used within offloading devices in people with DFU [52]. Finally, we agree with IWGDF that felted foam is a modality to augment the plantar pressure reduction effect of existing offloading devices or footwear and should not be considered as a standalone intervention [22].

*Monitoring*: The same monitoring considerations as outlined in Recommendation 2 also apply. In addition, we suggest that felted foam may be considered as a secondary offloading treatment data item captured and monitored in organisation monitoring systems.

*Geographically remote people*: No additional considerations to that outlined above apply, except that it is even more important in locations with hot, humid or dusty environments to monitor the DFU and surrounding foot integrity for adverse events.

*Aboriginal and Torres Strait Islander people*: No additional considerations to those outlined above apply.

For more detailed considerations see eTable B6 in Supplementary Material.

### D. Surgical offloading techniques

#### Q6 In people with a DFU, are surgical offloading techniques compared to non-surgical offloading interventions effective to heal the DFU?

##### Recommendation 6A

If the best recommended offloading device option fails to heal a person with diabetes and a neuropathic plantar metatarsal head ulcer, consider using Achilles tendon lengthening or Gastrocnemius recession, metatarsal head resection(s), or joint arthroplasty to promote healing of the ulcer (Weak; Low).

##### Decision: Adapted

*Rationale*: The panel decided to adapt this recommendation as we considered the available evidence for desirable and undesirable effects also supported Gastrocnemius Recession procedures being included alongside the three surgical offloading procedures in the original IWGDF recommendation (Table 2). Therefore, we added “or Gatrocnemius recession” and we also moved and modifed the phrase “if non-surgical offloading treatment fails” to the start of the recommendation to highlight this important caveat earlier in the recommendation (Table 3). The panel also defined “if the best recommended offloading device option fails to heal” as treatment failure when following a step down approach of using the best recommended offloading devices option that is not contraindicated and is tolerated by the person. The panel defines “fails to heal” as the DFU not reducing in size by > 50% of its baseline size after 4–6 weeks of receiving the best recommended offloading device in conjunction with other recommended good standard of DFU care (see procedure in Recommendation 1A for more details). For detailed justifications see eTable A7 in Supplementary Material.

##### Implementation considerations

For effective implementation we suggest the following considerations:

*Description*: We agreed with IWGDF that surgical offloading is an overarching term used to describe a surgical procedure undertaken with the intention of relieving mechanical stress from a specific region of the foot, and for this recommendation, is evidenced to include the specific procedures of Achilles tendon lengthening, Gastrocnemius Recession (with or without soleal fascial lengthening), metatarsal head resection, and joint arthroplasty [22].

*Contraindications*: We agreed with IWGDF that a significant contraindication for these surgical procedures is moderate-to-severe ischaemia [22]. Furthermore, we suggest other sub-groups of people are also likely to be contraindicated and include those with moderate-to-severe infection, moderate-to-severe oedema, cognitive impairment that impairs capacity to provide informed consent, or conditions precluding anaesthesia. Lastly, we suggest people with normal (> 5 degrees of) ankle dorsiflexion are not likely to benefit from Achilles tendon lengthening or Gastrocnemius Recession procedures, and metatarsal head resections should be the surgical procedure considered instead in these circumstances [55]. Otherwise as persons undergoing these procedures will be required to post-operatively use offloading devices, we refer the reader back to contraindications in Recommendations 1–4.

*Procedures*: We strongly agreed with IWGDF that these surgical offloading procedures should only be considered if the person has failed to heal following 4–6 weeks of a good standard of DFU care [9, 22, 35]. We suggest a good standard of DFU care includes best practice recommendations for DFU classification, local wound debridement, wound dressings, antibiotics (if infected), revascularisaton (if ischaemic), patient-centred education (see recommendations in the accompanying Australian DFD guidelines [33, 34, 39–41]) and the best available offloading device (see Recommendations 1–4) [9, 35]. We suggest failure to heal is defined as the DFU not reducing in size by > 50% after receiving 4–6 weeks of such a good standard of DFU care [9, 35].

If the patient has failed to heal, we again strongly advise that the benefits, risks, contraindications and personal circumstances are always carefully discussed first with person to gain their informed consent for any surgical offloading procedure (see general procedure considerations in Recommendation 1A). Following informed consent, we strongly suggest that best practice DFU and general health assessments are re-performed to ensure the patient is indicated and fit for surgery, and that any lower limb surgeon considering performing surgical offloading procedures is appropriately trained, suitably qualified, able to demonstrate competence in the specific procedure concerned and be registered with the appropriate regulatory body. Lastly, we suggest that post-operative management of the patient involves a multi-disciplinary team performing a good standard of DFU care that includes using the best available offloading device until the DFU is healed. Otherwise, we refer the reader to the same general procedure considerations outlined for those offloading devices in Recommendations 1–4.

*Monitoring*: The same monitoring considerations as outlined in Recommendations 1A also apply to this recommendation. In addition, we suggest that the surgical offloading procedures included in this recommendation are also captured and monitored in offloading indicators and organisational monitoring systems [42, 43]. Furthermore, we suggest that organisations could consider engaging their local health information managers to help obtain routinely collected hospital surgical procedure data from their local hospital datasets using Australian Classification of Health Interventions codes for these specific surgical procedures as another method of monitoring the appropriate access and use of these surgical procedures as well [56, 57].

*Geographically remote people*: In addition to the above, similar considerations for geographically remote people outlined in Recommendations 1–3 apply. Additionally, we suggest when discussing the above benefits, risks, contraindications and personal circumstances for these procedures with geographically remote people, that the likely need for people to travel to large metropolitan tertiary hospitals to receive these procedures and post-operative DFU care are also discussed as part of the informed consent processes.

*Aboriginal and Torres Strait Islander people*: In addition to all the above, similar considerations for Aboriginal and Torres Strait Islander people outlined in Recommendation 1–3 apply. We further highlight that all discussions with Aboriginal and Torres Strait Islander persons should be preferably performed in conjunction with family and Aboriginal and Torres Strait Islander Health Care Workers, and allow adequate time to discuss, understand and consider the benefits, risks, contraindications, personal circumstances and travel requirements of such procedures so as to enable the person and their family to make an informed decision. Otherwise, we are unaware of any guidelines for culturally appropriate discussions surrounding surgery with Aboriginal people, however, the panel feels the developments of such guidelines in surgical training would be most useful.

For more detailed considerations see eTable B7 in Supplementary Material.

##### Recommendation 6B

If the best recommended offloading device option fails to heal a person with diabetes and a neuropathic plantar or apical ulcer on a non-rigid toe, consider using digital flexor tenotomy to promote healing of the ulcer (Weak; Low).

##### Decision: Adapted

*Rationale*: The panel decided to adapt this recommendation as we considered the available evidence only supported performing this procedure in those with a digital flexion deformity (or non-rigid toe) and not in those with a rigid toe deformity (Table 2). Therefore, we added the phrase “on a non-rigid toe” to specify the population that this procedure is evidenced to benefit and again moved and modified the phrase “if non-surgical offloading treatment fails” to the start of the recommendation to highlight this important caveat (Table 3). Failure of “best recommended offloading device option” is defined in Recommendation 6A. For detailed justifications see eTable A8 in Supplementary Material.

##### Implementation considerations

For effective implementation we suggest the following considerations:

*Description*: We agreed with IWGDF that surgical offloading is an overarching term used to describe a surgical procedure undertaken with the intention of relieving mechanical stress from a specific region of the foot and for this recommendation is evidenced to include digital flexor tenotomy procedures only [22].

*Contraindications*: The same contraindications as in Recommendation 6A apply. In addition, we suggest people with a rigid toe deformity are unlikely to benefit from these procedures.

*Procedures*: The same general procedure considerations as in Recommendation 6A apply. Additionally, we suggest during the DFU assessment that the digital deformity is assessed to confirm it is a flexion deformity (or non-rigid toe).

*Monitoring*: The same monitoring considerations outlined in Recommendation 6A apply, plus adding digital flexor tenotomies as a surgical offloading item in monitoring systems.

*Geographically remote people*: The same considerations for geographically remote people outlined in Recommendation 6A apply.

*Aboriginal and Torres Strait Islander people*: The same considerations for Aboriginal and Torres Strait Islander people outlined in Recommendation 6A apply.

For more detailed considerations see eTable B8 in Supplementary Material.

### E. Other ulcer types and locations

#### Q7 In people with a plantar DFU complicated by infection or ischaemia, which offloading intervention is effective for healing the DFU?

##### Recommendation 7A

In a person with diabetes and a neuropathic plantar forefoot or midfoot ulcer with either mild infection or mild ischaemia, consider using a non-removable knee-high offloading device to promote healing of the ulcer (Weak; Low).

##### Decision: Adopted

*Rationale*: The panel decided to adopt this recommendation without change after screening. This was based on having no differences in judgements to the IWGDF and judging this recommendation to be acceptable and applicable in the Australian context (Table 1).

##### Recommendation 7B

In a person with diabetes and a neuropathic plantar forefoot or midfoot ulcer with both mild infection and mild ischaemia, or with either moderate infection or moderate ischaemia, consider using a removable knee-high offloading device to promote healing of the ulcer. (Weak; Low).

##### Decision: Adopted

*Rationale*: The panel decided to adopt this recommendation without change after screening, based on having no differences in judgements to the IWGDF and judging this recommendation to be acceptable and applicable in the Australian context (Table 1).

##### Recommendation 7C

In a person with diabetes and a neuropathic plantar forefoot or midfoot ulcer with both moderate infection and moderate ischaemia, or with either severe infection or severe ischaemia, primarily address the infection and/or ischaemia, and consider using a removable offloading intervention based on the patient’s functioning, ambulatory status and activity level, to promote healing of the ulcer (Weak; Low).

##### Decision: Adopted

*Rationale*: The panel decided to adopt this recommendation without change after screening, based on having no differences in judgements to the IWGDF and judging this recommendation to be acceptable and applicable in the Australian context (Table 1).

##### Implementation considerations for recommendations 7A-7C

For effective implementation we suggest the following considerations:

*Description*: We agreed with IWGDF that although the evidence is limited, offloading treatment for high plantar tissue stress is also vital to heal people with DFU complicated by infection or ischaemia [22]. However, we also agree that health professionals should be more cautious with their offloading treatment due to the risk of swelling (with moderate-to-severe infection) which could render the device too tight, and the need for frequent removal of the device to monitor the foot [22]. Also given the limb threatening nature of severe infection and the associated systemic illness, hospitalisation and bedrest is often indicated, and hence offloading considerations may exist only for transferring in these circumstances. We refer the reader to the accompanying Australian DFD guidelines for infection and PAD for definitions and management recommendations [33, 34, 36].

*Contraindications*: We note these recommendations are specifically recommending offloading treatments to use when patients with DFU have infection or ischaemia and are contraindicated to other offloading devices. However, regardless of those with different infection or ischaemia severity categories, we suggest those at high falls risk are contraindicated for knee-high offloading devices and suggest instead to use Recommendation 7C.

*Procedures*: We agreed with the IWGDF that an evidence-based DFU assessment should be initially undertaken to determine the infection or ischaemia severity category and in turn which of Recommendations 7A-7C to use [22]. We also agree in those assessed with limb-threatening severe infection, severe ischaemia or both moderate infection and moderate ischaemia, that their infection or ischaemia management plan should be of primary concern and instigated urgently. Thus, we refer the reader to the accompanying Australian DFD guidelines for infection and PAD for assessment and management recommendations [33, 34, 36]. However, we also agreed that in those with limb-threatening infection or ischaemia that these persons still importantly need offloading treatment to reduce plantar pressure and facilitate a healing DFU environment [22]. Thus, offloading treatment should ideally be provided on the same day as the infection or ischaemia management plan is instigated and not delayed waiting for resolution of infection or ischaemia. Otherwise we suggest the same considerations outlined in Recommendations 1 apply for Recommendation 7A, Recommendation 2 applies for Recommendation 7B and Recommendation 3 applies for Recommendation 7C.

*Monitoring*: The same monitoring considerations as outlined in Recommendations 1–3 apply. Additionally, we strongly suggest that the offloading treatment be reviewed at the same time as it is recommended to monitor the infection or PAD management and changed in accordance with any change in infection or ischaemia severity category. Lastly, we suggest that infection and PAD severity categories are also collected as part of the routine patient characteristics captured and monitored within organisational data monitoring systems to enable monitoring of patients with complications to ensure they are receiving recommended offloading treatment [42, 43].

*Geographically remote people*: The same above considerations for geographically remote people apply.

*Aboriginal and Torres Strait Islander people*: The same above considerations for Aboriginal and Torres Strait Islander apply.

For more detailed considerations see eTable B9, B10 and B11 in Supplementary Material.

#### Q8 In people with a plantar heel DFU, which offloading intervention is effective to heal the DFU?

##### Recommendation 8

In a person with diabetes and a neuropathic plantar heel ulcer, consider using a knee-high offloading device or other offloading intervention that effectively reduces plantar pressure on the heel and is tolerated by the patient, to promote healing of the ulcer. (Weak; Low).

##### Decision: Adopted

*Rationale*: The panel decided to adopt this recommendation without change after screening, based on having no differences in judgements to the IWGDF and judging this recommendation to be acceptable and applicable in the Australian context (Table 1).

##### Implementation considerations

For effective implementation we suggest the following considerations:

*Description*: We agreed with IWGDF that the definition of plantar heel ulcer is one on the plantar surface of the rearfoot (or hindfoot) which is composed of the talus, calcaneus and surrounding soft tissue [22, 58]. We also agreed that the prevalence of plantar heel DFU is lower than plantar forefoot DFU, the evidence to heal these plantar heel DFU is limited, but that these plantar heel DFU are often much more challenging to offload and pose a greater risk of amputation of the lower leg [22]. Thus, offloading treatment for excessive plantar pressure is arguably even more vital to heal people with these plantar heel DFU [22]. Otherwise we refer the reader to the descriptions of non-removable knee-high offloading devices in Recommendations 1 and removable knee-high offloading devices in Recommendation 2. Finally, we suggest that the ankle-high offloading devices outlined in Recommendations 3 may be used for plantar heel DFU, but only if they can demonstrate a superior plantar pressure reduction at the ulcer site than knee-high offloading devices.

*Contraindications*: The same contraindications as outlined in Recommendations 1–2 also apply, depending on the knee-high offloading device chosen.

*Procedures*: The same general procedures as outlined in Recommendation 1–2 apply, depending on the knee-high offloading device chosen. Additionally, if considering ankle-high devices we highlight that such a device needs to demonstrate it can reduce more plantar pressure at the heel DFU site than knee-high devices, using validated plantar pressure measuring equipment, to be chosen. Lastly, we also suggest that complete offloading of heel DFUs may be considered for severe DFU which fail to heal with knee-high offloading devices. While there is no literature to support their use as treatment to heal people with DFU, wheelchairs, knee scooters or electric scooters may be considered in these circumstances.

*Monitoring*: The same monitoring considerations as outlined in Recommendations 1–2 apply. Additionally, we suggest that different DFU locations (such as forefoot, midfoot, rearfoot and plantar or dorsal) are also collected as part of routine patient characteristics within organisational data monitoring systems to enable organisations to monitor if their patients are receiving the recommended offloading intervention for their ulcer location [42, 43].

*Geographically remote people*: The same considerations for geographically remote people in Recommendations 1–2 apply, depending on the knee-high offloading device chosen.

*Aboriginal and Torres Strait Islander people*: The same considerations for Aboriginal and Torres Strait Islander people in Recommendations 1–2 apply, depending on the knee-high offloading device chosen.

For more detailed considerations see eTable B12 in Supplementary Material.

#### Q9 In people with a non-plantar DFU, which offloading intervention is effective to heal the DFU?

##### Recommendation 9

In a person with diabetes and a non-plantar foot ulcer, use a removable offloading device, medical grade footwear, felted foam, toe spacers or orthoses, depending on the type and location of the foot ulcer, rather than no offloading intervention to promote healing of the ulcer and to prevent further ulceration (Strong; Very Low).

##### Decision: Adapted

*Rationale*: The panel decided to adapt this recommendation as we had differing judgements for desirable effects, undesirable effects and quality of evidence ratings, the need to also include other intervention options, the control treatment and to prevent another DFU (Table 2). Therefore, we downgraded the quality of evidence from “low” to “very low”, added any “removable offloading device” and “felted foam” as other intervention options, “rather than no offloading intervention” as the comparator, and “to prevent further ulceration” as another outcome of value. We again also replaced “footwear modifications “with the Australian term “medical grade footwear” that covers this definition (Table 3). For detailed justifications see eTable A9 in Supplementary Material.

##### Implementation considerations

For effective implementation we suggest the following considerations:

*Description*: We agreed with IWGDF that the definition of non-plantar DFU is for a DFU that is on a surface of the foot other than the plantar (weight-bearing) surface, including dorsal or interdigital surfaces of the foot [22, 58]. We also agreed that evidence suggests that non-plantar DFU are similar in prevalence to plantar DFU, however, the evidence to offload non-plantar DFU is nearly non-existent even though the expert opinion is that offloading (or protecting from) pressure from these non-plantar DFU is equally important for healing [22]. Otherwise we refer the reader to the descriptions of the various removable non-surgical offloading interventions, including removable offloading devices in Recommendations 2–3, medical grade footwear in Recommendation 4 and felted foam in Recommendation 5. Lastly, we agreed with IWGDF that toe spacers or orthoses are in-shoe orthoses designed to achieve some alteration in function of the toe and are typically customised from material such as silicon, rubber or foam [22].

*Contraindications*: The same contraindications in Recommendations 2–5 apply, depending on the specific removable non-surgical offloading intervention chosen.

*Procedures*: The panel agreed with IWGDF that given there is a substantial lack of evidence to guide offloading treatment for non-plantar DFUs [15], and until new evidence becomes available, that various removable non-surgical offloading modalities can be considered depending on the location of the nonplantar ulcer [22]. Otherwise the same procedures in Recommendation 2–5 apply, depending on the removable non-surgical offloading intervention chosen.

*Monitoring*: The same monitoring considerations in Recommendations 2–5 & 8 apply.

*Geographically remote people*: The same considerations for geographically remote people in Recommendations 2–5 apply, depending on the removable non-surgical offloading intervention chosen.

*Aboriginal and Torres Strait Islander people*: The same considerations for Aboriginal and Torres Strait Islander people in Recommendations 2–5 apply, depending on the removable non-surgical offloading intervention chosen.

For more detailed considerations see eTable B13 in Supplementary Material.

## Discussion

### Key findings and recommendations

We developed an Australian evidence-based guideline on offloading treatment for people with DFU by systematically adapting high-quality international guidelines to the Australian context. In Australia, we recommend a step-down offloading treatment approach for people with plantar DFU depending on their contraindications and tolerance. We strongly recommend non-removable knee-high offloading devices as first option unless contraindicated or not tolerated, then consider removable knee-high offloading devices second, removable ankle-high offloading devices third and medical grade footwear as last option. We also recommend considering using felted foam (or other pressure offloading insole) in combination with the chosen offloading device or footwear to further reduce plantar pressure. For people with non-plantar DFU we recommend using a removable offloading device, felted foam, toe spacers or orthoses, or medical grade footwear depending on the type and location of the foot ulcer. If offloading device options fail to heal a person with plantar DFU, depending on the location, we recommend considering various surgical offloading procedures. This new guideline, endorsed by ten key national peak bodies, should serve as the new national multi-disciplinary evidence-based offloading treatment guideline and the best practice standard of offloading care for people with DFU in Australia.

### Differences to previous guideline

There are now 13 offloading treatment recommendations in this new 2021 guideline compared with two offloading treatment recommendations in the previous 2011 guideline, i.e.: i) gold standard “use of a total contact cast or other device rendered irremovable”, and ii) where these irremovable devices could not be used then “other removable offloading devices may be considered” [16]. The increased number of 2021 guideline recommendations are at least in part due to the substantial new offloading evidence published since the last guideline, including at least 11 RCTs and six meta-analyses [15]. In this new 2021 guideline, non-removable knee-high offloading devices remain the gold standard offloading treatment (Recommendations 1). However, the big difference to the previous guideline is the detailed recommendations for circumstances when gold standard non-removable devices are contraindicated or unable to be tolerated. In these situations, we recommend considering removable knee-high or ankle-high offloading devices (Recommendations 2–3), footwear as a last resort (Recommendation 4) and considering felted foam (or pressure offloading insoles) to further reduce plantar pressure in all these devices (Recommendation 5). Furthermore, we recommend surgical offloading procedure options for when the best recommended offloading device treatment fails to heal a DFU (Recommendations 6), and various offloading devices or footwear options in people with DFUs complicated by infection, ischaemic or on a different foot location (Recommendations 7–9). Overall, this new guideline provides specific evidence-based offloading treatment options for nearly all circumstances for people with DFU in Australia.

### Implementation considerations

To try and optimise the uptake of these new recommendations into national clinical practice we provided a comprehensive range of implementation considerations for health professionals. These included facilitating patients to make an informed decision on which offloading treatment is best for their circumstances and other considerations when prescribing offloading treatments, such as including pressure offloading insoles and contralateral shoe raises [15, 22, 38]. We also provided considerations on when and how to monitor the efficacy of offloading treatments for individual patients [22, 38] and for organisations [42, 43]. Lastly, we distilled all recommendations into a one-page user-friendly clinical pathway to try and maximise uptake and implementation of these recommendations and considerations by busy Australian multi-disciplinary clinicians (Fig. 1).

In addition, we provided further specific implementation considerations for when treating people residing in geographically remote areas and Aboriginal and Torres Strait Islander peoples, such as the impact of limited or infrequent access to DFU care, hot climates, dusty environments and cultural practices. We emphasise that health professionals always consider it important to carefully explain and discuss with Aboriginal and Torres Strait Islander people the benefits and risks of the recommendations in the context of their personal and cultural circumstances. Ideally this should be performed in collaboration with family, caregivers, support networks and local Aboriginal and Torres Strait Islander health care workers to optimise understanding. Further, we suggest health professionals consider facilitating culturally appropriate follow-up care, such as via liaising with local Aboriginal and Torres Strait Islander Health Care Worker(s), local Aboriginal Community Controlled Health Services, using Aboriginal Medical Benefit Scheme entitlements, developing culturally-appropriate resources and potentially incorporating Aboriginal artworks in the appearance of offloading devices to personalise treatment. We suggest providing such culturally appropriate health care through a safe and welcoming clinical environment that is professional, humble, inclusive, transparent, respectful, empathetic, non-judgemental, and one that encourages choice, may help result in “Aboriginal and Torres Strait Islander people enjoying long and healthy lives” by preventing the “psychological distress” caused by DFU hospitalisation and disability [6–8].

Regardless of these implementation considerations for health professionals, we suggest there remains important access challenges to overcome before we see equitable wide-scale implementation of these guidelines throughout Australia. These challenges centre on the vast differences in access to and availability of offloading interventions (especially knee-high offloading devices and surgical procedures) that are governed by local funding restrictions and bureaucratic policies. Unfortunately, the ability to access these critical DFU treatments is still nearly entirely dependent on which Primary Health Network, Hospital and Health Service and/or State Health Department the patient resides as to what offloading treatment is provided and/or subsidised [3, 5, 37]. A recent large prospective real-world cohort of nearly 5000 Australians with DFU highlighted the critical importance to patients and services of overcoming any offloading treatment access challenges, when finding that knee-offloading device treatment was one of the only treatment factors that was positively associated with DFU healing after adjustment for multiple other demographic, comorbidity, limb, ulcer and treatment-related factors [11]. Whilst it is hoped that access to these knee-high offloading treatments should become much more readily available with the introduction of recent Australian High Risk Foot Service standards requiring services to provide these offloading treatments to be accredited [43], we still strongly suggest a nationally equitable scheme for patients to access best practice offloading treatments is urgently needed to reduce the national DFU burden [5, 15]. Given multiple (inter) national cost-effectiveness studies consistently demonstrate that gold standard knee-high offloading treatments are the most (cost-)effective intervention to heal people with DFU [46, 59], and reduce what is a leading cause of the national disability burden [4, 6], we suggest that there needs to be equitable national access to recommended offloading devices via a national publicly-funded scheme, such as Medicare Benefits Schedule or National Diabetes Services Scheme [5, 15, 37].

### Strengths and limitations

There are several strengths and limitations to note regarding the development of this guideline. The strengths included that we followed NHMRC-recommended ADAPTE and GRADE procedures for best practice adaptation of suitable international source guidelines [25–27], we identified and adapted the most recent international DFU guideline that was independently objectively assessed as being the highest-quality international guideline by ourselves and others [15, 21, 22] and the adaptation procedures were enacted by a transparent independent multi-disciplinary panel of (inter)nationally-recognised offloading experts in DFU care [28]. Further, unlike the international guideline we adapted [22], we also critically included consumer and Aboriginal and Torres Strait Islander experts in all aspects of guideline decision making, and comprehensively outlined implementation considerations when using the recommendations in this evidence-based guideline in accordance with GRADE [25–27].

However, this guideline is not without limitations, including we were reliant on the IWGDF systematic review identifying all the relevant available evidence in the field for us to review [15], and we were unable to review more recent evidence published since that 2019 review, which may have meant we missed important new evidence as part of our guideline deliberations [28]. However, we were able to re-review all identified IWGDF evidence and any additional recent Australian literature in which we were subsequently aware [28]. Further, as we followed a process to adapt IWGDF guidelines, we could not address any novel or alternate questions or undertake further systematic reviews. However, by adapting robust, high-quality guidelines, most if not all, of the topical questions can be considered covered in the current guideline. Additionally, although we did use a widely representative (inter)national expert panel in all decision making processes, and we engaged the perspectives of many Australian health professionals, researchers and peak bodies via a public call for consultation [28], we acknowledge that certain opinions and views may have been missed in this process. Lastly, whilst this guideline addresses recommendations in relations to questions regarding the best evidenced offloading treatment for those with a DFU, it does not address, and nor does the accompanying prevention guideline, questions relating to what offloading treatment should be recommended in the vital weeks and months after the patient heals to prevent recurrence [50]. We also strongly suggest that future guideline iterations address recommendations for best practice offloading treatment when transitioning from focussing on healing to prevention [50].

### Future research considerations summary

Despite the substantial new evidence published since the 2011 guideline, there is still high-quality evidence lacking for the majority of offloading treatments [15]. This is highlighted by the fact, that except for non-removable knee-high offloading devices, we rated all other recommendations as having (very) low quality of supporting evidence. This means the panel had low or very low levels of confidence that all other recommendations were based on studies that reported consistent effects with a low risk of bias and in turn further research was likely to change our confidence [29, 30]. Therefore, we agree with the IWGDF that there are multiple future research opportunities to significantly improve our understandings of the key benefits, risks, contraindications and feasibility of using different offloading treatments to heal people with DFU [15, 22].

Like IWGDF, we recommend future high-quality trials are still very much needed to test the effectiveness of all other offloading treatments (including removable offloading devices, footwear, other non-surgical interventions and surgical offloading procedures) against gold standard non-removable knee-high offloading device controls on multiple important outcomes including healing, plantar pressure, weight-bearing activity, adverse events, patient satisfaction, costs and particularly adherence [15, 22]. Behavioural interventions aimed at improving patient understanding and motivation regarding the use of offloading devices to improve adherence should also be a key focus of such future research. We also agree that such trials be conducted in accordance with IWGDF international reporting standards for high-quality DFU trials [35] and the CONSORT guideline [60], which should in turn enable future pooling of data for these outcomes and the opportunity for sub-group analyses to determine the patient (and foot) characteristics that benefit most (or least) from these specific interventions, such as in those complicated by infection or ischaemia or on different locations of the foot [15, 22]. Unfortunately, with the exception of one trial investigating the use of felted foam to offload and heal plantar DFU [53], to our knowledge no other trials of offloading interventions to heal people with DFU have been performed in Australia [15]. Thus, the panel encourages future Australian trials of offloading interventions adhering to the above trial standards and guidelines [35, 60], and particularly in Aboriginal and Torres Strait Islander populations and/or regions that are either geographically remote, have hot climates or dry environments, to determine if the effects found on healing in predominantly European and northern American trials are also found in Australia.

In addition to the above trials, the panel suggests future research into community perceptions of the benefits and risks of different offloading treatments are undertaken, such as those in qualitative studies to truly understand the patient perspective, particularly in geographically remote and Aboriginal and Torres Strait Islander peoples. We lastly suggest investigations into the effectiveness of the implementation of these guidelines in a range of different Australian environments, including in diverse patient groups, such as Aboriginal and Torres Strait Islander people are needed.

## Conclusion

When combined with other best practice DFU care, pressure offloading is a critical DFU treatment with the strongest evidence available to effectively heal foot ulcers and reduce the national burden of DFU. These new Australian guideline recommendations guide best practice offloading treatment in Australia and have been developed to suit the unique geography, diversity and needs of the Australian health professionals, sectors and patients. We have also outlined implementation strategies and future research priorities for offloading treatments in Australia. Thus, health professionals implementing these recommendations in Australia should impart better DFU knowledge, treatment and healing outcomes on their patients, communities and nation and in turn reduce the footprint of this devastating condition on the lives and livelihoods of Australians living with diabetes today and into the future.

## Supplementary Information

**Additional file 1.**

## Data Availability

Data sharing is not applicable to this article as no datasets were generated or analysed during the current study.

## Abbreviations

CONSORT: Consolidated Standards of Reporting Trials
DFD: Diabetes-related foot disease
DFU: Diabetes-related foot ulcers
DPN: Diabetes-related peripheral neuropathy
EtD: Evidence to Decision
GRADE: Grading of Recommendations Assessment, Development and Evaluation
IWGDF: International Working Group on the Diabetic Foot
NHMRC: National Health and Medical Research Council
PAD: Peripheral artery disease
RCT: Randomised controlled trial
TCC: Total contact cast
