# Supplementary material for: Australian guideline on offloading treatment for foot ulcers: part of the 2021 Australian evidence-based guidelines for diabetes-related foot disease
Source: J Foot Ankle Res. 2022 May 5;15:31. doi: 10.1186/s13047-022-00538-3 (PMC9069804; doi:10.1186/s13047-022-00538-3)
Supplement: Supplementary file 1 — Additional file 1. [file 13047_2022_538_MOESM1_ESM.docx]

**Online-Only Supplementary Material for:**

**Australian guideline on offloading treatment for foot ulcers: Part of the 2021 Australian evidence-based guidelines for diabetes-related foot disease**

Fernando ME, Horsley M, Jones S, Martin B, Nube VL, Charles J, Cheney J, Lazzarini PA, on behalf of the Australian Diabetes-related Foot Disease Guidelines & Pathways Project

**Table of contents Page**

**APPENDIX A: Tables of detailed justifications for recommendations** 3

**eTable A1:** Detailed justifications for Recommendation 1A 3

**eTable A2:** Detailed justifications for Recommendation 1B 5

**eTable A3:** Detailed justifications for Recommendation 2 7

**eTable A4:** Detailed justifications for Recommendation 3 9

**eTable A5:** Detailed justifications for Recommendation 4 11

**eTable A6:** Detailed justifications for Recommendation 5 14

**eTable A7:** Detailed justifications for Recommendation 6A 16

**eTable A8:** Detailed justifications for Recommendation 6B 19

**eTable A9:** Detailed justifications for Recommendation 9 21

**APPENDIX B: Tables of detailed considerations for recommendations** 23

**eTable B1:** Detailed considerations for Recommendation 1A 23

**eTable B2:** Detailed considerations for Recommendation 1B 26

**eTable B3:** Detailed considerations for Recommendation 2 28

**eTable B4:** Detailed considerations for Recommendation 3 30

**eTable B5:** Detailed considerations for Recommendation 4 32

**eTable B6:** Detailed considerations for Recommendation 5 34

**eTable B7:** Detailed considerations for Recommendation 6A 36

**eTable B8:** Detailed considerations for Recommendation 6B 39

**eTable B9:** Detailed considerations for Recommendation 7A 41

**eTable B10:** Detailed considerations for Recommendation 7B 42

**eTable B11:** Detailed considerations for Recommendation 7C 44

**eTable B12:** Detailed considerations for Recommendation 8 46

**eTable B13:** Detailed considerations for Recommendation 9 48

**REFERENCES**  49

**APPENDIX A: Tables of detailed justifications for recommendations**

## OFFLOADING DEVICES

#### **Recommendation 1A**

In a person with diabetes and a neuropathic plantar forefoot or midfoot ulcer, use a non-removable knee-high offloading device rather than a removable offloading device to promote healing of the ulcer (GRADE strength of recommendation: Strong; Quality of evidence: Moderate).

**eTable A1:** Detailed justifications for Recommendation 1A

| EtD criteria | Rating  (agreed*) | Detailed justifications |
| --- | --- | --- |
| Problem a priority | Yes  (+) | The panel agreed with the IWGDF that the available evidence supported that neuropathic plantar forefoot or midfoot DFU are both a serious and urgent health care problem to treat, and thus, a priority health problem internationally (1) and also in Australia (2). |
| Value of outcomes | Probably no important  uncertainty  (+) | The panel agreed with the IWGDF that there was probably no important uncertainty in how much patients value the outcome measures used to compare the intervention and control. This was based on the panel concluding that this recommendation reflects the belief that like international patients, most patients (and providers) in Australia, would also place most value (critical importance) on the outcome of healing their ulcer over other important but not critical DFU-related outcomes, such as plantar pressures, adverse events and patient satisfaction (1, 3). |
| Desirable effects  (Benefits) | Moderate  (?) | The panel could not determine if the IWGDF rating was for moderate or large desirable effects. However, the panel concluded that the available evidence supported that non-removable knee-high offloading devices were likely to have moderate additional desirable effects in comparison to removable offloading devices. This was based on the findings of several meta-analyses showing non-removable knee-high offloading devices compared with removable offloading devices were 17-43% significantly more likely, and 8-12 days faster, to heal neuropathic plantar forefoot or midfoot DFU after three months of use (1, 4). |
| Undesirable effects  (Risks) | Trivial  (+) | The panel agreed with the IWGDF that the available evidence supported that non-removable knee-high offloading devices were likely to have trivial additional undesirable effects compared to removable offloading devices. This was based on both devices reporting similar low adverse event incidences (0-20%) and similar but equivocal patient reported outcomes according to a small number of studies with a low quality of evidence for these outcomes (1, 4). Although, in the panel’s expert opinion, Australian patients may be less likely than international patients to prefer (or tolerate) using non-removable knee-high offloading devices in comparison to removable offloading devices because of reasons such as Australia’s hotter climate and the potential need for more frequent driving in Australia, and especially if they are not properly informed of the above desirable (benefits) and undesirable effects (risks). Thus, the panel felt it important that patients be fully informed of the above likely benefits as well as risks of using non-removable devices which may increase their likelihood of preferring (or tolerating) non-removable devices as has been previously identified in international literature (4, 5). |
| Balance of effects | Favours the intervention (+) | The panel agreed with IWGDF that the difference in balance of effects between the (moderate) additional desirable and (trivial) additional undesirable anticipated effects strongly favoured the non-removable knee-high offloading device over removable offloading devices for healing people with neuropathic plantar forefoot or midfoot DFUs (1). |
| Quality of evidence | Moderate  (-) | The panel disagreed with the IWGDF on the quality of supporting evidence and downgraded the IWGDF rating from high to moderate. This was based on the most recent, robust and homogenous (in terms of included intervention and control devices) meta-analysis published on this topic (4), that reported a moderate quality of evidence for custom-made non-removable knee-high offloading devices (i.e. total contact casts (TCCs)) compared to removable knee-high offloading devices (i.e. removable cast walkers) due to some inconsistency in findings across studies, plus, a low quality of evidence for pre-fabricated non-removable cast walkers compared with removable cast walkers due to some inconsistency in findings and baseline characteristics across studies (5). Thus, the panel was only moderately confident that the findings and low risk of bias of the collective evidence was consistent and rated the quality of evidence as moderate. |
| Acceptability | Probably yes  (+) | The panel agreed with the IWGDF that non-removable knee-high offloading devices would most probably be acceptable to most eligible (not contraindicated) patients and providers in most healthcare organisations that typically provide such treatment in Australia when patients were properly informed of the benefits and risks. This was based on the panel concluding that most Australian patients (and providers) would accept the evidence that the balance of effects favoured non-removable knee-high offloading devices, plus, that cost-effectiveness analyses conclude that they are less expensive over the duration of treatment compared with removable offloading devices (4, 5). |
| Feasibility | Probably yes  (+) | The panel agreed with the IWGDF that most patients (and providers) would find the non-removable knee-high offloading devices feasible to implement in most healthcare settings that typically provide such treatment in Australia. This was based on the panel concluding that most Australian healthcare organisations would be likely to choose to invest in non-removable knee-high offloading devices for patients in their care with such a serious and urgent problem (i.e. neuropathic plantar DFU), if they knew that these devices were more effective and cost-effective to heal those patients (4, 5). The panel also concluded that whilst some healthcare organisations may not have the expertise to use such devices at present, with recent Australian High Risk Foot Service standards requiring services to have such expertise available to be accredited (6), these devices should become even more readily available in the near future. |

*: +, agreed with IWGDF rating; ? unsure if agreed with IWGDF rating as IWGDF rating not clear or not reported; -, disagreed with IWGDF rating.

DFU; Diabetes-related foot ulcer; EtD: Evidence to decision; IWGDF:, International Working Group on the Diabetic Foot; TCC: Total contact cast

#### **Recommendation 1B**

When using a non-removable knee-high offloading device to heal a neuropathic plantar forefoot or midfoot ulcer in a person with diabetes, consider using either a total contact cast or nonremovable knee-high walker, with the choice dependent on the local resources and technical skills available, and the person’s preference and extent of foot deformity (Weak; Low).

**eTable A2:** Detailed justifications for Recommendation 1B

| EtD criteria | Rating  (agreed*) | Detailed justifications |
| --- | --- | --- |
| Problem a priority | Yes  (+) | The panel agreed with the IWGDF that the available evidence supported that neuropathic plantar forefoot or midfoot DFU are both a serious and urgent health care problem to treat, and thus, a priority health problem internationally (1) and also in Australia (2). |
| Value of outcomes | Probably no important  uncertainty  (+) | The panel also agreed with the IWGDF that there was probably no important uncertainty in that most patients (and providers) om Australian would also place most critical value on the outcome of healing their ulcer over other important outcomes (1, 3). |
| Desirable effects | Trivial  (?) | The panel felt trivial additional desirable effects, can be anticipated from using TCCs compared to non-removable knee-high walkers, such as similar healing rates but were unsure if they agreed with the IWGDF as we could not determine if the IWGDF rating was for trivial or small desirable effects. However, the panel concluded that the available evidence indicated that the difference in desirable effects between TCCs and non-removable knee-high walkers was trivial. This was based on one recent high-quality meta-analysis (5) and four individual RCTs of differing quality, all finding no significant difference in ulcers healed and time-to-healing between these devices after three months of use (1, 4). We also agreed with the IWGDF that the available evidence, although of low quality, indicated that the surrogate outcomes of plantar pressure reductions and ulcer area reductions also showed no differences between either of these devices (1, 4). However, these studies contained small sample sizes. Hence, we agreed with the IWGDF that none of the studies were powered to test for equivalence and thus they may have been underpowered to detect a difference. |
| Undesirable effects | Trivial  (+) | The panel agreed with the IWGDF that the available evidence supported that the difference in undesirable effects between non-removable knee-high offloading devices and removable offloading devices was also trivial. This was based on the above studies reporting both devices had statistically similar and relatively low incidence of adverse events (0-30%), patients choosing to discontinue treatment (9-20%), and similar but equivocal patient-reported outcomes according to a small number of studies with a low quality of evidence for these outcomes (1, 4, 5). We also agreed with the IWGDF, and a recent large cost-effectiveness analysis (5), that the cost per patient for the duration of treatment is likely to be more expensive in the TCC intervention compared to the non-removable knee-high walkers control, mostly due to gaining similar outcomes with less expenditure on materials when using the non-removable knee-high walkers control (1). Thus, the panel felt the difference in undesirable effects was trivial for the two devices. |
| Balance of effects | Does not favour either intervention or control  (+) | The panel agreed with the IWGDF that the difference in balance of effects does not favour either the TCC or non-removable knee-high walkers as they both produce similar desirable (benefits) and similar undesirable effects (risks) for healing people with neuropathic plantar forefoot or midfoot DFUs (1). |
| Quality of evidence | Low  (-) | The panel disagreed with the IWGDF on the quality of supporting evidence and downgraded the IWGDF rating from moderate to low. This was based on the most recent, robust and homogenous (in terms of included intervention and control devices) meta-analysis published on this topic (4) reporting a low quality of evidence for TCCs compared with non-removable knee-high walkers due to very low statistical power to detect a statistical difference between groups (5). Thus, the panel had low confidence in the certainty of the findings from the available evidence and rated the quality of evidence as low. |
| Acceptability | Probably yes  (?) | The panel was unsure if they agreed with the IWGDF as it was unclear as to what the IWGDF acceptability rating was for this recommendation. However, in the judgement of the panel we suggest that most patients (and providers) in most healthcare organisations that typically provide such treatment would probably find either device acceptable. This was based on the panel concluding that while most Australian patients (and providers) would accept the evidence that the balance of effects would be similar between these devices. However, these devices’ effect on autonomy would vary depending on the extent of their foot deformity and personal preference. Thus, we again could not make a decision regarding superiority of one type of non-removable knee-high offloading device as it would depend on the patient’s circumstances and preferences, and the resources available to their treating organisation to apply, review and remove the device as needed. (4, 5) |
| Feasibility | Probably yes  (?) | Again, the panel was unsure if they agreed with the IWGDF as the IWGDF rating was unclear regarding feasibility. However, in the judgement of the panel, the use of prefabricated removable knee-high walkers (made irremovable) have largely (although not completely) replaced TCC in the treatment of DFU due to their accessibility, ease of application and lack of skill needed to apply. Additionally, non-removable walkers were found to be more cost-effective than TCCs (5). However, when required, and particularly for those patients with large foot deformities that are unable to be accommodated in a prefabricated walker, such as a very wide foot, plantigrade foot, a large Charcot foot, or extensive bunion, that TCCs should be chosen. We also consider access to TCC is also improving with implementation of Interdisciplinary High Risk Foot Service accreditation standards of care in Australia requiring services demonstrate access to TCCs and walkers (6). Thus, we suggest that using both these devices by eligible patients (and their providers) is probably feasible. However, the panel also agreed with IWGDF that the decision regarding the use of non-removable cast walker or TCC is dependent on a number of other factors; the level of expertise to apply, review and remove both types of non-removable devices that the service providing such treatment possesses, and the extent (size) of foot deformity to be accommodated in the device and individual patient preferences. |

* +, agreed with IWGDF rating; ? unsure if agreed with IWGDF rating not clear or not reported; -, disagreed with IWGDF rating.

DFU; Diabetes-related foot ulcer; EtD: Evidence to decision; IWGDF:, International Working Group on the Diabetic Foot; TCC: Total contact cast

#### **Recommendation 2**

In a person with diabetes and a neuropathic plantar forefoot or midfoot ulcer, when nonremovable knee-high offloading devices are contraindicated or not tolerated, consider using a removable knee-high offloading device (and explain the importance of using) during all weight-bearing activities rather than a removable ankle-high offloading device to reduce plantar pressure and promote healing of the ulcer (Weak; Low).

**eTable A3:** Detailed justifications for Recommendation 2

| EtD criteria | Rating  (agreed*) | Detailed justifications |
| --- | --- | --- |
| Problem a priority | Yes  (+) | The panel agreed with the IWGDF that the available evidence supported that neuropathic plantar forefoot or midfoot DFU are both a serious and urgent health care problem to treat internationally (1) and also in Australia (2). |
| Value of outcomes | Possibly important uncertainty  (-) | The panel disagreed with the IWGDF in that there may be possibly important uncertainty or variability in how much patients value ulcer healing compared to their autonomy. The panel therefore wanted to emphasise patient involvement in the decision making regarding whether to use a knee-high or ankle-high removable offloading device and to ensure that it does not lead to postural instability and a detrimental impact on the quality of life of the person using the device (7, 8). The panel agreed that an individual decision needs to be made regarding whether the user values their independence and safe mobility over rapid healing (1). However, the panel agreed that most patients (and providers) would place more critical value on the outcome of healing their ulcer in Australia over other important outcomes (1). |
| Desirable effects | Moderate  (+) | The panel agreed with the IWGDF that currently there is no robust evidence of benefit of a removable knee-high device compared to removable ankle-high devices to show superiority in DFU healing outcomes in the most robust meta-analysis of the evidence to date (5, 9). However, there was evidence from a high-quality RCT, and multiple cross-sectional studies, to show that removable knee high offloading devices were associated with a greater reduction in plantar pressures (10) compared to a removable ankle high device and some evidence from several RCTs of varying quality to show that activity levels (11) may be reduced compared to a removable ankle high devices, but findings were inconsistent. Thus, the panel agreed with the IWGDF that a greater plantar pressure relieving effect is likely to offer a greater probability of healing for those wearing removable knee-high offloading devices if the devices are worn for the same amount of time/activity (1, 4). |
| Undesirable effects | Trivial-small  (?) | The panel had similar judgements on undesirable effects to the IWGDF in that the available evidence supported that the difference in undesirable effects between removable knee-high offloading devices and other removable offloading devices was trivial and there is no clear difference in adverse events between removable knee- and ankle-high offloading devices and in other secondary outcomes (1). We agree with IWGDF that these devices will mostly have similar low adverse events, although the ankle‐high offloading devices may potentially have fewer adverse events compared with knee‐high offloading devices (1) as they either have lower or no device walls that reduce the risk for abrasions, lower‐leg ulcers, imbalance, and gait challenges (12). However whether this was true was unclear from individual studies as the limited evidence suggested descriptively similar adverse events when using both types of devices within the population, but given the potential for recruitment bias and risk of false negatives in previous clinical trials (due to small sample sizes) and the exclusion of patients for whom a greater than average risk existed this is not entirely certain (5, 9). |
| Balance of effects | Probably favours intervention (+) | The panel agreed with the IWGDF that the difference in balance of effects probably favours the removable knee high device compared to other types of devices based predominantly on moderate desirable effects of the secondary outcomes of plantar pressure and activity levels (10, 11) compared to other removable devices, as they both produce similar desirable (benefits) and similar undesirable effects (risks) for the primary outcome of healing in people with neuropathic plantar forefoot or midfoot DFU (1). |
| Quality of evidence | Low  (+) | The panel agreed with the IWGDF on the quality of supporting evidence as a “low” quality of evidence. This was based on the most recent, robust and homogenous (in terms of included intervention and control devices) meta-analysis published on this topic (4), that reported a low quality of evidence (5). Thus, the panel had low confidence in the certainty of the findings from the available evidence and rated the quality of evidence as low. |
| Acceptability | Yes  (+) | The panel agreed with the IWGDF that either a removable knee-high offloading device or ankle high offloading device would be most probably acceptable to most patients (and providers) in most healthcare organisations that typically provide such treatment. Given the lack of power to detect a difference in acceptability and adherence in prior studies (10, 11), the panel decided there is insufficient evidence of large benefit to convince all providers that one form of removable offloading is substantially better than other forms of ankle high offloading when patient acceptability and the impact the intervention has on their quality of life is considered (13). Thus, we again could not make a decision regarding superiority of one type of removable offloading device as it would vary depending on the patient’s value of outcomes, circumstances and preferences. (4, 5). |
| Feasibility | Probably yes  (+) | We suggest that there are probably no important barriers that are likely to limit the feasibility of implementing the removable knee-high devices or require consideration when implementing and it should be sustainable if implemented long term. This was based on the panel concluding that most Australian healthcare organisations would probably invest in both removable knee and ankle-high offloading devices for patients with such a serious and urgent problem (i.e., neuropathic plantar DFU) once they knew that these devices were likely to be equally effective to heal those patients (4, 5), but that users may prefer one device over the other. The panel agreed that both interventions are sustainable and that there is probably no important barriers that are likely to limit the feasibility of implementing the intervention or require consideration when implementing it. |

*Note: +, agreed with IWGDF rating; ? unsure if agreed with IWGDF rating not clear or not reported; -, disagreed with IWGDF rating.

DFU; Diabetes-related foot ulcer; EtD: Evidence to decision; IWGDF:, International Working Group on the Diabetic Foot; TCC: Total contact cast

#### **Recommendation 3**

In a person with diabetes and a neuropathic plantar forefoot or midfoot ulcer, when knee-high offloading devices are contraindicated or not tolerated, use a removable ankle-high offloading device (and explain the importance of using) during all weight-bearing activities rather than medical grade footwear to promote healing of the ulcer (Strong; Very low)

**eTable A4:** Detailed justifications for Recommendation 3

| EtD criteria | Rating  (agreed*) | Detailed justifications |
| --- | --- | --- |
| Problem a priority | Yes  (+) | The panel agreed with the IWGDF that the available evidence supported that neuropathic plantar forefoot or midfoot DFU are both a serious and urgent health care problem to treat internationally (1) and also in Australia (2). |
| Value of outcomes | Probably no important uncertainty  (+) | The panel also agreed with the IWGDF that there was probably no important uncertainty in that most patients (and providers) would place most critical value on the outcome of healing their ulcer in Australia over other important outcomes (1) and that there is probably no important uncertainty or variability in critical surrogate outcomes and other secondary outcomes. |
| Desirable effects | Varied, but likely to be moderate  (-) | The panel noted that no controlled studies specifically comparing removable ankle‐high devices to conventional or standard therapeutic footwear or other offloading interventions, for effectiveness of healing, surrogate healing outcomes, adverse events, patient preferences, or costs was found in the most up to date systematic review to date (4). However, several noncontrolled studies show that 70% to 96% of plantar foot ulcers can be healed in a reasonable time frame (mean 34‐79 days) with ankle‐high removable offloading devices and that such devices may be better at reducing plantar pressures compared to a variety of therapeutic footwear types, provided they are used regularly (14-19)**.** However, these findings were reported in uncontrolled studies and contained small sample sizes, the control groups were given various types of footwear (not always therapeutic footwear) and therefore the robustness of these findings are unclear given the high risk of bias and substantial heterogeneity. Importantly, there was a range of devices included under the umbrella heading of “removable ankle-high devices” which included ankle‐high cast walkers, cast shoes, half shoes, forefoot offloading shoes, postoperative healing shoes, and custom‐made temporary shoes (1). Furthermore, there is limited data on which to evaluate comfort and acceptance of removable ankle-high offloading devices compared to therapeutic footwear (10, 20) and there was no data available on cost-effectiveness (1). Given these reasons, the expert panel decided that the likely desirable effect of removable ankle-high offloading devices is varied. However, given that a majority of plantar foot ulcers can be healed in a reasonable time frame (mean 34‐79 days) with ankle‐high removable offloading devices (1) the panel decided there was a “strong” recommendation for the intervention. If medical-grade shoes with an appropriate orthotic device is accessible and has demonstrated superior pressure offloading and patient acceptance to other alternative ankle high devices, then use the medical-grade footwear. |
| Undesirable effects | Trivial  (+) | The panel agreed with the recommendation of the IWGDF that there is likely to be only a small effect (difference) for the critical primary outcomes (e.g., healing) for which there is an undesirable effect and similarly for the critical surrogate outcomes (e.g., ulcer area change, plantar pressure change etc) and for the other secondary outcomes (e.g., adverse events, patient satisfaction, cost-effectiveness, etc) (1). The panel however felt that certain types of ankle-high offloading devices such as a half-shoe may lead to a high risk of falls compared to therapeutic footwear despite its superior plantar pressure reduction effect given that the half-shoe is also associated with altered gait and a reduced propulsion in late stance (21). Given an already compromised gait in people with DFU (22), the expert opinion of the panel was that extra-caution needs to be applied in using this particular type of removable ankle-high offloading device until further research becomes available given the risk for falls (23). |
| Balance of effects | Favours the intervention  (+) | The panel agreed with the IWGDF that the difference in balance of effects probably favours the removable ankle-high devices compared to therapeutic footwear after weighing the potentially higher healing benefits of removable ankle-high devices over conventional or therapeutic footwear and the reported better outcomes on plantar pressure (14-19) **w**ith expected similar low incidence of harms and patient preferences (10, 20) in the limited literature to date (1). However, the panel felt that there were too many diverse interventions included in the ankle-high offloading interventions which may lead to misrepresentation of a true effect. Hence the overall balance of effect is hard to ascertain due to paucity of evidence but except for half shoes, most ankle high devices are well tolerated without any serious adverse events with the benefit of pressure offloading to support healing. The panel agreed with the IWGDF that ankle-high devices may be associated with better adherence compared to knee high devices (8) and for low resource settings and areas with limited trained expertise and cast technicians, these removable ankle-high devices may be an appropriate offloading intervention for treating some patients with plantar neuropathic forefoot ulcers (1). |
| Quality of evidence | Very low  (?) | The panel disagreed with the quality of evidence reported by the IWGDF as a “low” quality of evidence and downgraded this to a “very low” level of evidence. The panel disagreed with the IWGDF regarding the certainty of evidence and they felt that there was limited data to be certain that one type of device is superior to others. Given there are no randomised controlled trials evaluating the role of removable ankle-high offloading devices in comparison to therapeutic footwear and as the foundation for the recommendations are based on non-controlled clinical data on a small number of patients from a select number of continents and largely based on expert opinion, this was perceived by the panel to be of a very low quality and the applicability of these findings to Australia was unclear. The panel felt that in comparing ankle-high devices to therapeutic footwear with a highly variable pressure offloading outcome, the relative benefit (if worn more consistently) has not been assessed. Until further evidence becomes available, the quality of evidence remains “very low”. |
| Acceptability | Yes  (+) | The panel agreed with the IWGDF on the overall acceptability for using a removable ankle-high offloading device by most patients (and providers) in most healthcare organisations that typically provide such treatment. There is limited data on which to evaluate comfort and acceptance of removable ankle-high offloading devices compared to therapeutic footwear (10, 20) and there was no data available on cost-effectiveness (1). These remain important areas with limited evidence and future research may change the overall acceptability of using a removable ankle-high offloading device by most patients (and providers). |
| Feasibility | Yes  (+) | The panel also agreed with the IWGDF that the availability of ankle-high device is likely to be less of a barrier compared to the availability of custom-made therapeutic footwear within Australian sites (1). Given the current Australian context and the variability of access to custom made therapeutic footwear most Australian healthcare organisations are likely to invest in removable ankle-high offloading devices for patients with such a serious and urgent problem (i.e., neuropathic plantar DFU) once they knew that these devices were effective to heal DFU (4, 5). The panel agreed that both interventions are sustainable and that there is no probably no important barriers that are likely to limit the feasibility of implementing the intervention or require consideration when implementing it. |

* +, agreed with IWGDF rating; ? unsure if agreed with IWGDF rating; -, disagreed with IWGDF rating.

DFU; Diabetes-related foot ulcer; EtD: Evidence to decision; IWGDF:, International Working Group on the Diabetic Foot; TCC: Total contact cast

## FOOTWEAR

#### **Recommendation 4**

In a person with diabetes and a neuropathic plantar forefoot or midfoot ulcer, when ankle-high offloading devices are contraindicated or not tolerated, use medical grade footwear rather than other footwear types or no footwear to promote healing of the ulcer (Strong; Low).

**eTable A5:** Detailed justifications for Recommendation 4

| EtD criteria | Rating  (agreed*) | Detailed justifications |
| --- | --- | --- |
| Problem a priority | Yes  (+) | The panel agreed with the IWGDF that the available evidence supported that neuropathic plantar forefoot or midfoot DFU are both a serious and urgent health care problem to treat internationally (1) and also in Australia (2). |
| Value of outcomes | Possible important uncertainty  (-) | While the panel agreed with the IWGDF that most patients (and providers) were likely to place most critical value on the outcome of healing their ulcer in Australia over other important outcomes (1), we also considered that there was a level of possibly important uncertainty as well as some patients may equally value surrogate and other secondary outcomes due to limited evidence on healing when using therapeutic footwear in people with DFU due to implications on plantar pressure, activity levels, costs, patient satisfaction and adverse events i.e. secondary ulceration and risk of infection as reported in studies (24-26). Additionally, a recent high-quality meta-analysis reported significantly more treatment discontinuations in those patients treated with nonremovable knee-high offloading devices compared to therapeutic footwear (5). |
| Desirable effects | Don’t know, but likely to be moderate  (-) | We disagreed with the IWGDF that the desirable effects were clear regarding the efficacy of therapeutic footwear compared to no footwear or other types of footwear given the paucity of evidence and given it is dependent on the specific device used as well as patient factors (1). However, we agreed with the IWGDF that there is high quality evidence to show that nonremovable knee‐high offloading devices were 62% to 68% more likely to heal a neuropathic plantar forefoot ulcer than therapeutic footwear based on two high quality meta-analyses (5, 27), that included three studies reporting ulcer healing in 68% to 90% of patients in the nonremovable knee-high offloading group and 32% to 56% in the therapeutic footwear group after 3 to 4 months of follow-up (5). The same high quality meta-analysis reported that therapeutic footwear was far less cost‐effective than other nonremovable and removable knee-high offloading devices (5). However, the panel’s expert opinion is that although therapeutic footwear is much less effective than knee-high offloading devices, where therapeutic footwear is the only option available to use, then in our expert opinion therapeutic footwear (known as medical grade footwear in Australia) would be likely to offer moderate additional desirable effects on healing, as many cross-sectional studies show small-to-large desirable effects on plantar pressure reduction when using medical grade footwear compared to other footwear or no footwear. |
| Undesirable effects | Don’t know, but likely to be trivial  (-) | The panel disagreed with the recommendation of the IWGDF that there is likely to be a small effect (difference) for the secondary outcomes (e.g., adverse events, patient satisfaction, cost-effectiveness, etc) (1). The panel felt that as there was no data to show the undesirable effects of therapeutic footwear in healing DFU when compared to no intervention or when compared to other footwear(1), conclusions could not be reached based on the evidence available. However, based on a recent high-quality meta-analysis reporting significantly more treatment discontinuations in those patients treated with nonremovable knee-high offloading devices compared with therapeutic footwear (5), and in our expert opinion that as medical grade footwear is customised to the patient’s foot, then our opinion is that medical grade footwear is likely to produce trivial additional undesirable effects compared with other footwear types or no footwear at all (barefoot). |
| Balance of effects | Favours the intervention  (+) | The panel agreed with the IWGDF that based nearly entirely on our expert opinion that the difference in balance of effects is most likely to favour medical grade footwear over other footwear types or no footwear based on moderate desirable effects on plantar pressure (and in turn potentially healing) and trivial undesirable effects on adverse events etc The panel’s expert opinion is that such a comparison would swing the balance of effects in favour of therapeutic footwear and this may be an appropriate offloading intervention for treating plantar neuropathic forefoot ulcers in the absence of availability of offloading device interventions (1). However, the panel agreed that the balance of effects from the available evidence between the desirable (benefits) and undesirable (risks) effects clearly favours the nonremovable knee-high device compared to therapeutic footwear, given that nonremovable knee‐high offloading devices were 62% to 68% more likely to heal a neuropathic plantar forefoot ulcer than therapeutic footwear based on two high quality meta-analyses (5, 27). Thus, given there is moderate evidence for knee-high offloading devices being much more effective than therapeutic footwear to heal DFU, but an absence of evidence comparing therapeutic footwear to other footwear types for obvious ethical reasons, the panel agreed the in circumstances where no offloading devices are available then medical grade footwear would be much more preferable to use than other footwear types or no footwear at all. |
| Quality of evidence | Low  (-) | The panel disagreed with the IWGDF regarding the certainty of evidence as we felt that there was no data to show the efficacy of therapeutic footwear in healing DFU when compared to no intervention or when compared to other footwear types (1). Although the IWGDF evidence quality was rated as “moderate” given the number of high quality meta-analysis included that demonstrated knee-high offloading devices were much more effective than therapeutic footwear (27), the panel downgraded this to “low” given a paucity of studies reporting on the use of therapeutic footwear in people with DFU compared to no footwear or other footwear types. The panel disagreed with the IWGDF regarding the certainty of evidence for using therapeutic footwear. Importantly, specific medical grade footwear modifications such as cushioning, rigidity of the outer sole, whether it has a rigid rocker and the nature of the insole, cushioning or padding used in conjunction within the footwear are likely to all contribute to a reduction in plantar pressures and therefore a benefit in ulcer healing, yet there is a lack of evidence regarding these interventions to heal DFU (28). |
| Acceptability | Probably yes  (+) | Therapeutic footwear is likely to be acceptable when compared to no intervention, but unlikely to be acceptable when compared to knee-high offloading options but in general may be less well tolerated. Therefore, therapeutic footwear should only be used in specific circumstances where no other offloading devices are available for the healing of a DFU. The panel agreed with the IWGDF on the overall acceptability for using therapeutic footwear by most patients (and providers) in most healthcare organisations that typically provide such treatment. One low‐quality RCT reported on patient preference and found that those patients using nonremovable knee high devices and those using therapeutic footwear had no difference in acceptance of treatment scores (26). Therefore, most patients and providers would accept that the desirable effects would in general outweigh the undesirable effects of using therapeutic footwear compared to no footwear as DFU healing is the primary outcome. |
| Feasibility | Probably yes  (+) | The panel also agreed with the IWGDF that the intervention is probably likely feasible to implement, however availability of custom-made therapeutic footwear within Australian sites is likely to be a barrier (1). While the panel felt that the intervention would be sustainable to implement from a supply perspective through funded and supported schemes to subsidise footwear and availability of a range of footwear, the expertise to assess suitability and prescribe appropriate footwear is not consistent across Australian states and territories. Therefore, access to suppliers (geographically and/or financially) is likely a limiting issue for some patients. The panel felt that lack of consistent national policy in relation to supply of therapeutic footwear at the present time is likely to be a barrier to the feasibility of using this intervention. This may be an important future consideration. |

Note: +, agreed with IWGDF rating; ? unsure if agreed with IWGDF rating; -, disagreed with IWGDF rating.

DFU; Diabetes-related foot ulcer; EtD: Evidence to decision; IWGDF:, International Working Group on the Diabetic Foot; TCC: Total contact cast

## OTHER (NON-SURGICAL) OFFLOADING TECHNIQUES

#### **Recommendation 5**

In a person with diabetes and a neuropathic plantar forefoot or midfoot ulcer, consider using felted foam in combination with an offloading device or footwear rather than using the offloading device or footwear alone to further reduce plantar pressure and promote healing of the ulcer (Weak; Very Low).

**eTable A6:** Detailed justifications for Recommendation 5

| EtD criteria | Rating  (agreed*) | Detailed justifications |
| --- | --- | --- |
| Problem a priority | Yes  (+) | The panel agreed with the IWGDF that the available evidence supported that neuropathic plantar forefoot or midfoot DFU are both a serious and urgent health care problem to treat internationally (1) and also in Australia (2). |
| Value of outcomes | Probably no important uncertainty  (+) | The panel also agreed with the IWGDF that there was probably no important uncertainty in that most patients (and providers) would place most critical value on the outcome of healing their ulcer in Australia over other important outcomes (1) and that there is probably no important uncertainty or variability in critical surrogate outcomes and other secondary outcomes. |
| Desirable effects | Small-moderate  (+) | Firstly, we note for the Australian reader that studies on felted foam and felt only were considered and reported collectively under the category of “felted foam” by IWGDF, and thus felt is considered as a type of felted foam for this recommendation (1, 4). We agreed with the IWGDF that the desirable effects are likely to be small-to-moderate given a reported shorter time to healing when felted foam has been used in combination with an ankle-high offloading device compared to using a different ankle-high offloading device without felt foam in one study (29), no trials on footwear though, and a reduction in plantar pressures reported in other clinical studies (30, 31). Given there are no controlled studies that investigated patient preferences or costs in the most recent systematic review on the topic (4), we agree with the IWGDF on their expert opinion that users and clinicians will likely value and prefer the use of felted foam as an easy‐to‐use modality given that costs are relatively low (1). |
| Undesirable effects | Trivial-small  (+) | Given that the risk of adverse outcomes were similar when using felted foam in combination with an offloading device or in an offloading device alone including minor skin tear/maceration (10% vs 20%) and foot infection (25% vs 23%) (29, 32), we mostly agree with the IWGDF that undesirable effects are likely to be trivial. However, we also felt that the risk of maceration may be greater in certain Australian populations in hotter climates. Also an additional undesirable effect of this treatment is that it requires frequent replacement, by a clinician, the patient, a relative, or a home‐care nurse which may add to the frequency of visits for both the patient and clinician. This is an important consideration in the Australian context (see subgroup considerations). We also agree with the IWGDF that whether the felted foam is fitted to the foot or to the shoe or insole may not make a difference in healing based on a single trial showing no difference in healing (32). |
| Balance of effects | Probably favours intervention (+) | The panel therefore agreed with the IWGDF that when evaluating the effect of felted foam in combination with offloading devices and its effects on DFU healing (16, 29, 32) and plantar pressure (30, 31) and a low risk of adverse effects (29, 32) reported, that the balance of effects probably favours the intervention of felted foam in combination with offloading devices compared to no felted foam or felted foam used in isolation (1). Based on our expert opinion, we also agreed that the balance of effects of using felted foam with footwear is also likely to favour this over footwear alone also. |
| Quality of evidence | Very low  (-) | The panel disagreed with the “low” quality of evidence rating of the IWGDF and downgraded this to a “very low” quality of evidence, given this is based on one small low-quality RCT using offloading devices only, no trials reported for footwear and several cross-sectional studies showing small-moderate further reductions in plantar pressure when using felted foam compared with not using felted foam. Thus, the panel felt the quality of evidence should be “very low”. |
| Acceptability | Probably yes  (+) | The panel agreed with the IWGDF that although there are no trials that investigated patient preferences or costs on the use of felted foam, the use of felted foam within offloading devices or footwear is likely to be acceptable to most patients and clinicians in Australia. This acceptability rating seems to be supported by two surveys of Australian clinicians reporting that clinicians often use felted foam in their offloading treatment of people with DFU (33, 34). |
| Feasibility | Probably yes  (+) | The panel also agreed with the IWGDF that the availability of felted foam is unlikely to be a major barrier compared to the availability of other interventions within Australian sites, especially given the low cost of utilising this intervention (1). Given the current Australian context, the panel’s opinion was that most healthcare organisations are likely to invest in felted foam for patients with such a serious and urgent problem (i.e., neuropathic plantar DFU) once they knew that felted foam may assist with DFU healing and plantar pressure reduction. The panel agreed that the intervention was sustainable and that there are probably no important barriers that are likely to limit the feasibility of implementing the intervention or require consideration when implementing it. |

Note: +, agreed with IWGDF rating; ? unsure if agreed with IWGDF rating; -, disagreed with IWGDF rating.

DFU; Diabetes-related foot ulcer; EtD: Evidence to decision; IWGDF:, International Working Group on the Diabetic Foot; TCC: Total contact cast

## SURGICAL OFFLOADING TECHNIQUES

#### **Recommendation 6A**

If the best recommended offloading device option fails to heal a person with diabetes and a neuropathic plantar metatarsal head ulcer, consider using Achilles tendon lengthening or Gastrocnemius recession, metatarsal head resection(s), or joint arthroplasty to promote healing of the ulcer (Weak; Low).

**eTable A7:** Detailed justification for Recommendation 6A

| EtD criteria | Rating  (agreed*) | Detailed justifications |
| --- | --- | --- |
| Problem a priority | Yes  (+) | The panel agreed with the IWGDF that the available evidence supported that neuropathic plantar metatarsal head DFU that had failed to heal using non-surgical offloading interventions are both a serious and urgent health care problem to treat internationally (1) and also in Australia (2). |
| Value of outcomes | Probably no important uncertainty  (+) | The panel also agreed with the IWGDF that there was probably no important uncertainty in that most Australian patients (and providers) would place most critical value on the outcome of healing their ulcer over other important outcomes (1). |
| Desirable effects | Moderate  (+) | The panel agreed with the IWGDF that collectively for the three surgical offloading procedures that there were moderate desirable effects, i.e. Achilles tendon lengthening (ATL), metatarsal head resections (MTH resections) and joint arthroplasty procedures. Additionally, the panel considered that Gastrocnemius Recession (GR) also had moderate desirable effects. For ATL procedures, this was based on the findings of one high-quality RCT and meta-analyses showing a non-significant trend for ATL (plus TCCs) to be 6-13% more likely, and up to 14 days faster, to heal these DFU after 7 months of use compared with TCC offloading devices alone (35, 36). However, it should be noted that one low-quality RCT in the meta-analysis used either ATL or GR procedures in their trial and reported 14 days faster healing (36, 37). Furthermore, one high-quality RCT found a 27-42% significant reduction in forefoot plantar pressure compared to a TCC control, three-weeks after undergoing an ATL intervention, but this plantar pressure difference did not persist after 8 months (38). Similar effects on healing and plantar pressure outcomes were also seen in other non-controlled case series using ATL and GR procedures (1, 4). For MTH resection, four controlled studies of various quality found MTH resections (plus, a variety of other non-gold standard offloading interventions) were 16-40% more likely, and 21-350 days faster, to heal these DFU after a variety of follow-up times compared with a variety of other non-gold standard offloading interventions alone (1, 4). However, it should be noted that the higher desirable effects were reported in the low-quality studies and thus the real effect is likely to be at the lower end of the above reported range (1, 4). For joint arthroplasty, two low-quality controlled trials found joint arthroplasty (plus non-removable offloading devices) healed these DFU 24-43 days faster than a non-removable offloading device alone (1, 4). Finally, although most adverse event outcomes were similar between the surgical offloading procedures and non-surgical offloading controls (devices or footwear), the four controlled studies investigating MTH resection showed a 4-47% significant decrease in developing infection adverse events in those undergoing MTH resection (1, 4). Therefore, collectively we assessed these four surgical offloading procedures (plus offloading devices) had moderate desirable effects compared to offloading devices alone on healing plantar metatarsal head ulcers in those who had failed to heal using offloading devices to date. |
| Undesirable effects | Small  (+) | The panel agreed with the IWGDF that collectively for the three surgical offloading procedures, plus again in our view GR procedures, that there were small undesirable effects. With the exception of the potentially moderate desirable effect on infection adverse events identified above, this was based on surgical and non-surgical offloading interventions reporting similar low adverse events incidences (0-18%) in the above controlled trials (1, 4), except for ATL procedures having a 13% significant increase in new heel ulcers develop compared to TCCs (1, 4, 35, 38). However, this increased heel ulcer rate was not found for similar GR procedures in several other non-controlled case series which found no heel ulcer adverse events (4, 39, 40), unlike other ATL case series which found heel ulcers developed in 15-21% of patients (4, 41, 42). This difference in heel ulcer adverse event rates may be explained by ATL procedures potentially overlengthening intra-operatively, whereas GR procedures potentially protect against overlengthening and preserve soleal function therefore reducing load transfer to the heel during gait and reducing the risk of heel ulceration. (39, 40). Overall, in recognition of the inherent increased risk of any surgical procedure compared with a non-surgical intervention, that the population concerned were those that had already failed non-surgical offloading interventions, and the increased risk of heel ulcers in those undergoing ATL procedures, the panel collectively agreed these surgical offloading procedures (plus offloading devices) had small undesirable effects compared to non-surgical offloading devices alone. |
| Balance of effects | Probably favours the intervention(s) (+) | The panel agreed with the IWGDF that the difference in balance of effects between the (moderate) desirable and (small) undesirable effects probably collectively favoured the surgical offloading procedures over other non-surgical offloading interventions (typically devices or footwear) for healing neuropathic plantar metatarsal DFU that had failed to previously heal using other non-surgical offloading interventions alone (1). As mentioned above, the panel considered this small positive balance of effects not only favoured ATL, MTH resections and joint arthroplasties, but also GR procedures as well. |
| Quality of evidence | Low  (+) | The panel agreed with the IWGDF that the collective quality of supporting evidence was low (1). This was based on there being only a few controlled trials of mostly low quality for each of the four surgical procedures, and those of highest quality reported the lowest desirable effects (and vice versa) (1, 4). Thus, the panel had low confidence in the certainty of the findings from the available evidence and rated the quality of evidence as low. |
| Acceptability | Probably yes  (+) | The panel agreed with the IWGDF that using these surgical offloading procedure interventions would probably be acceptable to most patients (and providers) that had previously failed to heal their plantar metatarsal head ulcers using non-surgical offloading interventions (or when pressure offloading cannot be achieved due to the severity of contracture) in most of the healthcare organisations that typically provide such treatment (1). This was based on the only controlled trial measuring patient satisfaction finding that, whilst patients undergoing MTH resections (plus therapeutic footwear) were ~40% less satisfied with their discomfort during treatment, they were ~30% more satisfied at the conclusion of treatment than those provided with therapeutic footwear treatment only (4, 43). Furthermore, the panel felt that although these surgical procedures would probably adversely affect the patients’ autonomy in the short-term post-operatively, most Australian patients (and providers) would probably accept the evidence that the balance of effects would be in favour of these surgical offloading procedures in non-healing circumstances, that they are also likely to accept the post-operative care required as such care is usually a similar offloading device to that they had previously worn, and finally no patients are likely to disapprove morally. Thus, we concluded these surgical procedures are probably acceptable to most Australian patients (and providers) in these circumstances (4). |
| Feasibility | Probably yes  (?) | The panel was unsure if they agreed with the IWGDF as it was unclear as to what the IWGDF feasibility rating was for this recommendation. However, we suggest that using, or at least referring for, these surgical offloading procedures would probably be feasible in most healthcare organisations that patients attended for such treatment in Australia. This was based on the panel concluding that most tertiary Australian healthcare organisations would probably invest in these surgical offloading interventions, for their patients with such a serious and urgent problem (i.e. neuropathic plantar forefoot DFU) that had previously failed to heal following non-surgical offloading treatment, if they knew that these surgical procedures were probably more effective to heal those patients (1, 4). Furthermore, the panel concluded that the equipment and costs required to undertake such procedures is likely available in most hospitals that have an orthopaedic surgeon available. However, the panel did suggest there may be personnel and expertise barriers to performing some of these surgical procedures. Whereas most lower limb surgeons would have the necessary skills to perform ATL, GR, single MTH resection and joint arthroplasty, the panel felt that more complex ATL lengthening/balancing and multiple MTH resection or joint arthroplasty surgery may only be able to be undertaken by specialised foot and ankle surgeons. The panel concluded that whilst the main barrier to delivering these procedures is likely a lack of training, the NADC Australian High Risk Foot Service accreditation standards now includes as a core service indicator that "patients of HRFS should have access to an Orthopaedic surgeon with expertise in foot corrective surgery" (6) and as such this training and in turn procedures should become much more readily available in Australia. |

Note: +, agreed with IWGDF rating; ? unsure if agreed with IWGDF rating; -, disagreed with IWGDF rating.

DFU; Diabetes-related foot ulcer; EtD: Evidence to decision; IWGDF:, International Working Group on the Diabetic Foot; TCC: Total contact cast

#### **Recommendation 6B**

#### If the best recommended offloading device option fails to heal a person with diabetes and a neuropathic plantar or apical ulcer on a non-rigid toe, consider using digital flexor tenotomy to promote healing of the ulcer (Weak; Low).

**eTable A8:** Detailed justification for Recommendation 6B

| EtD criteria | Rating  (agreed*) | Detailed justifications |
| --- | --- | --- |
| Problem a priority | Yes  (+) | The panel agreed with the IWGDF that the available evidence supported that neuropathic plantar or apex digital ulcer that had failed to heal using non-surgical offloading interventions are probably a serious and urgent health care problem to treat internationally (1) and also in Australia (2). |
| Value of outcomes | Probably no important uncertainty  (+) | The panel also agreed with the IWGDF that there was probably no important uncertainty in that most Australian patients (and providers) would place most critical value on the outcome of healing their ulcer over other important outcomes (1). |
| Desirable effects | Moderate  (+) | The panel agreed with the IWGDF that for digital flexor tenotomy surgical offloading procedures that there were moderate desirable effects to heal plantar or apex ulcers on a non-rigid toe that had failed to heal with non-surgical offloading interventions. This was based on the findings of two systematic reviews, that included up to eight non-controlled case-series studies, showing an overall mean time-to-healing of 30 days (4, 44, 45), plus, one recent Australian case-series study published since the IWGDF systematic review that found a mean time-to-healing of 10 days (46). Although the healing times are impressive compared to historical data as all findings were from case series and there have been no controlled trials directly comparing this procedure with a non-surgical offloading control, the panel felt caution was required on these positive healing outcomes reported (1, 4, 44). Further, all studies identified only included participants with digital flexion deformity or non-rigid toe (44, 45) and thus, there was no evidence for this procedure in participants with rigid deformities. Therefore, we assessed these digital flexor tenotomy surgical offloading procedures had moderate desirable effects on healing neuropathic plantar or apex digital ulcers on a non-rigid toe. |
| Undesirable effects | Small  (+) | The panel agreed with the IWGDF that digital flexor tenotomy surgical offloading procedures had small undesirable effects. This was based on these case series reporting seemingly similar low levels (2-16%) of adverse events to that of historical non-surgical offloading interventions, except that 5-16% of participants reported a transfer ulcer adverse event which is a relatively rare adverse event in non-surgical offloading interventions (1, 4, 44). Therefore, in recognition of the small increased risk of new transfer ulcers developing after the procedure, the panel assessed these digital flexor tenotomy surgical offloading procedures had small undesirable effects. |
| Balance of effects | Probably favours the intervention(s) (+) | The panel agreed with the IWGDF that the difference in balance of effects between the (moderate) desirable and (small) undesirable effects probably favoured the digital flexor tenotomy procedures over other non-surgical offloading interventions (typically devices or footwear) for healing neuropathic plantar or apical digital ulcers in those with non-rigid toes that had failed to previously heal using other non-surgical offloading interventions alone (1). |
| Quality of evidence | Low  (+) | The panel agreed with the IWGDF that the collective quality of supporting evidence was low (1). This was based on only having evidence from non-controlled case series, but that this evidence was consistent and supported by recent Australian case series (1, 4, 46). Thus, on balance we also had low confidence in the certainty of the findings and rated the quality of evidence as low. |
| Acceptability | Probably yes  (+) | The panel agreed with the IWGDF that using these surgical offloading procedure interventions would probably be acceptable to most patients (and providers) that had previously failed to heal their plantar or apex digital ulcers using non-surgical offloading interventions in most of the healthcare organisations that typically provide such treatment (1). This was based on the only study reporting patient satisfaction, being that of the recent Australian case series, that reported all participants were satisfied with the outcome of the procedure (4, 46). Furthermore, the panel felt that most Australian patients (and providers) would probably accept the evidence that the balance of effects would be in favour of these surgical offloading procedures in non-healing circumstances, that they are unlikely to affect the patient’s autonomy after the immediate post-operative period, and that no patients are likely to disapprove morally. Thus, we concluded these surgical procedures are probably acceptable to most Australian patients (and providers) in these circumstances (4). |
| Feasibility | Yes  (?) | The panel was unsure if they agreed with the IWGDF as it was unclear as to what the IWGDF feasibility rating was for this recommendation. However, we suggest that using these digital flexor tenotomy surgical offloading procedures would be feasible in most healthcare organisations that patients attended for such treatment in Australia. This was based on the panel concluding that most tertiary Australian healthcare organisations would probably invest in these relatively simple surgical offloading procedure (1), for their patients with a probably serious and urgent problem (i.e. neuropathic plantar digital DFU) that had previously failed to resolve following non-surgical offloading treatment, if they knew that these procedures were simple, could easily be performed in outpatient setting and are probably more effective to heal those patients (1, 4). Furthermore, the panel concluded that the equipment and costs required to undertake such procedures should be available in most organisations, the procedure can be performed by a range of different surgeons or trained other health professionals, and that the NADC Australian High Risk Foot Service accreditation standards includes as a core service indicator that "patients of HRFS should have access to an Orthopaedic surgeon with expertise in foot corrective surgery" (6). Thus, these procedures should be therefore readily available in Australia. |

Note: +, agreed with IWGDF rating; ? unsure if agreed with IWGDF rating; -, disagreed with IWGDF rating.

DFU; Diabetes-related foot ulcer; EtD: Evidence to decision; IWGDF:, International Working Group on the Diabetic Foot; TCC: Total contact cast

## OTHER ULCER TYPES AND LOCATIONS

#### **Recommendation 9**

In a person with diabetes and a non-plantar foot ulcer, use a removable offloading device, medical grade footwear, felted foam, toe spacers or orthoses, depending on the type and location of the foot ulcer, rather than no offloading intervention to promote healing of the ulcer and to prevent further ulceration (Strong; Very Low).

**eTable A9:** Detailed justification for Recommendation 9

| EtD criteria | Rating  (agreed*) | Detailed justifications |
| --- | --- | --- |
| Problem a priority | Yes  (+) | The panel agreed with the IWGDF that the available evidence supported that non-plantar DFU are both a serious and urgent health care problem to treat internationally (1) and also in Australia. |
| Value of outcomes | Probably no important uncertainty  (+) | The panel also agreed with the IWGDF that there was probably no important uncertainty in that most patients (and providers) would place most critical value on the outcome of healing their ulcer in Australia over other important outcomes (1). However, the panel were uncertain regarding surrogate and secondary outcomes given that the burden from non-plantar ulcer remains understudied and poorly understood and as the role of pressure offloading in relation to non-plantar ulcers remains unclear and the implications of using offloading interventions on patient satisfaction, cost-effectiveness and adverse events and activity levels is unknown given current limited evidence (4). |
| Desirable effects | Don’t know, but likely to be moderate  (-) | Contrary to the IWGDF, and given that the most recent systematic review on the topic found that overall, there was very little evidence available on how to best offload non-plantar foot ulcers (4), the panel felt that there was insufficient evidence to ascertain the effects of offloading interventions for non-plantar DFU contrary to the IWGDF. Based on only one large high‐quality RCT (47) which compared a custom‐made, fibreglass heel cast in addition to non-standardised usual care with usual care alone in 72% of the cohort with non-plantar DFU with no reported differences in DFU healing, adverse events, or patient preferences in the overall cohort, but at a higher cost, the panel were unable to make a decision regarding the balance of effects based on this single study with no reported outcomes for people with non-plantar DFU. However, the panel agreed with the IWGDF that based on expert opinion the use of offloading modalities for non-plantar DFU is likely to have more desirable effects compared to undesirable effects and therefore the strength of recommendation to use these devices should be strong. |
| Undesirable effects | Don’t know, but likely to be trivial  (-) | The panel disagreed with the IWGDF, in that based on the current available evidence regarding undesirable effects, it is unclear whether offloading interventions have an anticipated effect (difference) for the critical primary outcomes (e.g., healing) and secondary and surrogate outcomes for which there is an undesirable effect (1). The panel decided that, as the reported adverse outcomes in the single study included new DFU (47), more evidence is needed to further evaluate undesirable effects in people with non-plantar DFU (1). |
| Balance of effects | Favours the intervention(s)  (+) | The panel agreed with the IWGDF that the difference in balance of effects probably favours using offloading interventions to heal non-plantar DFU compared to no offloading intervention. This was however purely based on expert opinion only, given that it is unclear at this time whether the balance of effects favours any one intervention over the other for the treatment of non-plantar DFUs (1). |
| Quality of evidence | Very low  (-) | The panel disagreed with the quality of evidence reported by the IWGDF as a “low” quality of evidence and downgraded this to a “very low” level of evidence. The panel agreed that given there is only one heterogenous clinical trial on this topic and given that the current recommendation refers to several types of offloading interventions, that until further evidence becomes available, the quality of evidence should remain “very low”. |
| Acceptability | Probably yes  (+) | The panel agreed with the IWGDF on the overall acceptability for using an offloading device by most patients (and providers) in most healthcare organisations that typically provide such treatment for people with non-plantar DFU. The panel agreed with the IWGDF that despite a lack of evidence to support acceptability of offloading interventions for treating non-plantar DFU, the expert opinion is a person with diabetes and a non-plantar foot ulcer should be advised against wearing regular footwear and be fitted with an offloading device (or use a device) that can be worn safely and without harm while achieving a reduction in trauma to the ulcer site. The panel agreed that users will need to be instructed on safe use and how to monitor for any adverse effects. In addition, the panel felt that the offloading device should not cause any excessive plantar pressures which will lead to a higher risk of plantar ulcers. Therefore, similar to the IWGDF, the panel agrees that offloading modalities compared with standard wound care alone would produce benefits in terms of DFU healing, mechanical stress reduction, and patient preference that should outweigh any harms or small costs of treatment (1). |
| Feasibility | Probably yes  (+) | The panel also agreed with the IWGDF that the recommended offloading devices is likely to be less of a barrier compared to the availability of custom-made therapeutic footwear within Australian sites (1). The panel agreed that the recommended interventions are sustainable and that there are probably no important barriers that are likely to limit the feasibility of implementing the intervention or require consideration when implementing it. |

Note: +, agreed with IWGDF rating; ? unsure if agreed with IWGDF rating; -, disagreed with IWGDF rating.

DFU; Diabetes-related foot ulcer; EtD: Evidence to decision; IWGDF:, International Working Group on the Diabetic Foot; TCC: Total contact cast

**APPENDIX B: Tables of detailed justifications for recommendations**

## OFFLOADING DEVICES

#### **Recommendation 1A**

In a person with diabetes and a neuropathic plantar forefoot or midfoot ulcer, use a non-removable knee-high offloading device over a removable offloading device to promote healing of the ulcer (GRADE strength of recommendation: Strong; Quality of evidence: Moderate).

**eTable B1:** Detailed considerations for Recommendation 1A

| Topic | Considerations |
| --- | --- |
| General implementation | The panel agreed with the IWGDF that there are two main types of non-removable knee-high offloading devices: total contact casts (TCCs) and non-removable walkers (often termed “instant TCCs”). TCCs are custom-made, knee-high, non-removable casts that can be applied using several different methods and materials. Non-removable walkers are prefabricated, knee-high, removable cast walkers, such as CAM walkers, moonboots or air cast walkers, that are made irremovable by wrapping a layer of fibreglass, plaster of paris, cohesive bandage, or tie wrap around the device to make it non-removable to the patient (1). In terms of choosing which of these two types should be used we refer health professionals to recommendation 1B which addresses that very question.  Firstly, we suggest when considering non-removable offloading devices, that health professionals consider providing the patient with locally developed patient-friendly written information on the likely benefits, risks, contraindications and 24-hour emergency contact information for each device based on the desirable and undesirable effects, contraindications outlined and the personal circumstances that may impact on wearing these devices such as job requirements, frequent driving, hot climates, or infrequent ability to attend follow-up care. We suggest such information, when discussed with the patient, will enable patients to make a fully informed decision on the offloading device they consider is best for them after weighing up the likely anticipated benefits (e.g. healing) and risks (e.g. adverse events) (1, 48) in the context of their personal circumstances. For example, according to the available evidence, we could be moderately confident that a patient wearing a non-removable knee-high offloading device could anticipate to be ~20-40% more likely to heal after 3 months of offloading treatment, and 1-2 weeks quicker, with similar relatively low chance of an adverse events (0-20%) and satisfaction, than if they chose to wear a removable knee-high offloading device instead. Yet, it may also mean they may not be able to drive and find bathing and other hygiene practices more challenging than if in a removable knee-high offloading device (5).  The panel agreed with the IWGDF that whilst no trials defined the insoles (or as defined by the IWGDF as “foot-device interface” and also often termed orthoses) that were used in these devices, that custom-made or prefabricated pressure offloading insoles should be incorporated within these devices to further reduce peak pressures at the ulcer site (1, 48). The panel suggests for such an insole using EVA customised to the patient’s foot (and sometimes lower leg for a total contact insole) shape in combination with softer cushioning material covers such as Poron^TM^ (which possesses the features of cushioning and memory) and/or Plastizote (which will accommodate and mould to bony prominences) (28, 49). Additionally, the panel agreed with the IWGDF suggestion that a shoe raise (sometimes referred to as an “evenup”) on the contralateral shoe should be considered to reduce any acquired limb length discrepancy and potentially prevent subsequent contralateral ulcers or knee or hip biomechanical complaints in patients (1, 48). If available, the panel also suggests using validated plantar pressure measurements to objectively measure and guide the modification of the insole, evenup and device to further optimise the reduction of plantar pressure at the DFU site (1, 48). Otherwise, consideration should be given to advising patients to minimise their weight-bearing activity to that that is essential and using additional walking aid, such as walking frames, to support patients to safely optimise plantar pressure reduction. Finally, we suggest patients are reviewed <4 days of initial device use, and at least weekly after that, to monitor for adverse events and ensure optimal pressure reduction. |
| Contraindicate sub-groups | The panel agreed with the IWGDF that there are likely to be other subgroups of people with neuropathic plantar forefoot or midfoot DFUs that may be contraindicated for using non-removable knee-high offloading devices, including those with both mild infection and mild ischaemia, moderate-to-severe infection, or moderate-to-severe ischaemia (1). See accompanying infection and PAD guidelines for definitions (REFS). For these specific subgroups we refer health professionals to recommendations 7A, 7B and 7C. The panel also considers, that although the available evidence that these devices may cause falls is limited and equivocal (1), that for those at high risk of falls, that health professionals should conduct or refer for a formal falls risk assessment prior to prescribing offloading. See Australian falls guidelines for definitions, risk assessments and care (50). For those formally assessed as having a high falls risk, we suggest health professionals perhaps instead consider using removable ankle-high offloading devices that have less impact on balance and stability and refer health professionals to consider using Recommendation 3 as the balance of effects is likely to not be in favour of using irremovable offloading devices in this population. |
| Geographically remote people | The panel suggests that when considering the use of non-removable knee-high offloading devices in people from areas outside of metropolitan or large regional centres i.e. in outer regional or remote areas (51), health professionals should include in their above discussion of the benefits and risks, the potential further risks of infrequent accessibility to DFU care, hot climates and dry and dusty environments. If health professionals and their patient conclude that the patient is unable to routinely receive the weekly removal of these devices that is typically required to appropriately monitor and care for their DFU, then we suggest that the balance of effects may swing back towards using removable offloading devices due to the increased potential risk of adverse events when devices are unable to be removed for longer periods (5). Other scenarios including prolonged use of a motor vehicle, hot climates that may precipitate excessive perspiration and potential maceration to the leg and foot, or dry and dusty environments where patients may need to have the ability to more frequently remove their device to clean and check for any abrasions from foreign objects may also swing the balance of effects towards removable offloading devices. Of importance is that most clinical trials evaluating these devices were performed in temperate climates (4). However, again we strongly recommend health professionals discuss with the patient the above evidenced desirable effects (moderate benefits on healing), undesirable effects (low proportion of adverse events), alongside the patient’s personal living circumstances and our expert opinion that such undesirable effects may increase in some geographically remote circumstances to enable the patient to make a fully informed decision. |
| Aboriginal & Torres Strait Islander people | The panel also considers that non-removable knee-high offloading devices may not always be strongly recommended for Aboriginal and Torres Strait Islander people. Similar to those people living in geographically remote areas, Aboriginal and Torres Strait Islander people may also have infrequent access to care, and content with hot temperatures and dry, dusty environments. This may swing the balance of effects toward instead using a removable knee-high offloading device that has the same functional benefits on plantar pressure reduction when worn all the time but is removable for the person to more frequently check and clean their foot and leg. Additionally, Aboriginal and Torres Strait Islander people may benefit from knowing they have the freedom to more frequently remove their footwear (and offloading devices) to participate in traditional gatherings and cultural obligations. Health professionals should be sensitive to the possibility that Aboriginal and Torres Strait Islander people may feel a sense of ‘shame’ in having to wear such an obvious device that highlights they have a health issue. Thus, the panel suggests for Aboriginal and Torres Strait Islander people it is very important to carefully discuss with the person the above anticipated benefits and risks of such devices in the context of the patient’s personal circumstances. We strongly suggest that such discussions be performed in collaboration with local Aboriginal and Torres Strait Islander health care workers to optimise understanding of these devices and the requirements, such as length of time the device would need to be worn and in turn the patient’s foot health outcome. We also suggest that health professionals could consider facilitating improved culturally appropriate aesthetic appeal for such devices, such as working with local Aboriginal and Torres Strait Islander communities to come up with solutions that may improve acceptability, for example having local artwork to be applied to the device which may help decrease any shame felt by the patient. Finally, we suggest health professionals consider facilitating patients to access culturally appropriate DFU care and offloading devices, such as via Aboriginal Community Controlled Health Services, additional Aboriginal Medical Benefit Scheme entitlements for podiatry care and offloading devices, liaising with local Aboriginal and Torres Strait Islander Health Care Worker(s) to produce other culturally-appropriate resources etc (52) |
| Monitoring & evaluation | The panel agreed with the IWGDF that offloading interventions are arguably the most important intervention for healing plantar DFU and that non-removable knee-high offloading devices have the best evidence of all offloading interventions to most effectively heal DFU (1). Therefore, like the IWGDF, the panel suggests organisations need to include in their formal monitoring of key clinical performance indicators an item to objectively measure the proportion of their patients with plantar DFU (that are not contraindicated) that are prescribed non-removable knee-high offloading devices (1). We refer the reader to existing national and state based High Risk Foot Service database monitoring systems and datasets that typically include such measures of non-removable knee-high offloading devices and are usually available to the majority of Australian organisations to utilise (6, 53, 54). |
| Future research priorities | The panel also agreed with the IWGDF that there are several potential future research priority considerations that if investigated would further help patients and providers better understand the key benefits and risks of using non-removable knee-high offloading devices to heal people with plantar DFU. These include future trials on different methods or makes of custom-made and pre-fabricated non-removable knee-high offloading devices to determine which are most (cost-) effective on healing, plantar pressure reduction, weight-bearing activity reduction, adverse events, patient satisfaction and even adherence (1, 4) across a diverse group of patients. Future trials also provide greater participant numbers and the opportunity for sub-group analyses to determine patient (and foot) characteristics that benefit most from these specific devices, and important data on which participant sub-groups were excluded or declined participation for which devices (3, 55) . Furthermore, to our knowledge, whilst some non-controlled studies have investigated non-removable knee-high offloading devices in Australia, no controlled trials have been performed here. Thus, the panel encourages future Australian trials of these devices, and particularly in Aboriginal and Torres Strait Islander populations and/or regions that are either geographically remote, have hot climates or dry environments, to determine if the effects found on healing in predominantly European and US trials are also found in Australia. |

DFU; Diabetes-related foot ulcer; EtD: Evidence to decision; IWGDF:, International Working Group on the Diabetic Foot; TCC: Total contact cast

#### **Recommendation 1B**

When using a non-removable knee-high offloading device to heal a neuropathic plantar forefoot or midfoot ulcer in a person with diabetes, consider using either a total contact cast or nonremovable knee-high walker, with the choice dependent on the local resources and technical skills available, and the person’s preference and extent of foot deformity (Weak; Low).

**eTable B2:** Detailed considerations for Recommendation 1A

| Topic | Considerations |
| --- | --- |
| General implementation | The panel agreed with the IWGDF that either type of non-removable knee-high offloading devices should be the gold standard for offloading interventions for plantar forefoot and midfoot DFU (1). We also agree with the IWGDF that the selection of either a TCC or non-removable knee-high walker (also known as an instant TCC (iTCC)) should be guided by the patient’s circumstances and their treating organisation’s available resources (1). We would also highlight that the ability of the patient’s foot (and any foot deformities) to be comfortably accommodated in a cast walker should be a priority consideration when discussing with patients the benefits and risks of either device (1, 5). In cases where a patient’s foot deformities are unable, or if unsure, to be accommodated in a walker, we suggest that the risks of using a non-removable walker on potential future adverse events increases substantially and sways the balance of effects to using a TCC in these circumstances (5). However, if the patient’s foot deformity is able to be comfortably accommodated in a cast walker, then perhaps the balance of effects may swing back in favour of a non-removable knee-high walker due to the non-removable walkers being typically of lighter weight, being quicker to apply, needing much less expertise to apply and being more cost-effective (1, 4, 5). Finally, we agree with the IWGDF that there is no standard method for manufacturing a TCC or non-removable walker (1) and instead refer the reader to these guides for them to choose at their discretion based on the above considerations (56-58). Otherwise we refer the reader to the same implementation considerations outlined in Recommendation 1A to consider in these instances as well, such as optimising the pressure offloading insole, using a shoe raise or similar on the contralateral shoe and providing patient-friendly written information on the benefits and risks of the devices to enable the patient to make an informed decision based on weighing up the benefits and risks within the prism of their personal circumstances. |
| Contraindicated sub-groups | Similarly, to Recommendation 1A, the panel also suggests that those patients with various levels of infection or ischaemia should use recommendations 7A, 7B and 7D. Furthermore, the panel also suggests health professionals should consider those assessed as having a high falls risk to perhaps instead consider using Recommendation 3. Lastly, the panel suggests for those patients with a large foot deformity which cannot be comfortably accommodated in a prefabricated cast walker, that a TCC is used, such as a very wide foot, plantigrade foot, large Charcot foot deformity or extensive bunion (5). |
| Geographically remote people | Similarly to Recommendation 1A, the panel also suggests that when considering the use of either type of non-removable knee-high offloading device in people from geographically remote areas, health professionals should include in their explanation of the benefits and risks, the potential further risks of infrequent accessibility to DFU care (5), hot climates, dry and dusty environments. Please see Recommendation 1A for more details on those implementation considerations. In addition, we would highlight that if the patient has made the informed decision to use one of the types of non-removable knee-high offloading devices, then non-removable knee-high walkers which typically require less expertise, time and resources and can be more easily removed if required may be favourable in those from geographically remote areas (5). Furthermore, we suggest the health professional consider being flexible in terms of how to make the device non-removable, either via using a tie wrap, wrapping a layer of cohesive bandage (“coban”), fibreglass or plaster of paris around the cast (1, 5). A cohesive bandage for example enables patients to be able to more simply remove the cast with a pair of scissors if necessary and provides a good indication for the health professional if the cast has been removed between treatment and thus if adherence may have been impacted for the treatment period concerned (5). However, again we strongly recommend health professionals discuss with the patient concerned the above evidenced benefits and risks as outlined in Recommendation 1A, alongside the patient’s personal living circumstances and our expert opinion that such undesirable effects may increase in some geographically remote circumstances, to enable the patient to make an informed decision. |
| Aboriginal & Torres Strait Islander people | Again, similar to people from geographically remote populations, the panel suggests when considering which type of non-removable knee-high offloading device to use, health professionals should include in their considerations if the Aboriginal and Torres Strait Island person they are caring for has infrequent access to care, or lives in hot, dry or dusty environments. Additionally, as per Recommendation 1A, Aboriginal and Torres Strait Islander people may also require an ability to more frequently remove their footwear (and offloading devices) to participate in traditional gatherings and meetings. Cultural obligations may also require Aboriginal and Torres Strait Islander people to travel to other locations and communities. Thus, similarly to those in geographically remote areas that can be accommodated in a pre-fabricated knee-high walker, the panel suggests considering using a coban wrap to make the cast walker non-removable, but in turn practically removable by the patient which provides the flexibility and freedom for the patient to remove if needed and the health professional the knowledge that that patient has removed and reduced their adherence to the device over the preceding treatment period. Regardless, the panel again suggests that health professionals explain and discuss with Aboriginal and Torres Strait Islander people the benefits and risks of such devices in the context of their personal circumstances, and ideally with local Aboriginal and Torres Strait Islander health care workers to optimise understanding. |
| Monitoring & evaluation | The panel refers the reader to the same monitoring considerations contained in Recommendation 1A for this recommendation as well. Additionally the panel suggests that organisations that do monitor the proportion of eligible (not contraindicate) patients that use a non-removable knee-high offloading device, that they also consider adding options within this key clinical performance indicator to identify which type of these devices are used to help monitor patient preferences, resource utilisation and the healing outcomes of these different types of non-removable knee-high offloading devices in their local organisation. |
| Future research priorities | Again, the panel suggests the same future research considerations outlined in Recommendation 1A are also applicable to this recommendation, including future Australian trials on different methods or makes of custom-made and pre-fabricated non-removable knee-high offloading devices to determine which are most (cost-) effective on healing, plantar pressure reduction, weight-bearing activity reduction, adverse events, patient satisfaction and even adherence (1, 4), and specifically also within geographically remote and Aboriginal and Torres Strait Islander populations. |

DFU; Diabetes-related foot ulcer; EtD: Evidence to decision; IWGDF:, International Working Group on the Diabetic Foot; TCC: Total contact cast

#### **Recommendation 2**

In a person with diabetes and a neuropathic plantar forefoot or midfoot ulcer, when non-removable knee-high offloading devices are contraindicated or not tolerated, consider using a removable knee-high offloading device (and explain the importance of using) during all weight-bearing activities rather than a removable ankle-high offloading device to reduce plantar pressure and promote healing of the ulcer (Weak; Low).

**eTable B3:** Detailed considerations for Recommendation 2

| Topic | Considerations |
| --- | --- |
| General implementation | The panel agreed with the IWGDF that a removable knee‐high offloading device may be a solution for circumstances when a gold standard nonremovable knee‐high offloading device is contraindicated or cannot be tolerated by the patient (1). Intolerance can include refusal to wear a non-removable device or where the patient's circumstances do not support its use, such as due to the requirements of the patient's job (1). The panel felt that for the Australian context, patients who can safely wear a removable knee-high device should be strongly advised to wear the device consistently with explanation to the patient that wearing such a device 100% of the time potentially provides the equivalent effectiveness to using the gold standard non-removable knee-high offloading device. However, if they are unwilling or unable to safely wear the device for the majority of time that they are weight-bearing, the alternate option of a removable ankle high device may be a better option as low quality evidence has been shown this device may improve adherence to offloading treatment. Informed patient consent should be obtained for using either device after explaining to the patient that a removable knee-high offloading device is likely to be more effective at reducing plantar pressures and limiting activity, thereby reducing plantar tissue stress which could impede ulcer healing (4, 59). Otherwise, we refer the reader to the same implementation considerations outlined in Recommendation 1A to consider in these instances as well, such as optimising the pressure offloading insole, using an evenup or similar on the contralateral shoe and providing patient-friendly written information on the benefits and risks of the devices to enable the patient to make an informed decision based on weighing up the benefits and risks within the prism of their personal circumstances. Lastly, the panel emphasises the need to carefully review the specific offloading requite of the individual over time to determine their adherence levels to wearing such devices and potentially change removable devices accordingly if their adherence is being significantly impacted. |
| Contraindicated sub-groups | Similarly, to Recommendation 1A, the panel also suggests that those patients with various levels of infection or ischaemia should instead consider using recommendations 7a, 7b and 7c. Furthermore, the panel also suggests health professionals should consider those assessed as having a high falls risk may be more suited to removable ankle-high devices compared to removable knee-high devices. |
| Geographically remote people | In addition to the above implementation considerations, the panel suggests for geographically remote people in hot climates that may precipitate excessive perspiration and potential skin maceration, that this may also swing the balance of effects towards removable ankle-high offloading devices. However, that would be in exceptional circumstances as these devices are both removable. However, again we strongly recommend health professionals discuss with the patient concerned the above evidenced benefits and risks as outlined in Recommendation 2, alongside the patient’s personal living |
| Aboriginal & Torres Strait Islander people | Similar to considerations in Recommendations 1, the panel suggests when considering which type of removable offloading device to use, that health professionals should be sensitive to the possibility that Aboriginal and Torres Strait Islander people may feel a sense of ‘shame’ in having to wear such a device that highlights they have a health issue. Thus, the panel suggests for Aboriginal and Torres Strait Islander people it is very important to carefully discuss with the person the above anticipated benefits and risks of such devices in the context of the patient’s personal circumstances and if the person considers they are more likely to wear the ankle-high device as it reduces their ‘shame’ then the panel suggests that would be the best option compared with a removable knee-high device in this instance. Again, we suggest that such discussions should be performed in collaboration with local Aboriginal and Torres Strait Islander health care workers to optimise understanding and in turn the patients foot health outcome. |
| Monitoring & evaluation | The panel refers the reader to the same monitoring considerations contained in Recommendation 1A for this recommendation as well. In addition, the panel strongly suggest that where possible, clinicians should monitor the efficacy of an offloading device using in-device plantar pressure analysis to ensure efficacy of the device being used in lowering plantar pressures or if plantar pressure analysis is not available observe for skin changes such as callus build-up, indicating offloading may not be effective (59). Additionally, the panel suggests that organisations that do monitor the proportion of patients that use removable knee-high offloading device, that they also consider adding options within this key clinical performance indicator to identify which type of these devices are used to help monitor patient preferences, resource utilisation and the healing outcomes of these different types of devices in their local organisation. |
| Future research priorities | The panel suggests the same future research considerations outlined in Recommendation 1A are also applicable to this recommendation, including future Australian trials on different methods or makes of custom-made and pre-fabricated removable offloading devices to determine which are most (cost-) effective on healing, plantar pressure reduction, weight-bearing activity reduction, adverse events, patient satisfaction and adherence (1, 4), and specifically also within geographically remote and Aboriginal and Torres Strait Islander populations. In particular, whether removable knee-high devices have a superiority in primary outcome compared with removable ankle-high devices with respect to ulcer healing remains unknown and should be tested in large well powered and designed clinical trials. Lastly, studies evaluating behaviour modification and motivational interviewing approaches to increase adherence to offloading treatments should be a major focus of future research. This includes studies involving Aboriginal and Torres Strait Islander peoples. |

DFU; Diabetes-related foot ulcer; EtD: Evidence to decision; IWGDF:, International Working Group on the Diabetic Foot; TCC: Total contact cast

#### **Recommendation 3**

In a person with diabetes and a neuropathic plantar forefoot or midfoot ulcer, when knee-high offloading devices are contraindicated or not tolerated, use a removable ankle-high offloading device (and explain the importance of using) during all weight-bearing activities rather than medical grade footwear to promote healing of the ulcer (Strong; Very low)

**eTable B4:** Detailed considerations for Recommendation 3

| Topic | Considerations |
| --- | --- |
| General implementation | The panel agreed with the IWGDF that a removable ankle‐high offloading device may be a solution for circumstances when a knee‐high offloading device is contraindicated or cannot be tolerated by the patient or is unavailable. Intolerance by the patient can include refusal to wear the device or the patient's circumstances do not support its use, such as unable to use the device as part of the patient's job (1). A removable ankle-high device is likely to be more effective at reducing plantar pressures and heal DFU than medical grade footwear based on a very low level of evidence thereby reducing tissue stress which could impede ulcer healing (4, 59). Otherwise, we refer the reader to the same implementation considerations outlined in Recommendation 1A to consider in these instances as well, such as optimising the pressure offloading insole, using an evenup or similar on the contralateral shoe and providing patient-friendly written information on the benefits and risks of the devices to enable the patient to make an informed decision based on weighing up the benefits and risks within the prism of their personal circumstances. |
| Contraindicated sub-groups | Similarly, to Recommendation 1A, the panel also suggests that those patients with various levels of infection or ischaemia should instead consider using recommendations 7A, 7B and 7C. Furthermore, the panel also suggests health professionals should consider those assessed as having a high falls risk may be more suited to certain types of removable ankle-high devices. The caveats regarding the use of specific types of ankle-high offloading devices which may exacerbate gait abnormalities and increase falls risk such as the half-shoe needs to be evaluated on a case by case basis within older populations given risk of falls and instability during gait (60). |
| Geographically remote people | Please see Recommendation 1A for more details on those implementation considerations for geographically remote people that could be considered for this recommendation as well. In addition, we would highlight that removable devices may be more favourable in those from geographically remote areas (5). Furthermore, we suggest that health professionals should specifically address adherence to using the device with the user (48, 61). However, again we strongly recommend health professionals discuss with the patient concerned the above evidenced benefits and risks as outlined in Recommendation 3, alongside the patient’s personal living circumstances and our expert opinion. |
| Aboriginal & Torres Strait Islander people | The panel considers the same considerations as in Recommendation 1A for Aboriginal and Torres Strait Islander people and those general considerations outlined above apply for this recommendation as well. |
| Monitoring & evaluation | The panel refers the reader to the same monitoring considerations contained in Recommendation 1A for this recommendation as well. Additionally, the panel suggests that organisations consider adding options within their key clinical performance indicator to identify which type of these devices are used to help monitor patient preferences, resource utilisation and the healing outcomes of these different types of offloading devices in their local organisation. |
| Future research priorities | The panel suggests the same future research considerations outlined in Recommendation 1A are also applicable to this recommendation (1, 4), and specifically also within geographically remote and Aboriginal and Torres Strait Islander populations. Other key areas for future research includes (1) research relating to the use of insole/interface or padding within ankle-high offloading devices (2) research evaluating features to minimise shear including rocker sole designs for specific devices and 3) research to verify the effectiveness of pressure offloading at the region of interest. Lastly, studies evaluating behaviour modification and motivational interviewing approaches to increase adherence to offloading treatments should be a major focus of future research. This includes studies involving Aboriginal and Torres Strait Islander peoples. |

DFU; Diabetes-related foot ulcer; EtD: Evidence to decision; IWGDF:, International Working Group on the Diabetic Foot; TCC: Total contact cast

## FOOTWEAR

#### **Recommendation 4**

In a person with diabetes and a neuropathic plantar forefoot or midfoot ulcer, when ankle-high offloading devices are contraindicated or not tolerated, use medical grade footwear rather than other footwear types or no footwear to promote healing of the ulcer (Strong; Low).

**eTable B5:** Detailed considerations for Recommendation 4

| Topic | Considerations |
| --- | --- |
| General implementation | We refer the reader to the same general implementation considerations outlined in Recommendation 1-3 to consider in these instances for medical grade footwear as well, such as optimising the pressure offloading insole, using an evenup or similar on the contralateral shoe and providing patient-friendly written information on the benefits and risks of the devices and footwear to enable the patient to make an informed decision based on weighing up the benefits and risks within the prism of their personal circumstances. As per the other recommendations, health professionals should consider providing to each patient the national clinical pathway provided (Figure 1) and locally developed patient-friendly written information on the likely benefits, risks and 24-hour emergency contact information for each device or footwear (based on the desirable and undesirable) stated in earlier recommendations (1, 48). |
| Contraindicated sub-groups | We are unaware of any significant sub-groups who may be contraindicated to correctly fitted medical grade footwear (32). However, a contraindication for prefabricated medical grade footwear are those with a large foot deformity(s) that cannot be safely accommodated in prefabricated medical grade footwear, such as a very wide foot, plantigrade foot, a large Charcot foot, or extensive bunion (20, 32). We strongly suggest using custom-made medical grade footwear instead for these people. |
| Geographically remote people | Similarly to Recommendation 3, the panel suggests that when considering the use of any type of offloading device or footwear in people from geographically remote areas, health professionals should include in their explanation of the benefits and risks, the potential further risks of infrequent accessibility to DFU care (5), this includes hot climates and dry and dusty environments and geographical distance for travel. Please see Recommendation 1A for more details on those implementation considerations for geographically remote people that should be considered for this recommendation as well. Furthermore, we suggest that health professionals should specifically address adherence to using the device with the user (48, 61). However, again we strongly recommend health professionals discuss with the patient concerned the above evidenced benefits and risks as outlined in Recommendation 4a, alongside the patient’s personal living circumstances and our expert opinion. |
| Aboriginal & Torres Strait Islander people | Aboriginal and Torres Strait Islander peoples are known to wear flip flops, slip-on footwear or no footwear, and inappropriately fitting footwear may contribute to foot complications and contribute to poor offloading and DFU healing (62). Therefore, therapeutic footwear may be an appropriate option preferentially over no footwear or other off-the-shelf footwear for some Aboriginal and Torres Strait Islander individuals that do not find that offloading devices are culturally acceptable after discussing the benefits, risks, contra-indications and personal circumstances. The panel suggests that health professionals should include in their discussion with the Aboriginal and Torres Strait Islander person they are caring for and seek to identify whether they have infrequent access to care, hot, dry or dusty conditions in which they live. Additionally, as per Recommendation 1A and 2, Aboriginal and Torres Strait Islander people may also require an ability to more frequently remove their footwear (and offloading devices) to participate in traditional gatherings and meetings. Cultural obligations may require Aboriginal and Torres Strait Islander people to travel to other locations and communities. Therefore, therapeutic footwear may be more acceptable to some Aboriginal and Torres Strait Islander people. Thus, similarly to those in geographically remote areas that can be accommodated in a removable walker, the panel suggests considering using the same, which provides the flexibility and freedom for the patient to remove if needed and the health professional the knowledge that that patient has removed and reduced their adherence to the device over the preceding treatment period. Regardless, the panel again suggests that health professionals consider it very important to carefully explain and discuss with Aboriginal and Torres Strait Islander people the benefits and risks of such devices in the context of their personal and cultural circumstances. The panel highlights that such explanations should, where the patient is agreeable, be ideally performed in collaboration with local Aboriginal and Torres Strait Islander health care workers to optimise understanding. |
| Other sub-groups of interest | Similarly, to Recommendation 1A, the panel also suggests that those patients with various levels of infection or ischaemia should instead consider using recommendations 7a, 7b and 7c. Lastly, the panel emphasise the need to carefully review the specific offloading requirements of the individual. |
| Monitoring & evaluation | The panel refers the reader to the same monitoring considerations contained in Recommendation 2-3 for this recommendation as well. Additionally, we suggest that the use of medical grade footwear is perhaps captured and monitored in organisational monitoring systems to try and ensure that medical grade footwear to offload DFU is only used in rare circumstances. |
| Future research priorities | The panel suggests the same future research considerations outlined in Recommendation 2-3 are also applicable to this recommendation (1, 4), and specifically also within geographically remote and Aboriginal and Torres Strait Islander populations. Other key areas for future research includes Australian based studies evaluating the efficacy and acceptability of therapeutic footwear when compared to other offloading options especially where standard care is no footwear or standard footwear. |

DFU; Diabetes-related foot ulcer; EtD: Evidence to decision; IWGDF:, International Working Group on the Diabetic Foot; TCC: Total contact cast

## OTHER (NON-SURGICAL) OFFLOADING TECHNIQUES

#### **Recommendation 5**

In a person with diabetes and a neuropathic plantar forefoot or midfoot ulcer, consider using felted foam in combination with an offloading device or footwear rather than using the offloading device or footwear alone to further reduce plantar pressure and promote healing of the ulcer (Weak; Very Low).

**eTable B6:** Detailed considerations for Recommendation 5

| Topic | Considerations |
| --- | --- |
| General implementation | The panel agreed with the IWGDF that felted foam in addition to standard offloading may be beneficial in either removable or nonremovable offloading devices. Otherwise, we refer the reader to the same implementation considerations outlined in Recommendation 1A to consider in these instances as well, such as optimising the pressure offloading insole, using an evenup or similar on the contralateral shoe and providing patient-friendly written information on the benefits and risks of the devices to enable the patient to make an informed decision based on weighing up the benefits and risks within the prism of their personal circumstances. The panel also recommends that staff expertise should be assessed to apply and to monitor the use of the padding materials if they are to be used in the Australian context and appropriate education regarding how to use the felt foam needs to be provided to the patient. Some practice points could be included here; Ensure the design of the device can accommodate the felt, aim to replace felt as it compresses (within 1 week ideally) and minimise the effect of transferring load by using felt in combination with cushioning, bevelling the edge of the felt and using a large (not donut style) padding to disperse the weight-bearing load. Furthermore, when there is space to accommodate them, the use accommodative orthoses, cushioning (using materials which have demonstrated efficacy), felt deflective paddings or metatarsal paddings may augment pressure offloading according to indirect evidence. See supplementary information. |
| Contraindicated sub-groups | We agreed with IWGDF that we are unaware of any significant sub-groups who may be contraindicated to correctly felted foam (20). However, we suggest those with severe ischaemia, very fragile skin or heavily exudating ulcers are likely to be contraindicated to using felted foam that is adhered to the foot itself (20). We strongly suggest in these circumstances to instead adhere the felted foam to the pressure offloading insole in the offloading device or footwear if choosing to use felted foam. |
| Geographically remote people | The panel suggests that when considering the use of any main or supplementary offloading device in people from geographically remote areas, the use of padding may lead to an increased risk of foot infections, as it acts as a foreign body material which can harbour waste and bacteria near a wound, when applied directly to the skin. Therefore, the panel felt that caution should be exercised when using this intervention in sandy or high humidity areas and in rural and remote populations. Please see Recommendation 1A for more details on those implementation considerations for geographically remote people that should be considered for this recommendation as well. |
| Aboriginal & Torres Strait Islander people | Similarly to those in geographically remote areas, the panel suggests the same caution of using felted foam in select patients where the flexibility and freedom for the patient to remove the felted foam if needed is compromised. It may be more appropriate to apply felted foam directly to the foot or to the offloading device and this needs to be discussed with each individual Aboriginal and Torres Strait Islander person the clinician is caring for. Regardless, the panel again suggests that health professionals consider it very important to carefully explain and discuss with Aboriginal and Torres Strait Islander people the benefits and risks of such devices in the context of their personal and cultural circumstances. The panel highlights that such explanations should, where the patient is agreeable, be ideally performed in collaboration with local Aboriginal and Torres Strait Islander health care workers to optimise understanding. |
| Other sub-groups of interest | The panel acknowledge that given the use of felted foam introduces extra material to the foot-device interface which can harbour bacteria and debris, particular consideration needs to be given to this |
| Monitoring & evaluation | The same monitoring considerations as outlined in Recommendation 2 also apply. In addition, we suggest that felted foam may be considered as a secondary item offloading intervention option captured and monitored in organisation monitoring systems. |
| Future research priorities | The panel suggests further randomised controlled trials are required to adequately evaluate the benefit, acceptability and costs of felted foam within the Australian context. |

DFU; Diabetes-related foot ulcer; EtD: Evidence to decision; IWGDF:, International Working Group on the Diabetic Foot; TCC: Total contact cast

## SURGICAL OFFLOADING TECHNIQUES

#### **Recommendation 6A**

If the best recommended offloading device option fails to heal a person with diabetes and a neuropathic plantar metatarsal head ulcer, consider using Achilles tendon lengthening or Gastrocnemius recession, metatarsal head resection(s), or joint arthroplasty to promote healing of the ulcer (Weak; Low).

**eTable B7:** Detailed considerations for Recommendation 6A

| Topic | Considerations |
| --- | --- |
| General implementation | The panel agreed with the IWGDF that these surgical procedures have been historically used, and are evidenced to show some benefit, when treating patients with plantar forefoot DFUs that have failed to heal after using non-surgical offloading interventions, and that these procedures typically come with an inherent risk of complications (1). Thus, the panel strongly suggests that these procedures are only considered for implementation when the patient has failed to heal after receiving a good standard of DFU care (1, 3). We suggest a good standard of DFU care should include: evidence-based DFU classification (63), appropriate debridement (64), wound dressings (64), revascularisation considerations (65) , antimicrobial treatment if required (66), along with the best available offloading device (See Recommendations 1-4) (1, 3). We also suggest failing to heal be defined as <50% reduction in DFU size/area after receiving at least four weeks of such good standard of DFU care (1, 3, 4, 67). Thus, if the patient has failed to heal, according to the above definitions, we suggest health professionals then consider discussing these surgical offloading procedures with their patient.  Prior to any such discussions with patients, we suggest as mandatory that a best practice DFU assessment and general health is performed to determine the patient’s fitness to undergo such a surgical procedure and their current infection and ischaemia status (1, 48). We refer the reader to the accompanying Australian PAD and DFI guidelines for those assessments and status criteria (65, 66). These assessments will help to determine if the patient is contraindicated for such surgery, such as those with severe ischaemia (1, 48), and we refer the reader to the other subgroup considerations for contraindications. If the patient is not contraindicated, then we suggest health professionals discuss with their patient the potential benefits (desirable) and risks (undesirable effects) of these surgical offloading procedure options within the prism of the patient’s circumstances. We would suggest such discussions include the potential moderate benefit (desirable effect) that undergoing such a surgical offloading procedure may have on healing and plantar pressure reduction, the potential small risks (undesirable effects) that come with that for surgical complications, including discomfort, short-term gait challenges, potential for ruptured tendons, new transfer ulcers (e.g. heel ulcers for ATL procedures), plus the effect on short-term autonomy and the need to continue to wear best available offloading devices post-surgery and potentially undergo some rehabilitation (48).  Again we suggest that health professionals should consider providing each patient the national clinical pathway (Figure 1) and locally developed patient-friendly written information on the likely benefits, risks, Australian applicability and feasibility information for these surgical offloading procedures based on the detailed judgements section above and any subgroup considerations outlined below. Such information discussed with the patient should enable the patient to make a fully informed decision and provide informed consent to determine if such surgical offloading interventions are best for them in the context of their personal circumstances. Finally, we suggest using validated plantar pressure measurements if available, before and after the procedure, to objectively measure and monitor the plantar pressure reduction and potentially guide post-operative offloading device therapy (See Recommendations 1-4) (1, 48).  Lastly, we strongly suggest that any lower limb surgeon considering such offloading procedures needs to be appropriately trained and be able to demonstrate competency in the procedure concerned. In addition to the procedure itself this also includes being able to demonstrate competency in best practice DFU assessments, recognising any contraindications and the multidisciplinary care required to both pre- and post-operatively manage the patient. Such surgery is preferably performed by a specialist foot and ankle surgeon, and as such we recommend that if these surgeons aren’t available in a treating organisation, then at the very least the organisation should have a formal referral pathway to a specialist foot and ankle surgeon to advise when surgical offloading is required. |
| Contraindicated sub-groups | We agreed with IWGDF that a significant contraindication for these surgical procedures is moderate-to-severe ischaemia (20). Furthermore, we suggest other sub-groups of people are also likely to be contraindicated, include those with moderate-to-severe infection, moderate-to-severe oedema, cognitive impairment impairing capacity to provide informed consent or conditions precluding anaesthesia. Lastly, we suggest people with normal (>5 degrees of) ankle dorsiflexion are not likely to benefit from Achilles tendon lengthening or Gastrocnemius Recession procedures, and metatarsal head resections should be the surgical procedure considered instead (45). Otherwise as persons undergoing these procedures will be required to post-operatively use offloading devices, we refer the reader back to the contraindications for those offloading devices in Recommendations 1-4. |
| Geographically remote people | The panel suggests when considering using any of these surgical offloading procedures for people from geographically remote areas, that health professionals should include in their benefits and risks discussions with their patient, that the patient will mostly likely also have to travel to receive such procedures from a large tertiary organisation and potentially received post-operate DFU care from that organisation as well. Furthermore, if by small chance the patient does develop an adverse event they may also be required to return to that organisation for care as well. Otherwise, we refer the reader back to earlier considerations for geographical remote populations for those patients that require to wear different offloading devices as part of their post-operative care in Recommendations 1-4. |
| Aboriginal & Torres Strait Islander people | In a similar manner to the considerations for people in geographically remote locations, the panel also considers that for Aboriginal and Torres Strait Islander people that health professionals include in their benefits and risk discussion the potential need for people to travel away from their community, country and families to receive such procedures and post-operative care from large tertiary organisations. Thus, for this recommendation in particular we strongly recommend that this discussions with Aboriginal and Torres Strait Islander people are held in collaboration with a local Aboriginal and Torres Strait Islander health care workers to help the person optimise their understanding of the benefits and risks for their circumstances, but also to help the health professional understand the sensitivities that the Aboriginal and Torres Strait Islander persons may have about having to leave their community to receive such care, and the length of time they may be away. We suggest such discussion are also performed with family members and with adequate time to truly discuss, understand and consider these benefits and risks so as to enable the person and their family to make a truly informed decision. We are unaware of any guidelines for culturally appropriately discussing surgery with Aboriginal people, however, the panel feels the developments of such guidelines and implementation in surgical training would be very useful. |
| Other sub-groups of interest | Similarly, to earlier recommendations, the panel suggests that those patients with various levels of infection or ischaemia should instead consider using recommendations 7a, 7b and 7c. Otherwise, the panel suggests that subgroups of people who are likely to be contraindicated to these procedures are those with infection close to the operative field, medical conditions resulting in moderate-to-severe oedema (such as congestive heart failure), medical conditions that preclude anaesthesia and conditions that may result in an inability to adhere with the post-operative offloading in a CAM boot or knee high immobiliser (such as dementia). Additionally, when undertaking GR meticulous haemostasis is required to avoid haematoma formation particularly when patients are taking anticoagulant medication for medical conditions such as ischaemic heart disease."  Furthermore, the panel suggest that there is unlikely to be a benefit from ATL or GR procedures in those patients with already greater than 5 degrees of ankle dorsiflexion and would suggest against using these procedures in this patient sub-group, but instead perhaps consider using MTH resections instead (Ref Mueller 2003).  Lastly, the panel refers the reader back to the other subgroup considerations in Recommendations 1-4 for the various offloading device treatments that may be considered for the patient to use post-operatively. |
| Monitoring & evaluation | Again, like the IWGDF, the panel suggests organisations include in their formal monitoring systems options to be able to collect, monitor and analyse the impact of these different recommended surgical offloading procedures on their patients’ healing outcomes (1). Again, as per earlier recommendations, we refer the reader to existing national and state based High Risk Foot Service database monitoring systems and datasets that typically include such measures of surgical offloading procedures and are usually available to the majority of Australian organisations to employ (6, 53, 54). Furthermore, we suggest that organisations can engage their local health information managers to help them obtain such routinely collected existing monitoring data from their local hospital discharge datasets also using Australian Classification of Health Interventions codes for these specific surgical interventions (68, 69) |
| Future research priorities | The panel also agreed with the IWGDF that there are very few previous controlled trials investigating surgical offloading interventions and most have been in participants where non-surgical offloading interventions have failed. Thus, we also agree with the IWGDF that more robust RCTs are required to investigate the impact of these surgical offloading procedure interventions compared to gold standard offloading device controls in participants with complicated non-healing ulcers, but also in those that don’t have such complicated ulcers to determine if surgical procedures would provide more permanent benefits in uncomplicated ulcers. As mentioned in earlier future research priority considerations for offloading device interventions these studies should be designed according to the IWGDF recommended reporting standards for trials in people with DFU (3), including investigating different surgical offloading procedure techniques to determine which are most (cost-) effective on the multiple outcomes of healing, plantar pressure reduction, weight-bearing activity reduction, adverse events, patient satisfaction and even adherence (1, 3, 4). Again, the panel suggests such Australian trials of these procedures also include Aboriginal and Torres Strait Islander populations and/or regions that are geographically remote. Lastly, the panel suggests future research into community perceptions of the benefits and risks of such surgical procedures are undertaken, such as those in qualitative studies to truly understand the patient perspective, particularly in Aboriginal and Torres Strait Islander peoples. |

DFU; Diabetes-related foot ulcer; EtD: Evidence to decision; IWGDF: International Working Group on the Diabetic Foot; TCC: Total contact cast

#### **Recommendation 6B**

If the best recommended offloading device option fails to heal a person with diabetes and a neuropathic plantar or apical ulcer on a non-rigid toe, consider using digital flexor tenotomy to promote healing of the ulcer (Weak; Low).

**eTable B8:** Detailed considerations for Recommendation 6B

| Topic | Considerations |
| --- | --- |
| General implementation | Although this digital flexor tenotomy procedure is a less complex surgical offloading procedure, we refer the reader to similar implementation considerations outlined in Recommendation 5 in these instances as well, such as considering these procedures only in those that have failed to heal after using a good standard of DFU care, performing a best practice DFU assessment to determine any current infection or ischaemia contraindications, and gaining informed consent from the patient after thoroughly discussing the benefits (aforementioned desirable effects) and risks (aforementioned undesirable effects). Furthermore, for this recommendation we strongly suggest that the digital deformity is assessed to confirm it is a flexion deformity (or non-rigid toe) that would benefit from the procedure and if available we suggest using validated plantar pressure measurements, before and after the procedure, to objectively measure and monitor the plantar pressure reduction (1, 48). Lastly, we strongly suggest that any health professional considering undertaking this surgical offloading procedure needs to be appropriately trained and be able to demonstrate competency in using this procedure. |
| Contraindicated sub-groups | The same contraindications as in Recommendation 6A apply. In addition, we suggest people with a rigid toe deformity are unlikely to benefit from these procedures. |
| Geographically remote people | The panel suggests in addition to those general implementation considerations above, that health professionals in geographically remote areas include in their benefits and risks discussions with their patient, the potential further risks of infrequent accessibility to DFU care if this is the case. Otherwise, we refer the reader back to earlier considerations for geographical remote populations for those patients that require to wear different offloading devices as part of their post-operative care in Recommendations 1-4. |
| Aboriginal & Torres Strait Islander people | In a similar manner to people in geographically remote locations, the panel also suggests that digital flexor tenotomy surgical offloading procedure may be impacted by infrequent access to DFU care for Aboriginal and Torres Strait Islander people. Additionally, as per previous recommendations, we suggest health professionals be cognisant of the possibility that Aboriginal and Torres Strait Islander people may feel a sense of ‘shame’ in having such surgery that may highlight they have a health issue, plus, any impending traditional meetings or ceremonies in which the patient needs to attend. Finally, the panel again strongly suggests for Aboriginal and Torres Strait Islander people it is very important to carefully discuss with the person the above anticipated benefits and risks of such procedures in the context of the patient’s personal circumstances and preferably to have those discussions in collaboration with a local Aboriginal and Torres Strait Islander health care worker. |
| Other sub-groups of interest | Similarly, to the previous surgical offloading recommendations, the panel also suggests that those patients with various levels of infection or ischaemia should instead consider using recommendations 7a, 7b and 7c. Further, the panel suggest that there is unlikely to be any benefit from performing a digital flexor tenotomy procedure in those patients with a rigid toe deformity and as such we suggest against using these procedures in this patient sub-group. Lastly, the panel refers the reader back to the other subgroup considerations in Recommendations 1-4 for the various offloading device treatments that may be considered for the patient to use post-operatively. |
| Monitoring & evaluation | Similar to the previous surgical offloading procedure recommendation the panel suggests organisations include in their formal monitoring systems options to be able to collect, monitor and analyse the impact of these surgical offloading procedures on their patients’ healing outcomes (1). Refer to Recommendation 5 for more details on these systems. |
| Future research priorities | The panel agreed with the IWGDF that with no previous controlled trials investigating this digital flexor tenotomy procedure, that robust controlled trials are needed to shed much more light on the (cost-)effectiveness of this promising surgical offloading intervention on healing and plantar pressure reduction (1). Furthermore, consideration should be given to performing these trials in both complicated non-healing ulcers and uncomplicated ulcers to determine if the more permanent surgical intervention provides more benefit than gold standard non-surgical offloading options. Lastly, it would be useful to consider also implementing such trials on this relatively simple procedure in Aboriginal and Torres Strait Islander populations and/or regions that are geographically remote. |

DFU; Diabetes-related foot ulcer; EtD: Evidence to decision; IWGDF:, International Working Group on the Diabetic Foot; TCC: Total contact cast

## OTHER ULCER TYPES AND LOCATIONS

#### **Recommendation 7A**

In a person with diabetes and a neuropathic plantar forefoot or midfoot ulcer with either mild infection or mild ischemia, consider using a non-removable knee-high offloading device to promote healing of the ulcer (Weak; Low).

**eTable B9:** Detailed considerations for Recommendation 7A

| Topic | Considerations |
| --- | --- |
| General implementation | The panel agreed with the IWGDF that either non-removable knee-high offloading devices can be used as the intervention for this recommendation, depending on the patient’s circumstances. However, the panel strongly suggests the reader also immediately enact the recommendations in the associated Australian DFD Guidelines for Infection and PAD to appropriately treat the patient’s mild infection and ischaemia, respectively (65, 66). Otherwise, we refer the reader to the same implementation considerations outlined in Recommendations 1, such as optimising the pressure offloading insole, using an evenup or similar on the contralateral shoe and providing patient-friendly written information on the benefits and risks of the devices to enable the patient to make an informed decision after weighing up the benefits and risks within the prism of their personal circumstances (1, 48). Furthermore, we suggest a mildly infected DFU and device is inspected at least twice each week until the mild infection is adequately resolved and once resolved refer to Recommendations 1. Lastly, if the infection or ischaemia deteriorates refer to Recommendations 7B and/or 7C below. |
| Contraindicated sub-groups | NA |
| Geographically remote people | The panel refers the reader to the same additional considerations for geographically remote populations at contained in Recommendations 1. Additionally, the panel highlights that if patients do not have at least twice weekly access to DFU care to inspect their infected DFU and review their device that until an infection is resolved then the clinician should consider a removable offloading device instead and in that case refer to Recommendation 2. |
| Aboriginal & Torres Strait Islander people | In addition to geographical remote considerations above, the panel refers the reader to the same additional considerations contained in Recommendations 1 for Aboriginal and Torres Strait Islander peoples. |
| Other sub-groups of interest | The panel refers the reader to the additional considerations contained in Recommendations 1 for other important subgroups. |
| Monitoring & evaluation | The same monitoring considerations as outlined in Recommendations 1-3 apply. Additionally, we strongly suggest that the offloading treatment be reviewed at the same time as it is recommended to monitor the infection or PAD treatment and changed in accordance with any change in infection or ischaemia category. Lastly, we suggest that infection and PAD categories are also collected as part of the routine patient characteristics captured and monitored within organisational monitoring systems to enable monitoring of patients with complications to ensure they are receiving recommended offloading (33, 35). |
| Future research priorities | The panel suggests the same future research considerations outlined in Recommendation 1-3 are also applicable to this recommendation, including future Australian trials on different methods or makes of custom-made and pre-fabricated removable offloading devices to determine which are most (cost-) effective on healing, plantar pressure reduction, weight-bearing activity reduction, adverse events, patient satisfaction and adherence (1, 4), and specifically also within geographically remote and Aboriginal and Torres Strait Islander populations in the context of infection and or ischemia. |

DFU; Diabetes-related foot ulcer; EtD: Evidence to decision; IWGDF:, International Working Group on the Diabetic Foot; TCC: Total contact cast

**Recommendation 7B**

In a person with diabetes and a neuropathic plantar forefoot or midfoot ulcer with both mild infection and mild ischemia, or with either moderate infection or moderate ischaemia, consider using a removable knee-high offloading device to promote healing of the ulcer. (Weak; Low).

**eTable B10:** Detailed considerations for Recommendation 7B

| Topic | Considerations |
| --- | --- |
| General implementation | The panel agreed with the IWGDF that a removable knee‐high offloading device is often the most appropriate solution for circumstances when a patient with a DFU has a moderate infection or moderate ischaemia, as these ulcers still require optimal plantar pressure offloading, but also require frequent inspection and review. Therefore, we refer the reader the implementation considerations contained in Recommendation 2 when implementing removable knee-high offloading devices. Furthermore, we again highlight the critical importance of also immediately enacting the recommendations in the associated Australian DFD Guidelines for Infection and PAD to appropriately treat the patient’s moderate infection and ischaemia, respectively [REF], and as per Recommendation 7A highlight the need for these complicated DFU to be inspected multiple times each week until resolved. Lastly, if the infection or ischaemia improves, we refer the reader to Recommendations 1 and if deteriorates to Recommendations 7C below |
| Contraindicated sub-groups | NA |
| Geographically remote people | The panel refers the reader to the same additional considerations for geographically remote populations as contained in Recommendation 7A, in addition to those considerations for geographically remote people contained in the associated Australian DFD Guidelines for Infection and PAD |
| Aboriginal & Torres Strait Islander people | The panel refers the reader to the same additional considerations for Aboriginal and Torres Strait Islander peoples as contained in Recommendation 7A, in addition to those considerations for Aboriginal and Torres Strait Islander peoples contained in the associated Australian DFD Guidelines for Infection and PAD. |
| Other sub-groups of interest | The panel refers the reader to the additional considerations contained in Recommendation 7A for other important subgroups, in addition to those considerations for other important subgroups contained in the associated Australian DFD Guidelines for Infection and PAD. The panel also highlights their agreeance with IWGDF that patients with moderate-to-severe infections or moderate-to-severe ischaemia should not be prescribed non-removable knee-high offloading devices. |
| Monitoring & evaluation | The same monitoring considerations as outlined in Recommendations 1-3 apply. Additionally, we strongly suggest that the offloading treatment be reviewed at the same time as it is recommended to monitor the infection or PAD treatment and changed in accordance with any change in infection or ischaemia category. Lastly, we suggest that infection and PAD categories are also collected as part of the routine patient characteristics captured and monitored within organisational monitoring systems to enable monitoring of patients with complications to ensure they are receiving recommended offloading (33, 35). |
| Future research priorities | The panel suggests the same future research considerations outlined in Recommendation 1-3 are also applicable to this recommendation, including future Australian trials on different methods or makes of custom-made and pre-fabricated removable offloading devices to determine which are most (cost-) effective on healing, plantar pressure reduction, weight-bearing activity reduction, adverse events, patient satisfaction and adherence (1, 4), and specifically also within geographically remote and Aboriginal and Torres Strait Islander populations in the context of infection and or ischemia. |

DFU; Diabetes-related foot ulcer; EtD: Evidence to decision; IWGDF:, International Working Group on the Diabetic Foot; TCC: Total contact cast

**Recommendation 7C**

In a person with diabetes and a neuropathic plantar forefoot or midfoot ulcer with both moderate infection and moderate ischaemia, or with either severe infection or severe ischemia, primarily address the infection and/or ischemia, and consider using a removable offloading intervention based on the patient’s functioning, ambulatory status and activity level, to promote healing of the ulcer (Weak; Low).

**eTable B11:** Detailed considerations for Recommendation 7C

| Topic | Considerations |
| --- | --- |
| General implementation | The panel agreed with the IWGDF that a removable offloading device, either knee-high or ankle-high is often the most appropriate solution for circumstances when a patient with a DFU has a severe infection or severe ischaemia, as these ulcers still require plantar pressure offloading, but that the infection or ischaemia should be the primary cause for concern. Therefore, we highlight the paramount importance of immediately enacting the recommendations in the associated Australian DFD Guidelines for Infection and PAD to appropriately treat the patient’s severe infection and ischaemia first and foremost, respectively [REF], before clinicians turn their attention to offloading treatment. However, we agree with the IWGDF and highlight that these patients do need offloading immediately after prescribing infection or ischaemia treatment plans to reduce plantar pressure and assist with a DFU environment to resolve infection. Therefore, we refer the reader the implementation considerations contained in Recommendation 2 and 3 when implementing removable offloading devices. Lastly, if the infection or ischaemia improves, we refer the reader to Recommendations to Recommendations 7A and 7B above. |
| Contraindicated sub-groups | NA |
| Geographically remote people | The panel refers the reader to the same additional considerations for geographically remote populations as contained in Recommendation 7A, in addition to those considerations for geographically remote people contained in the associated Australian DFD Guidelines for Infection and PAD. |
| Aboriginal & Torres Strait Islander people | The panel refers the reader to the same additional considerations for Aboriginal and Torres Strait Island peoples as contained in Recommendation 7A, in addition to those considerations for Aboriginal and Torres Strait Islander peoples contained in the associated Australian DFD Guidelines for Infection and PAD. |
| Other sub-groups of interest | The panel refers the reader to the additional considerations contained in Recommendation 7A for other important subgroups, in addition to those considerations for other important subgroups contained in the associated Australian DFD Guidelines for Infection and PAD. The panel again highlights their agreeance with IWGDF that patients with moderate-to-severe infections or moderate-to-severe ischaemia should not be prescribed non-removable knee-high offloading devices. |
| Monitoring & evaluation | The same monitoring considerations as outlined in Recommendations 1-3 apply. Additionally, we strongly suggest that the offloading treatment be reviewed at the same time as it is recommended to monitor the infection or PAD treatment and changed in accordance with any change in infection or ischaemia category. Lastly, we suggest that infection and PAD categories are also collected as part of the routine patient characteristics captured and monitored within organisational monitoring systems to enable monitoring of patients with complications to ensure they are receiving recommended offloading (33, 35). |
| Future research priorities | The panel suggests the same future research considerations outlined in Recommendation 1-3 are also applicable to this recommendation, including future Australian trials on different methods or makes of custom-made and pre-fabricated removable offloading devices to determine which are most (cost-) effective on healing, plantar pressure reduction, weight-bearing activity reduction, adverse events, patient satisfaction and adherence (1, 4), and specifically also within geographically remote and Aboriginal and Torres Strait Islander populations in the context of infection and or ischemia. |

DFU; Diabetes-related foot ulcer; EtD: Evidence to decision; IWGDF:, International Working Group on the Diabetic Foot; TCC: Total contact cast

**Recommendation 8**

In a person with diabetes and a neuropathic plantar heel ulcer, consider using a knee-high offloading device or other offloading intervention that effectively reduces plantar pressure on the heel and is tolerated by the patient, to promote healing of the ulcer (Weak; Low).

**eTable B12:** Detailed considerations for Recommendation 8

| Topic | Considerations |
| --- | --- |
| General implementation | The panel agreed with the IWGDF that either non-removable or removable knee-high offloading devices can be used as the intervention for this recommendation, depending on the patient’s circumstances. However, as previously outlined in Recommendation 2, if choosing a removable knee-high offloading device, the patient should be made fully aware of the importance of adhering to wearing the device at all times or as much as possible. Otherwise, when considering a knee-high offloading device, we refer the reader to the same implementation considerations outlined in Recommendations 1-2, such as optimising the pressure offloading insole, using an evenup or similar on the contralateral shoe and providing patient-friendly written information on the benefits and risks of the devices to enable the patient to make an informed decision after weighing up the benefits and risks within the prism of their personal circumstances. Furthermore, we suggest because of the lack of available evidence for plantar rearfoot ulcers that it is even more important to use, if available, validated plantar pressure measurements to objectively measure and guide the modification of the insole, evenup and device to further optimise the reduction of plantar pressure at the rearfoot DFU site (1, 48). There is no evidence for wheelchair, electric scooters or knee scooters however the panel should recommend consideration of these devices given the poor prognosis for non-healing, heal ulcers. |
| Contraindicated sub-groups | The same contraindications as outlined in Recommendations 1-2 also apply, depending on the knee-high offloading device chosen. |
| Geographically remote people | In terms of considerations to use this recommendation in geographically remote people, the panel refers the reader to consider those same geographical remote considerations outlined in Recommendations 1-2. |
| Aboriginal & Torres Strait Islander people | In terms of considerations to use this recommendation in Aboriginal and Torres Strait Islander people, the panel refers the reader to consider those same Aboriginal and Torres Strait Islander people considerations outlined in Recommendations 1-2. |
| Other sub-groups of interest | In terms of considerations to use this recommendation in other subgroups of importance, the panel refers the reader to consider those same other subgroup considerations outlined in Recommendations 1-2, including those with infection, ischaemia or confirmed falls risk. |
| Monitoring & evaluation | In terms of monitoring considerations, the panel again suggests referring to those same monitoring considerations outlined in Recommendations 1-2, including that organisations incorporate in their formal key clinical performance indicator monitoring systems, an item to objectively measure the proportion of their patients with DFU that are prescribed knee-high offloading devices of those eligible (1). In this case we also suggest that organisations consider adding the ability to identify the ulcer site in monitoring systems to enable a determination of the local outcomes of plantar rearfoot ulcers when using knee-high offloading devices or other offloading treatments. |
| Future research priorities | The panel also agreed with the IWGDF that many more trials are required to test the effectiveness of different offloading interventions on the effectiveness to heal neuropathic plantar rearfoot DFU to more definitely determine the best intervention in these circumstances (1, 4). Although the patient population in this case are people with rearfoot ulcers, we again refer the reader to Recommendations 1-2 in terms of the interventions, controls and outcomes that should be measured in such future national and international trials in this important patient population with challenging plantar rearfoot ulcers. |

DFU; Diabetes-related foot ulcer; EtD: Evidence to decision; IWGDF:, International Working Group on the Diabetic Foot; TCC: Total contact cast

**Recommendation 9**

In a person with diabetes and a non-plantar foot ulcer, use a removable offloading device, medical grade footwear, felted foam, toe spacers or orthoses, depending on the type and location of the foot ulcer, rather than no offloading intervention to promote healing of the ulcer and to prevent further ulceration (Strong; Very Low).

**eTable B13:** Detailed considerations for Recommendation 9

| Topic | Considerations |
| --- | --- |
| General implementation | The panel agreed with the IWGDF that a range of offloading devices are likely suitable for offloading non-plantar DFU. This includes removable offloading devices, footwear modifications, toe spacers, or orthoses and others, depending on the type and location of the foot ulcer, to promote healing of the ulcer. Therefore, until new evidence becomes available and depending on the location of the non-plantar ulcer, the panel recommend that various modalities can be considered. However, the panel felt that standard or therapeutic footwear alone may not be suitable for offloading non-plantar ulcers. However, the panel felt that there was currently insufficient evidence to guide practice (1). Otherwise, we refer the reader to the same implementation considerations outlined in Recommendation 1A to consider in these instances as well, such as optimising the pressure offloading insole, using an evenup or similar on the contralateral shoe and providing patient-friendly written information on the benefits and risks of the devices to enable the patient to make an informed decision based on weighing up the benefits and risks within the prism of their personal circumstances. As per the other recommendations, health professionals should consider providing to each patient the national clinical pathway provided (Figure 1) and locally developed patient-friendly written information on the likely benefits, risks and 24-hour emergency contact information for each device (based on the desirable and undesirable) stated in earlier recommendations (1, 48). |
| Contraindicated sub-groups | The same contraindications in Recommendations 2-5 apply, depending on the removable non-surgical offloading intervention chosen. |
| Geographically remote people | Please see above Recommendations 2-5 for more details on those implementation considerations for geographically remote people that should be considered for this recommendation as well. In addition, we would highlight that removable devices may be more favourable in those from geographically remote areas (5). |
| Aboriginal & Torres Strait Islander people | Please see above Recommendations 2-5 for more details on those implementation considerations for Aboriginal and Torre Strait Islander people that should be considered for this recommendation as well. |
| Other sub-groups of interest | Please see above Recommendations 2-5 for more details on other subgroup considerations for this range of devices that should be considered for this recommendation as well. |
| Monitoring & evaluation | The panel refers the reader to the same monitoring considerations contained in Recommendation 1-5 for this recommendation as well. |
| Future research priorities | Given there is an absolute lack of studies reporting on the offloading requirements for non-plantar DFU, further studies are urgently needed in this area including controlled trials which are adequately powered and appropriately designed. |

DFU; Diabetes-related foot ulcer; EtD: Evidence to decision; IWGDF:, International Working Group on the Diabetic Foot; TCC: Total contact cast

**REFERENCES**

1. Bus SA, Armstrong DG, Gooday C, Jarl G, Caravaggi C, Viswanathan V, et al. Guidelines on offloading foot ulcers in persons with diabetes (IWGDF 2019 update). Diabetes/Metabolism Research and Reviews. 2020;36(S1):e3274.

2. Lazzarini PA, van Netten JJ, Fitridge R, Griffiths I, Kinnear EM, Malone M, et al. Pathway to ending avoidable diabetes-related amputations in Australia. The Medical Journal Of Australia. 2018;209(7):288-90.

3. Jeffcoate WJ, Bus SA, Game FL, Hinchliffe RJ, Price PE, Schaper NC. Reporting standards of studies and papers on the prevention and management of foot ulcers in diabetes: required details and markers of good quality. The Lancet Diabetes & Endocrinology. 2016;4(9):781-8.

4. Lazzarini PA, Jarl G, Gooday C, Viswanathan V, Caravaggi CF, Armstrong DG, et al. Effectiveness of offloading interventions to heal foot ulcers in persons with diabetes: a systematic review. Diabetes/Metabolism Research and Reviews. 2020;36(S1):e3275.

5. Health Quality O. Fibreglass total contact casting, removable cast walkers, and irremovable cast walkers to treat diabetic neuropathic foot ulcers: A health technology assessment. Ontario Health Technology Assessment Series. 2017;17(12):1-124.

6. National Association of Diabetes Centres (NADC) and Australian Diabetes Society (ADS). NADC Collaborative Interdisciplinary Diabetes High Risk Foot Services Standards Version 1.1 Sydney, Australia: National Association of Diabetes Centres; 2018 [Available from: <https://nadc.net.au/national-standards/>.

7. Crews RT, Shen BJ, Campbell L, Lamont PJ, Boulton AJ, Peyrot M, et al. Role and Determinants of Adherence to Off-loading in Diabetic Foot Ulcer Healing: A Prospective Investigation. Diabetes Care. 2016;39(8):1371-7.

8. Crews RT, Candela J. Decreasing an Offloading Device’s Size and Offsetting Its Imposed Limb Length Discrepancy Lead to Improved Comfort and Gait. Diabetes Care. 2018:dc172584.

9. Lazzarini PA, Jarl G, Gooday C, Viswanathan V, Caravaggi CF, Armstrong DG, et al. Effectiveness of offloading interventions to heal foot ulcers in persons with diabetes: a systematic review. Diabetes Metab Res Rev. 2020;36 Suppl 1:e3275.

10. Bus SA, van Netten JJ, Kottink AI, Manning EA, Spraul M, Woittiez AJ, et al. The efficacy of removable devices to offload and heal neuropathic plantar forefoot ulcers in people with diabetes: a single-blinded multicentre randomised controlled trial. Int Wound J. 2018;15(1):65-74.

11. Lavery LA, Higgins KR, La Fontaine J, Zamorano RG, Constantinides GP, Kim PJ. Randomised clinical trial to compare total contact casts, healing sandals and a shear-reducing removable boot to heal diabetic foot ulcers. Int Wound J. 2015;12(6):710-5.

12. de Oliveira AL, Moore Z. Treatment of the diabetic foot by offloading: a systematic review. J Wound Care. 2015;24(12):560, 2-70.

13. Armstrong DG, Lavery LA, Wrobel JS, Vileikyte L. Quality of life in healing diabetic wounds: does the end justify the means? Journal of Foot & Ankle Surgery. 2008;47(4):278-82.

14. Dumont IJ, Lepeut MS, Tsirtsikolou DM, Popielarz SM, Cordonnier MM, Fayard AJ, et al. A proof-of-concept study of the effectiveness of a removable device for offloading in patients with neuropathic ulceration of the foot: the Ransart boot. Diabet Med. 2009;26(8):778-82.

15. Raspovic A, Landorf KB, Gazarek J, Stark M. Reduction of peak plantar pressure in people with diabetes-related peripheral neuropathy: an evaluation of the DH Pressure Relief Shoe™. J Foot Ankle Res. 2012;5(1):25.

16. Birke JA, Pavich MA, Patout CA, Jr., Horswell R. Comparison of forefoot ulcer healing using alternative off-loading methods in patients with diabetes mellitus. Adv Skin Wound Care. 2002;15(5):210-5.

17. Chantelau E, Breuer U, Leisch AC, Tanudjaja T, Reuter M. Outpatient treatment of unilateral diabetic foot ulcers with 'half shoes'. Diabet Med. 1993;10(3):267-70.

18. Fleischli JG, Lavery LA, Vela SA, Ashry H, Lavery DC. 1997 William J. Stickel Bronze Award. Comparison of strategies for reducing pressure at the site of neuropathic ulcers. Journal Of The American Podiatric Medical Association. 1997;87(10):466-72.

19. Götz J, Lange M, Dullien S, Grifka J, Hertel G, Baier C, et al. Off-loading strategies in diabetic foot syndrome-evaluation of different devices. Int Orthop. 2017;41(2):239-46.

20. Bus SA, van Deursen RW, Kanade RV, Wissink M, Manning EA, van Baal JG, et al. Plantar pressure relief in the diabetic foot using forefoot offloading shoes. Gait Posture. 2009;29(4):618-22.

21. Caravaggi P, Giangrande A, Berti L, Lullini G, Leardini A. Pedobarographic and kinematic analysis in the functional evaluation of two post-operative forefoot offloading shoes. Journal of Foot and Ankle Research. 2015;8(1):59.

22. Fernando ME, Crowther RG, Lazzarini PA, Sangla KS, Wearing S, Buttner P, et al. Gait in People With Nonhealing Diabetes-Related Plantar Ulcers. Physical Therapy. 2019;99(12):1602-15.

23. Crews RT, Yalla SV, Fleischer AE, Wu SC. A growing troubling triad: diabetes, aging, and falls. Journal of aging research. 2013;2013:342650-.

24. Mueller MJ, Diamond JE, Sinacore DR, Delitto A, Blair VP, Drury DA, et al. Total contact casting in treatment of diabetic plantar ulcers. Controlled clinical trial. Diabetes care. 1989;12(6):384-8.

25. Miyan Z, Ahmed J, Zaidi SI, Ahmedani MY, Fawwad A, Basit A. Use of locally made off-loading techniques for diabetic plantar foot ulcer in Karachi, Pakistan. Int Wound J. 2014;11(6):691-5.

26. Caravaggi C, Faglia E, De Giglio R, Mantero M, Quarantiello A, Sommariva E, et al. Effectiveness and safety of a nonremovable fiberglass off-bearing cast versus a therapeutic shoe in the treatment of neuropathic foot ulcers: a randomized study. Diabetes Care. 2000;23(12):1746-51.

27. Morona JK, Buckley ES, Jones S, Reddin EA, Merlin TL. Comparison of the clinical effectiveness of different off-loading devices for the treatment of neuropathic foot ulcers in patients with diabetes: a systematic review and meta-analysis. Diabetes/metabolism research and reviews. 2013;29(3):183-93.

28. van Netten JJ, Lazzarini PA, Armstrong DG, Bus SA, Fitridge R, Harding K, et al. Diabetic Foot Australia guideline on footwear for people with diabetes. Journal of Foot and Ankle Research. 2018;11(1):2.

29. Zimny S, Schatz H, Pfohl U. The effects of applied felted foam on wound healing and healing times in the therapy of neuropathic diabetic foot ulcers. Diabetic Medicine. 2003;20(8):622-5.

30. Pabon-Carrasco M, Juarez-Jimenez JM, Reina-Bueno M, Cohena-Jimenez M. Behavior of provisional pressure-reducing materials in diabetic foot. J Tissue Viability. 2016;25(2):143-9.

31. Raspovic A, Waller K, Wong WM. The effectiveness of felt padding for offloading diabetes-related foot ulcers, at baseline and after one week of wear. Diabetes Res Clin Pract. 2016;121:166-72.

32. Nubé VL, Molyneaux L, Bolton T, Clingan T, Palmer E, Yue DK. The use of felt deflective padding in the management of plantar hallux and forefoot ulcers in patients with diabetes. Foot. 2006;16(1):38-43.

33. Raspovic A, Landorf KB. A survey of offloading practices for diabetes-related plantar neuropathic foot ulcers. Journal of Foot and Ankle Research. 2014;7(1):1-8.

34. Quinton TR, Lazzarini PA, Boyle FM, Russell AW, Armstrong DG. How do Australian podiatrists manage patients with diabetes? The Australian diabetic foot management survey. J Foot Ankle Res. 2015;8:16.

35. Mueller MJ, Sinacore DR, Hastings MK, Strube MJ, Johnson JE. Effect of Achilles tendon lengthening on neuropathic plantar ulcers. A randomized clinical trial. The Journal Of Bone And Joint Surgery American Volume. 2003;85-A(8):1436-45.

36. Dallimore SM, Kaminski MR. Tendon lengthening and fascia release for healing and preventing diabetic foot ulcers: a systematic review and meta-analysis. Journal Of Foot And Ankle Research. 2015;8:33-.

37. Allam AM. Impact of Achilles tendon lengthening (ATL) on the diabetic plantar forefoot ulceration. Egypt J Plast Reconstr Surg. 2006;30:43-8.

38. Maluf KS, Mueller MJ, Strube MJ, Engsberg JR, Johnson JE. Tendon Achilles lengthening for the treatment of neuropathic ulcers causes a temporary reduction in forefoot pressure associated with changes in plantar flexor power rather than ankle motion during gait. Journal Of Biomechanics. 2004;37(6):897-906.

39. Laborde JM. Neuropathic plantar forefoot ulcers treated with tendon lengthenings. Foot Ankle Int. 2008;29(4):378-84.

40. Laborde JM. Midfoot ulcers treated with gastrocnemius-soleus recession. Foot Ankle Int. 2009;30(9):842-6.

41. Holstein P, Lohmann M, Bitsch M, Jorgensen B. Achilles tendon lengthening, the panacea for plantar forefoot ulceration? Diabetes Metab Res Rev. 2004;20 Suppl 1:S37-40.

42. La Fontaine J, Brown D, Adams M, VanPelt M. New and recurrent ulcerations after percutaneous achilles tendon lengthening in transmetatarsal amputation. J Foot Ankle Surg. 2008;47(3):225-9.

43. Piaggesi A, Schipani E, Campi F, Romanelli M, Baccetti F, Arvia C, et al. Conservative surgical approach versus non-surgical management for diabetic neuropathic foot ulcers: a randomized trial. Diabetic Medicine: A Journal Of The British Diabetic Association. 1998;15(5):412-7.

44. Bonanno DR, Gillies EJ. Flexor Tenotomy Improves Healing and Prevention of Diabetes-Related Toe Ulcers: A Systematic Review. J Foot Ankle Surg. 2017;56(3):600-4.

45. Scott JE, Hendry GJ, Locke J. Effectiveness of percutaneous flexor tenotomies for the management and prevention of recurrence of diabetic toe ulcers: a systematic review. J Foot Ankle Res. 2016;9:25.

46. Smith SE, Miller J. The Safety and Effectiveness of the Percutaneous Flexor Tenotomy in Healing Neuropathic Apical Toe Ulcers in the Outpatient Setting. Foot Ankle Spec. 2020;13(2):123-31.

47. Jeffcoate W, Game F, Turtle-Savage V, Musgrove A, Price P, Tan W, et al. Evaluation of the effectiveness and cost-effectiveness of lightweight fibreglass heel casts in the management of ulcers of the heel in diabetes: a randomised controlled trial. Health Technol Assess. 2017;21(34):1-92.

48. Jarl G, Gooday C, Lazzarini PA, Bus SA. Practical considerations for implementing the new IWGDF guideline for offloading diabetic foot ulcers. The Diabetic Foot Journal. 2020;23(2):34-40.

49. Ahmed S, Barwick A, Butterworth P, Nancarrow S. Footwear and insole design features that reduce neuropathic plantar forefoot ulcer risk in people with diabetes: a systematic literature review. Journal of Foot and Ankle Research. 2020;13(1):30.

50. (ACSQHC) ACoSaQiHC. Preventing Falls and Harm From Falls in Older People: Best Practice Guidelines for Australian Community Care. In: ACSQHC, editor. Sydney, Australia: Commonwealth of Australia; 2009.

51. Australian Bureau of Statistics (ABS). Australian Standard Geographical Classification (ABS Cat No. 1216.0). Canberra: ABS; 2010.

52. Services Australia. Your guide to Medicare for Indigenous health services: April 2021. Canberra, Australia; 2021 7 May 2021.

53. Lazzarini PA VNJ, Fitridge R, Kinnear E, Malone M, Perrin BM, Prentice J, Wraight PR. Australian Diabetic Foot Ulcer Minimum Dataset Dictionary. Brisbane: Diabetic Foot Australia, Wound Management Innovation CRC; 2016.

54. Lazzarini PA, Ng V, Kinnear EM, Kamp MC, Kuys SS, Hurst C, et al. The Queensland high risk foot form (QHRFF) - is it a reliable and valid clinical research tool for foot disease? Journal Of Foot And Ankle Research. 2014;7(1):7-.

55. Schulz KF, Altman DG, Moher D. CONSORT 2010 Statement: updated guidelines for reporting parallel group randomised trials. BMJ. 2010;340:c332.

56. Caputo GM, Ulbrecht JS, Cavanagh PR. The total contact cast: a method for treating neuropathic diabetic ulcers. American family physician. 1997;55(2):605-11, 15-6.

57. Tamir E, Heim M, Siev-Ner I. Removable fiberglass de-loading cast for the management of neuropathic plantar ulceration of the foot. The Israel Medical Association journal : IMAJ. 2005;7(8):507-10.

58. Piaggesi A, Macchiarini S, Rizzo L, Palumbo F, Tedeschi A, Nobili LA, et al. An off-the-shelf instant contact casting device for the management of diabetic foot ulcers: a randomized prospective trial versus traditional fiberglass cast. Diabetes Care. 2007;30(3):586-90.

59. Lazzarini PA, Crews RT, van Netten JJ, Bus SA, Fernando ME, Chadwick PJ, et al. Measuring Plantar Tissue Stress in People With Diabetic Peripheral Neuropathy: A Critical Concept in Diabetic Foot Management. Journal of Diabetes Science and Technology. 2019;13(5):869-80.

60. Lukaszyk C, Harvey L, Sherrington C, Keay L, Tiedemann A, Coombes J, et al. Risk factors, incidence, consequences and prevention strategies for falls and fall-injury within older indigenous populations: a systematic review. Australian and New Zealand Journal of Public Health. 2016;40(6):564-8.

61. Jarl G. Methodological considerations of investigating adherence to using offloading devices among people with diabetes. Patient Prefer Adherence. 2018;12:1767-75.

62. Charles J. An investigation into the foot health of Aboriginal and Torres Strait Islander peoples: a literature review. Australian Indigenous HealthBulletin 15(3) Retrieved [access date] from <http://healthbulletinorgau/articles/an-investigati>…erature-review/. 2015.

63. Hamilton EJ, Scheepers J, Ryan H, Perrin BM, Charles J, Cheney J, et al. Australian guideline on wound classification of diabetes-related foot ulcers: Part of the 2021 Australian evidence-based guidelines for diabetes-related foot disease. Brisbane, Australia: Diabetes Feet Australia, Australian Diabetes Society; 2021.

64. Chen P, Carville K, Swanson T, Lazzarini PA, Charles J, Cheney J, et al. Australian guideline on wound healing interventions to enhance healing of foot ulcers: Part of the 2021 Australian evidence-based guidelines for diabetes-related foot disease. Brisbane, Australia: Diabetes Feet Australia, Australian Diabetes Society; 2021.

65. Chuter VH, Quigley F, Tosenovsky P, Ritter JC, Charles J, Cheney J, et al. Australian guideline on diagnosis and management of peripheral artery disease: Part of the 2021 Australian evidence-based guidelines for diabetes-related foot disease. Brisbane, Australia: Diabetes Feet Australia, Australian Diabetes Society; 2021.

66. Commons RJ, Charles J, Cheney J, Lynar SA, Malone M, Raby E, et al. Australian guideline on management of diabetes-related foot infection: Part of the 2021 Australian evidence-based guidelines for diabetes-related foot disease. Brisbane, Australia: Diabetes Feet Australia, Australian Diabetes Society; 2021.

67. Sheehan P, Jones P, Caselli A, Giurini JM, Veves A. Percent change in wound area of diabetic foot ulcers over a 4-week period is a robust predictor of complete healing in a 12-week prospective trial. Diabetes Care. 2003;26(6):1879-82.

68. Independent Hospital Pricing Authority (IHPA). Chronicle of The International Statistical Classification of Diseases and Related Health Problems, Tenth Revision, Australian Modification (ICD-10-AM): First Edition to Tenth Edition, 2017. In: IHPA, editor. Darlinghurst, NSW: IHPA; 2017.

69. Queensland Department of Health. Queensland Hospital Admitted Patient Data Collection (QHAPDC) Manual 2019-2020 Version 1.2. In: Branch SS, editor. Brisbane, Queensland: Queensland Health; 2019.
